# Functional anatomy, jaw mechanisms, and feeding behavior of *Dunkleosteus terrelli* (Placodermi, Arthrodira)

**DOI:** 10.1002/ar.70075

**Published:** 2025-11-20

**Authors:** Russell K. Engelman, Robert K. Carr, Kate Trinajstic, Zerina Johanson, Oleg A. Lebedev

**Affiliations:** ^1^ Department of Biology Case Western Reserve University Cleveland Ohio USA; ^2^ Cleveland Museum of Natural History Cleveland Ohio USA; ^3^ School of Molecular and Life Sciences Curtin University Perth Western Australia Australia; ^4^ Earth and Planetary Sciences Western Australian Museum Perth Western Australia Australia; ^5^ Natural History Museum London UK; ^6^ A.A. Borissiak Palaeontological Institute of the Russian Academy of Sciences Moscow Russia

**Keywords:** biomechanics, Cleveland shale, Devonian, gnathostome, paleobiology, paleoecology

## Abstract

The comparative and functional anatomy of the cleaver‐like jaws of the Late Devonian arthrodire *Dunkleosteus terrelli* remains poorly understood, despite significant advances in our understanding of arthrodire anatomy. Here, we re‐investigate the jaw morphology of *D. terrelli* from a comparative anatomical perspective. Rare cartilaginous elements, preserved attachment sites, and comparisons with other arthrodires suggest the skull of *Dunkleosteus* contained significantly more cartilage than previously assumed, with many major jaw muscle attachments and cranial articulations located on cartilages. The *m. adductor mandibulae* likely did not originate on the skull roof, *contra* previous reconstructions, but instead either partially or entirely on the palatoquadrate, which in *D. terrelli* has an unusual, asymmetrical shape. A large external groove spanning the suborbital plate suggests the presence of a large *m. preorbitalis* originating on the enlarged ethmoid ossification, likely playing a role in jaw closure. The submarginal plate is unusually elongate compared to other arthrodires and lacks bony connections to the skull roof or the rest of the cheek, suggesting a specialized, loosely consolidated posterior cheek. The symphyseal cartilages of the lower jaw have a well‐developed medial facet, which would have created a stable joint with limited symphyseal flexibility. Many features of *D. terrelli* appear to reflect broader evolutionary trends characterizing dunkleosteoids, and some appear to be convergent specializations for macropredation shared with some aspinothoracidans. Prior claims of suction feeding in *D. terrelli* are questionable on comparative anatomical and ecological grounds, and previous estimates of bite force need to be re‐examined based on the revised myology.

## Graphical abstract

A new musculoskeletal reconstruction and revision of the cranio‐mandibular anatomy of the Devonian arthrodire placoderm *Dunkleosteus terrelli* from a comparative and functional anatomical perspective. *Dunkleosteus* is a specialized arthrodire with many specializations for feeding on large vertebrates, and many of its features are part of broader evolutionary trends within Arthrodira.

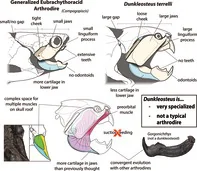

**Abbreviations**
AMNH: American Museum of Natural History, New York, USA
ANU: Australian National University, Canberra, Australia
CMC: Cincinnati Museum Center, Cincinnati, USA
CMNH: Cleveland Museum of Natural History, Cleveland, USA
LDUCZ: Grant Museum of Zoology, University College, London, UK
MB.f: fossil fish collections, Museum für Naturkunde, Berlin, Germany
MHNM: Musée d'Histoire Naturelle de Miguasha, Quebec, Canada
NHMUK: The Natural History Museum (London), London, UK
NMV: Museum Victoria, Melbourne, Australia
RMNH.PISC: former collections of the Rijksmuseum van Natuurlijke Historiche in the Naturalis Biodiversity Center, Leiden, the Netherlands
ROM: Royal Ontario Museum, Toronto, Canada
UALVP: University of Alberta Laboratory of Vertebrate Paleontology, Calgary, Canada
USNM: Smithsonian National Museum of Natural History, Washington D.C., USA
WAM: Western Australian Museum, Perth, Australia
ZMA.PISC: former collections of the Zoölogisch Museum Amsterdam in the Naturalis Biodiversity Center, Leiden, the Netherlands

## 1 INTRODUCTION

The gnathal plates of Late Devonian dunkleosteid arthrodires like *Dunkleosteus terrelli* are some of the most imposing mouthparts in the animal kingdom. With their fang‐like odontoids (sensu Lebedev et al., 2023) and bladed margins made of sharpened bone, these structures have fascinated researchers for more than a century (Newberry, 1873, 1875). The function of the mouthparts of *Dunkleosteus* has been the subject of several studies (Anderson, 2009, 2010; Anderson & Westneat, 2007; Dunkle & Bungart, 1946; Heintz, 1931a, 1932; Hussakof, 1906; Snively et al., 2010), but interpretation has been difficult due to the challenging preservation of the fossil material and the lack of close living relatives to serve as functional analogs. Outside of some exceptional examples like the Gogo Formation of Western Australia (Miles, 1970; Trinajstic, Briggs, & Long, 2022; Trinajstic, Long, et al., 2022) and the anti‐Atlas region of Morocco (e.g., Rücklin, 2011; Rücklin et al., 2015), many arthrodire fossils—including most specimens of *Dunkleosteus* (Heintz, 1932, pp. 116–122)—are preserved in a crushed and flattened state, requiring significant interpretation to reconstruct their original anatomy. Additionally, while the dermal armor and gnathal plates of arthrodires readily preserve, many elements of the cranial skeleton—including the palatoquadrate, hyomandibula, braincase, branchial arches, Meckel's cartilage, and articular surfaces of the jaw articulation and mandibular symphysis—are largely cartilaginous (with at most only a thin outer layer of perichondral bone), and thus rarely found in the fossil record (see Carr et al., 2009; Gardiner & Miles, 1990; Goujet, 1975; Hu et al., 2017; Jobbins et al., 2024; Stensiö, 1969; Young, 1979 for exceptions). These factors tend to make the anatomy of arthrodires beyond the dermal skeleton challenging to interpret (see, e.g., Adams, 1919; Branson, 1908; Heintz, 1931a, 1931b, 1932, 1938; Miles & Westoll, 1968; Newberry, 1873, 1875; Stensiö, 1963).

The musculoskeletal anatomy of *Dunkleosteus terrelli*, in particular, is in need of revision. The last major revision of this taxon's cranial anatomy was published by Heintz (1931a, 1932), with Dunkle and Bungart (1942a, 1946) adding a few additional observations on the gnathal plates and lateral consolidated region of the skull roof. Notably, Heintz (1932, pp. 115–116, 123) did not consider the well‐preserved *D. terrelli* material from the Cleveland Museum of Natural History, Cleveland, USA (CMNH) in his study (Heintz, 1932, p. 116), instead focusing on material from the American Museum of Natural History (AMNH), which is less well‐preserved and largely consists of isolated plates (Dean, 1909; Engelman, 2024, p. 3; Heintz, 1932, p. 116; Hussakof, 1905, p. 27; 1906). This means the CMNH *Dunkleosteus* hypodigm (sensu Newell, 1949; Simpson, 1940), which includes multiple near‐complete, three‐dimensionally mounted dermal skeletons, has remained almost undescribed from an anatomical perspective (Engelman, 2024, p. 3), especially with regards to the dermal armor and cranial anatomy. More recent works on the cranio‐mandibular anatomy of *Dunkleosteus* have primarily examined functional anatomy through either biomechanical modeling (Anderson, 2010; Anderson & Westneat, 2007, 2009) or finite element analysis (Snively et al., 2010), largely using these prior anatomical models as a starting point.

Significant advances in arthrodire comparative anatomy have been made since the foundational works of Heintz (1932) and Dunkle and Bungart (1942a, 1946), including Stensiö's monographs on arthrodiran morphology (Stensiö, 1959, 1963), Miles' works on arthrodire functional anatomy (Miles, 1967, 1969), and extensive monograph on *Coccosteus* (Miles & Westoll, 1968), and the discovery of well‐preserved arthrodires from the early Late Devonian (Frasnian) Gogo Formation of Western Australia (Miles, 1970, p. 12, Trinajstic, Briggs, & Long, 2022, and see specimens in Dennis‐Bryan, 1987; Dennis & Miles, 1981; Gardiner & Miles, 1990, 1994; Long, 1988, 1995; Miles & Dennis, 1979). The arthrodires from the Gogo Formation, in particular, are three‐dimensionally preserved and include remains of mineralized soft tissues, allowing for more rigorous reconstruction of musculoskeletal anatomy, either from well‐preserved skeletal elements with muscle attachment scars (Johanson, 2003; Long, 1995; Sanchez et al., 2013) or preserved soft tissues of the muscle bodies and internal organs themselves (Trinajstic et al., 2013; Trinajstic, Long, et al., 2022). Nevertheless, how the musculoskeletal morphology from the well‐known, yet morphologically unspecialized arthrodires of the Gogo Formation compares to more specialized latest Devonian (Famennian) forms like *Dunkleosteus* remains poorly understood. Thus, current cranial musculoskeletal reconstructions of *Dunkleosteus* are out of date given the current state of knowledge of arthrodire anatomy, and a re‐investigation is timely.

Recently, we re‐examined well‐preserved specimens of *Dunkleosteus terrelli* from the CMNH hypodigm as part of a project describing the morphology and gnathal histology of a new species of *Dunkleosteus* (*D. tuderensis*) from Russia (Lebedev et al., 2023). This, combined with data on preserved musculature from arthrodires from the Gogo Formation, enabled us to develop a new musculoskeletal reconstruction of *D. terrelli*, primarily focusing on the jaw muscles and the other parts of the skeleton involved in feeding. Our goal is for this new reconstruction to provide a framework for future biomechanical studies of this spectacular Devonian apex predator.

## 2 MATERIALS AND METHODS

This study is primarily based on specimens of *Dunkleosteus terrelli* housed at the CMNH, with additional observations drawn from *D. terrelli* material at the AMNH, Cincinnati Museum Center (CMC), and the Natural History Museum, London, UK (NHMUK). Previous studies have concluded that the *Dunkleosteus* material from the Cleveland Shale most likely represents a single species: *D. terrelli* (Hlavin, 1976). Special focus was paid to CMNH 6194, CMNH 7424, CMNH 6090, CMNH 7054, and CMNH 5768: a series of five near‐complete, three‐dimensionally mounted dermal skeletons from single individuals (listed from smallest to largest) ranging from juveniles to large adults. These specimens were originally preserved in a flattened state and were mounted via manual retro‐deformation in the 1920s; however, their morphology generally agrees with later specimens preserving some original curvature from the Cleveland Shale and uncrushed specimens from Morocco (Engelman, 2024). Morphological observations were made based on direct observations of *Dunkleosteus* material, 3D models of these specimens uploaded by the CMNH to MorphoSource (https://www.morphosource.org/organizations/000697777), or a 3D model of a cast of CMNH 5768 uploaded by the University of Michigan to UMORF (https://umorf.ummp.lsa.umich.edu/wp/specimen-data/?Model_ID=1336). 3D models used in these analyses can be accessed online or downloaded via these links.

Observations of other large Cleveland Shale arthrodires (*Gorgonichthys*, *Heintzichthys*, and *Bungartius*) were also made from direct examination of CMNH specimens, while observations of Gogo Formation arthrodires were based on specimens held at the Western Australian Museum (WAM) and the NHMUK. Additional observations were drawn from the previously published literature. Among arthrodires from the Gogo Formation, special attention is paid to *Eastmanosteus calliaspis*, which is an early‐diverging member of Dunkleosteoidea (Dennis‐Bryan, 1987; Zhu & Zhu, 2013; Zhu, Zhu, & Wang, 2016; but see Jobbins et al., 2025) and thus more closely related to *D. terrelli* than the other Gogo Formation arthrodires. Extensive comparisons were also made with the large macropredatory aspinothoracidans *Gorgonichthys clarki* (mainly CMNH 7129) and *Heintzichthys gouldii* (e.g., CMNH 5130, 5648). While these taxa are more distantly related to *Dunkleosteus* (Carr & Hlavin, 1995, 2010), they are known from well‐preserved cranio‐mandibular remains and converge with *Dunkleosteus* in their cranio‐mandibular morphology, likely due to similar macropredatory dietary habits (Denison, 1978; Dunkle & Bungart, 1946). As a result, comparisons with these taxa are useful to examine how convergent specializations for macropredation may have shaped the functional anatomy of large arthrodires.

**FIGURE 1:**
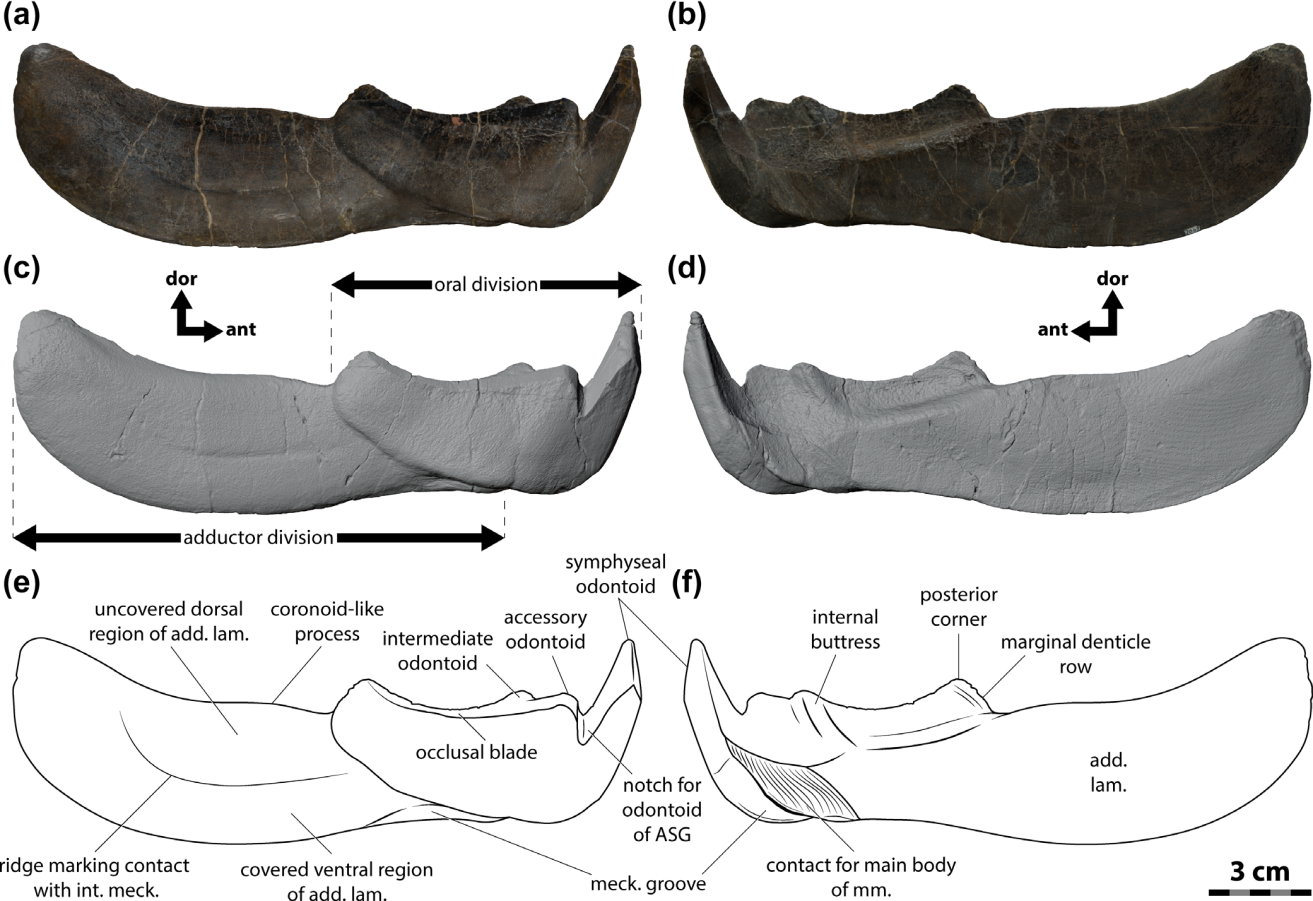
Photographs (a, b), surface models (c, d), and line drawings (e, f) of an infragnathal of *Dunkleosteus terrelli* (CMNH 7069) in external (a, c, e) and internal (b, d, f) views, showing features of relevance on this element for this study. Figure is largely modified from Lebedev et al. (2023), fig. 2). Note that in this specimen the marginal dentition and coronoid‐like process are not as developed as in other individuals (compare with Johanson & Smith, 2005, fig. 6 and Lebedev et al., 2023, fig. 2). Add. lam., adductor lamina; ant, anterior; ASG, anterior supragnathal; dor, dorsal; int. meck., intermediate, unossified portion of the Meckelian cartilage; meck. groove, Meckelian groove; mm, mentomandibular ossification.

General skeletal terminology for arthrodires in this paper follows Heintz (1932), Miles and Westoll (1968), Miles and White (1971), Denison (1978), and Miles and Dennis (1979). The term “cheek unit” refers to the structure formed by the suborbital, postsuborbital, and postnasal plates as well as the palatoquadrate cartilage and ethmoid ossification, following some prior authors (Miles, 1969; Miles & White, 1971), as these structures often form a functional unit. Infragnathal terminology and morphological information on the cartilages of the lower jaw (Figure 1) follow Lebedev et al. (2023). Specifically, “denticle” refers to small, superficial dentin structures that have pulp cavities and appear to be homologous with teeth (Johanson & Smith, 2005; Rücklin et al., 2012; Smith & Hall, 1990), whereas “odontoid” refers to tusk‐like projections formed by growth and wear of the bone. For musculature nomenclature and homologies, see Table 1.

**TABLE 1:** List of muscles examined in this study and their inferred insertions, origins, and functions.

Muscle	Origin	Insertion	Function
*m. adductor mandibulae*	Adductor fossa formed by ventral edge of palatoquadrate and internal face of suborbital plate	Adductor fossa of intermediate Meckel's cartilage	Mandible adduction
*m. levator palatoquadrati*	Anterior fossa on lateral consolidated region of skull roof	Dorsal surface of palatoquadrate just posterior to orbit	Unclear, stabilize mandibular arch relative to skull roof and neurocranium?
*m. preorbitalis*	Descending “lip” of ethmoid ossification and potentially fossa it forms with postnasal and internasal plate	Median raphe of m. adductor mandibulae	Mandible adduction, cranial depression
*m. coracomandibularis*	Fossa on internal face of mentomandibular ossification	Anterior border of interolateral plate on trunk armor	Mandible abduction, cranial depression
*m. levator capitis major*	Internal surface of mediodorsal	Thickened nuchal region of skull roof	Cranial elevation, mandible abduction (indirectly)
*m. levator capitis minor*	Lateral to levator capitis major on trunk armor	Lateral to levator capitis major on nuchal region of skull roof	Cranial elevation, mandible abduction (indirectly)
*m. cucullaris*	Dorsal edge of postbranchial lamina on anterior lateral plate	Cucullaris fossa on skull roof	Cranial depression, mandible adduction (indirectly)

Musculoskeletal reconstructions in paleontology typically involve the use of phylogenetic bracketing (Bryant & Russell, 1992; Witmer, 1995). Unfortunately, because arthrodires are stem‐gnathostomes, their basal extant phylogenetic bracket for soft tissues would be cyclostomes (hagfish, lamprey): specialized jawless vertebrates for which oral musculoskeletal homologies with gnathostomes remain contentious (see, e.g., Ziermann et al., 2014). This means many musculoskeletal inferences in arthrodires are type II inferences, based only on extant jawed vertebrates (sensu Witmer, 1995) unless otherwise noted. The preservation of muscle tissue in several Gogo Formation arthrodires (Trinajstic et al., 2007, 2013) provides a unique opportunity to directly observe the musculature of these extinct taxa (type I inferences) and more rigorously constrain the condition in *Dunkleosteus*, with the caveat being that sometimes these muscles are incomplete or only represented by their terminal attachments.

Eubrachythoracid phylogeny in this study broadly follows Carr and Hlavin (2010), Zhu and Zhu (2013), Zhu, Zhu, and Wang (2016), and Boyle and Ryan (2017). Most studies sampling a broad array of dunkleosteoids have recovered *Eastmanosteus calliaspis* as one of the earliest‐diverging dunkleosteoids, whereas *Dunkleosteus terrelli* is recovered as deeply nested among later‐diverging latest Devonian forms. We do note some studies have potentially not recovered these taxa in a monophyletic Dunkleosteoidea (Jobbins et al., 2022, 2024, 2025), but this may be a consequence of the more limited taxonomic sampling of these studies (e.g., not including taxa such as the basal aspinothoracidans *Bullerichthys*, *Bruntonichthys*, and *Kendrickichthys* that might be relevant for polarizing states at the base of Pachyosteomorpha), and/or their character matrices focused on phylogenetic relationships within Selenosteidae.

We use the term “macropredatory aspinothoracidans” to informally refer to the arthrodires *Dinichthys herzeri*, *Gorgonichthys clarki*, *Heintzichthys gouldii*, *Holdenius holdeni*, and possibly *Hadrosteus rapax*. These taxa show substantial morphological convergences with *Dunkleosteus* in jaw morphology, to the point they were originally regarded as closely related or even congeneric (see discussion in Carr, 1995; Carr & Hlavin, 2010; Denison, 1978; Dunkle & Bungart, 1946; Heintz, 1932). They also consistently occupy a similar place in most phylogenetic analyses, apical to the most basal aspinothoracidans like *Tapinosteus*, *Bullerichthys*, *Kendrickichthys*, and *Bruntonichthys* but basal to most or all of Selenosteidae (Boyle & Ryan, 2017; Carr, 1991; Fitzpatrick et al., 2024; Jobbins et al., 2022, 2024; Zhu & Zhu, 2013; Zhu, Zhu, & Wang, 2016). The monophyly of this group has not been recovered in all analyses, but this may be due to incomplete codings for *D. herzeri* (Carr & Hlavin, 2010) or no analysis considering all putative members of this group (*Holdenius* has never been included in a phylogenetic analysis). Furthermore, several characters identified in the present study, such as the development of symphyseal odontoids, reduced or absent symphyseal teeth, an expanded ethmoid‐anterior supragnathal contact with a large, anteroventrally descending lip (convergently developed in derived Dunkleosteoidea), a posterior facet distal to the odontoid on the anterior supragnathal (unique to macropredatory aspinothoracidans), and absence of an intermediate odontoid with internal buttress (differentiating their infragnathals from the superficially similar dunkleosteoids) have not been considered in prior phylogenetic analyses but may represent synapomorphies of this group. If this group is recovered as monophyletic by future studies, the name Dinichthyidae Newberry, 1885 would probably have priority (Carr & Hlavin, 1995). Nevertheless, in the absence of a formal phylogenetic analysis, we treat “macropredatory aspinothoracidans” as an informal grouping in order to more easily discuss patterns of functional morphology.

## 3 RESULTS

**FIGURE 2:**
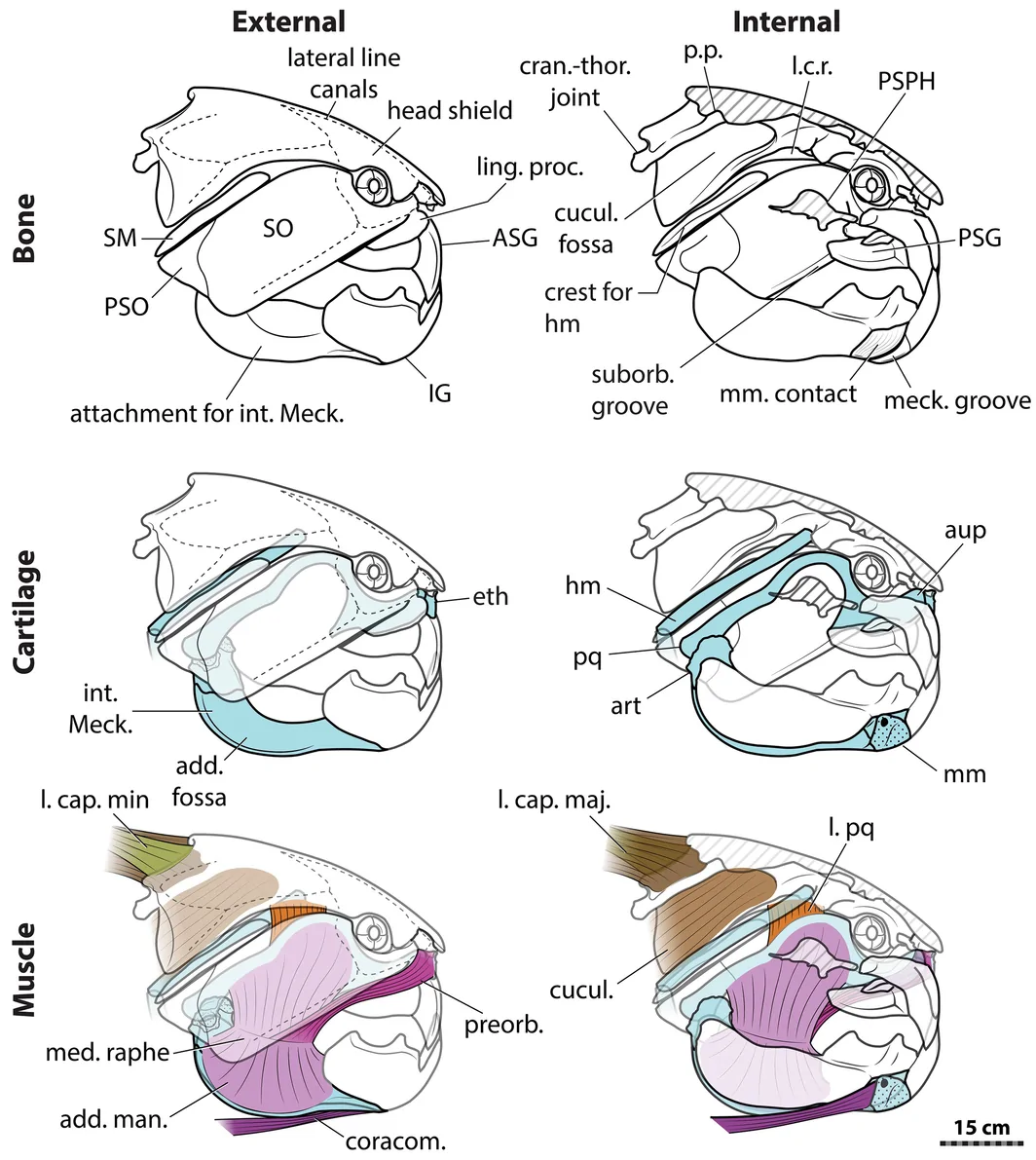
Bony (top), cartilaginous (middle), and muscular (bottom) anatomy of *Dunkleosteus terrelli* in external (left) and internal (right) views. Image here largely modeled after CMNH 7054, with missing elements replicated after other specimens. Mandible is slightly disarticulated from palatoquadrate to better show articulations between cranium and mandible. Lateral and medial fossae of the lateral consolidated region are unoccupied due to uncertainties regarding their function. Stippled areas represent articular surfaces. add. man., *m. adductor mandibulae*; art, articular cartilage; aup, autopalatine cartilage; coracom., *m. coracomandibularis*; cran.‐thor. joint, cranio‐thoracic joint; cucul., *m. cucullaris*; cucul. fossa, cucullaris/parabranchial fossa; eth, ethmoid ossification; hm, hyomandibula; IG, infragnathal; l. cap. major/minor; *m. levator capitis major/minor*; l.c.r., lateral consolidated region; l. pq, *m. levator palatoquadrati*; ling. proc., linguiform process of suborbital; med. raphe, median raphe; pq, palatoquadrate; preorb., preorbitalis; PSG, posterior supragnathal; PSO, postsuborbital; PSPH, parasphenoid; p.p, paired pits on skull roof; SM, submarginal; SO, suborbital; suborb. groove, suborbital groove. Other abbreviations follow Figure 1.

The general anatomy of the cranial plates in *Dunkleosteus* has been the subject of several studies (Branson, 1908; Dunkle & Bungart, 1942a, 1946; Heintz, 1932; Lebedev et al., 2023; Newberry, 1875, and references therein), and we refer readers to those papers for further details. Similarly, for the dental ontogeny and general histology of the gnathal plates in *Dunkleosteus*, we refer readers to Johanson and Smith (2005) and Lebedev et al. (2023), respectively. Instead, the present study focuses on aspects of the anatomy of *Dunkleosteus* that have received less focus, specifically the jaw cartilages, muscle attachments, and overall functional anatomy (Figure 2), as well as osteological correlates on the cranial plates not considered by prior studies. The present manuscript is organized in terms of putative functional groups, specifically the following:

1. The *m. adductor mandibulae* and other skeletal elements related to its attachments and function, including the articulation between the upper and lower jaws, the palatoquadrate attaching to the internal face of the cheek unit, and the intermediate Meckelian cartilage associated with the lower jaw.
2. The suborbital plate, in particular the large suborbital groove formed by its ventral surface, the linguiform process, and the ethmoid ossification of braincase, as well as the *m. preorbitalis* that occupied this region.
3. The lateral consolidated region on the internal surface of the headshield, which contains the origins for the *m. levator palatoquadrati* and probably the *m. levator hyomandibulae*.
4. The submarginal plate, which attaches to and overlies the hyomandibula (Carr et al., 2009; Gardiner & Miles, 1990; Hu et al., 2017; Miles & White, 1971).
5. Muscles spanning the cranio‐thoracic joint (*m. cucullaris*, *m. levator capitis major*, and *m. levator capitis minor*), which in arthrodires play a role in raising and lowering the head (Anderson & Westneat, 2007, 2009; Heintz, 1932; Miles, 1969; Trinajstic et al., 2013).
6. The mandibular symphysis, which includes the origin of the *m. coracomandibularis*, but also represents an important cranio‐mandibular articulation and has previously been suggested to be a location of kinesis in arthrodires (Heintz, 1932; Hussakof, 1906; Vézina, 1986, but see Dean, 1901, Miles, 1969)

These functional groups each center around major muscles, soft tissues, or cranio‐mandibular joints and their attachment sites on the bony or cartilaginous skeleton, analogous to how morphofunctional anatomical descriptions are structured in other studies (e.g., Holliday, 2009; Wilga, 2005). Questions of suction feeding, gape, and evolutionary trends among arthrodires involve elements from multiple functional groups working together, and so are considered further under Section 4. In cases where an element may fall under multiple functional groups (e.g., the posterior region of the palatoquadrate serving as the origin for the *m. adductor mandibulae* and the anterior autopalatine ossification of this cartilage serving as the attachment for the supragnathals), we discuss the relevant features in the context of their overlying soft tissues.

### 3.1 Functional group 1: Adductor mandibulae and associated structures

#### 3.1.1 Palatoquadrate

**FIGURE 3:**
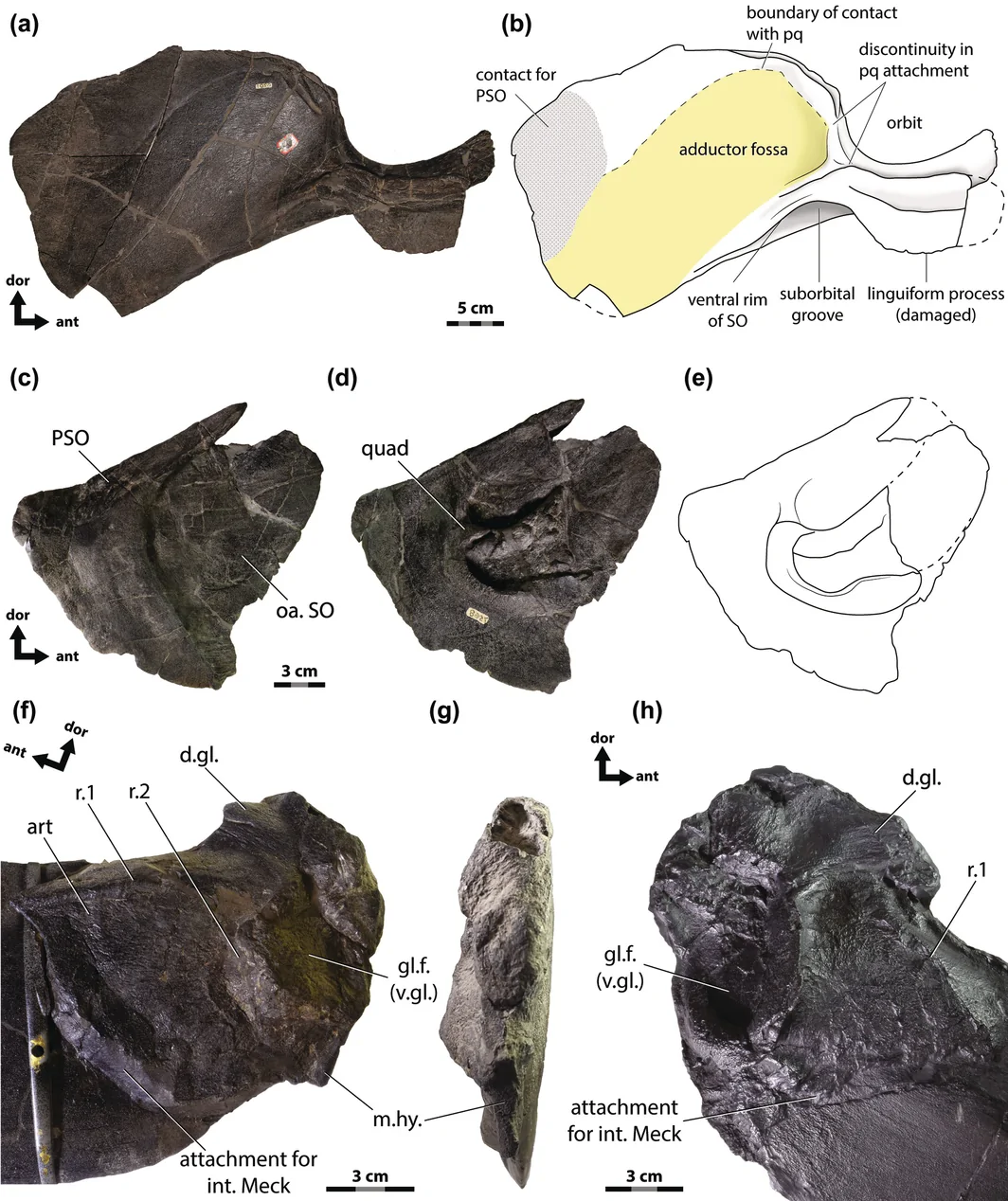
(a, b) Photograph (a) and interpretive drawing (b) of the suborbital of *Dunkleosteus terrelli* (CMNH 6201, reversed) in internal view, showing the sinusoidal boundary between the suborbital plate and palatoquadrate. (c, e) Photographs (c–d) and line drawing (e) of the posterior right cheek of *D. terrelli* (CMNH 7568) in external (c) and internal (d–e, reversed) views, showing the location of the perichondrally ossified quadrate articulation of the palatoquadrate. (f, g) Articular cartilage of *D. terrelli* (CMNH 7054) in left lateral (f) and posterior (g) views. (h) Articular cartilage of a second specimen of *D. terrelli* (CMNH 5768) in lateral view. Abbreviations follow Figure 2 with the addition of d.gl, dorsal glenoid facet; gl.f., glenoid fossa; m.hy., attachment for mandibulohyoid ligament; oa.SO, overlap area for suborbital plate on postsuborbital plate; r.1, ridge possibly marking dorsal border of (part of) the adductor fossa; r.2, ridge marking ventral border of glenoid fossa; quad, quadrate articulation on palatoquadrate; v.gl., ventral glenoid facet.

The palatoquadrate of arthrodires has a characteristic “omega‐shape” (Brazeau et al., 2023; Gardiner & Miles, 1990; Hu et al., 2017; Schaeffer, 1975), with its posterior portion forming a well‐defined, medially oriented adductor fossa that spans most of the cheek unit. The palatoquadrate surrounds the orbit posteriorly and ventrally at its anterior end, whereas its posterior end is associated with the postsuborbital plate, where it forms a prominent articulation (the quadrate articulation) with the lower jaw. *Dunkleosteus* does not differ in this respect. Many specimens preserve a distinct visceral thickening near the ossification center of the postsuborbital plate near the jaw articulation (as in most arthrodires; Gardiner & Miles, 1990; Miles & Westoll, 1968). In at least one specimen (CMNH 6201), the contact between the palatoquadrate and suborbital is readily visible on the suborbital in internal view (Figure 3a,b). The contact between the palatoquadrate and cheek unit is not continuous, with a slight discontinuity near the posterior orbit separating its anterior contact on the linguiform process from the adductor fossa on the postorbital region of the cheek. The contact for the overlap of the postsuborbital plate can also be recognized (Figure 3b).

**FIGURE 4:**
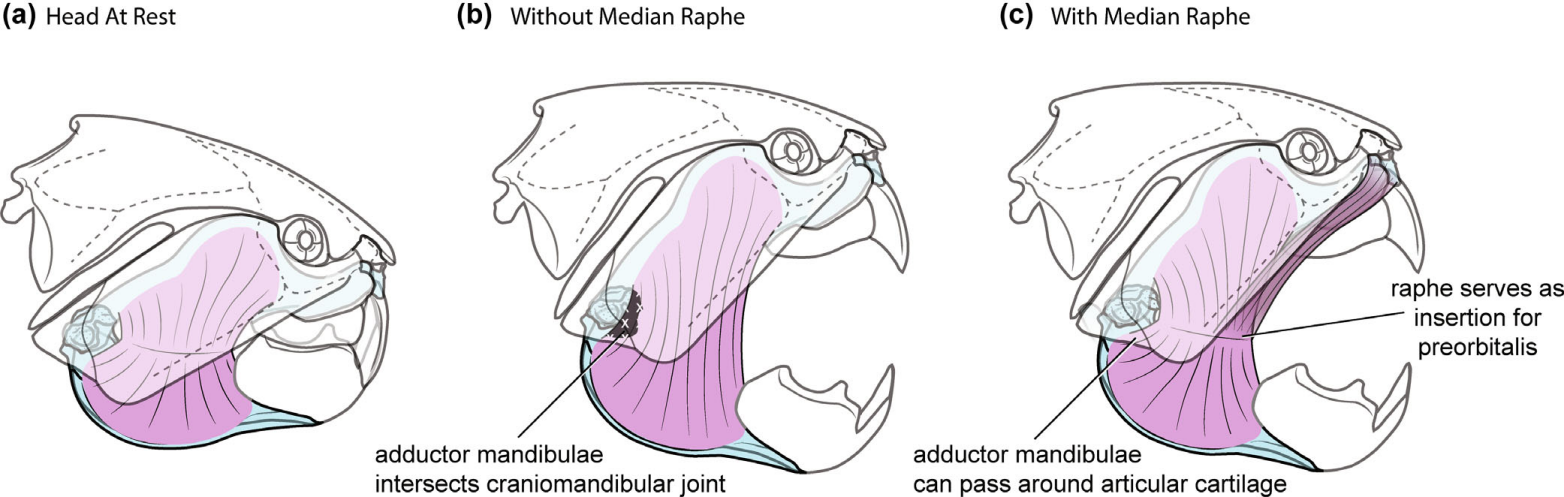
Skull of *Dunkleosteus terrelli* (modeled after CMNH 7054) with jaws closed (a) and open (b, c) showing how the presence of a median raphe affects the kinematics of the jaw. The highlighted area in (b) represents the region of intersection between the *m. adductor mandibulae* and the articular cartilage when the mouth is opened if there is no median raphe.

However, the adductor fossa of *Dunkleosteus* also shows several unique features compared to other arthrodires. The adductor fossa is anteroposteriorly elongate and asymmetrical, with its deepest point located just posterior to the orbit (Figure 3a; File S1B) and its longest axis oriented anterodorsally–posteroventrally (about 41° to the anteroposterior axis). By contrast, in other arthrodires the adductor fossa is a simple semi‐circle, deepest at its anteroposterior midpoint (File S1A; see also Dennis & Miles, 1979b, fig. 16; Gardiner & Miles, 1990, figs. 8 and 21; Hu et al., 2017, fig. 3, supplementary fig. 4a; Miles & White, 1971, figs. 23, 24, and 33). These points, along with the development of the linguiform process and ventral rim of the suborbital plate at the level of the anterior end of the adductor fossa restricting the anteriormost fibers of the *m. adductor mandibulae* from extending directly ventral, suggest the longest fibers of the dorsal head of the *m. adductor mandibulae* in *Dunkleosteus* were oriented at a relatively oblique angle to the contractile path of this muscle. This contrasts with other arthrodires, where the more semicircular shape of the palatoquadrate allows for a more verticalized contractile path and there is no significant blockage of the anterior fibers (Gardiner & Miles, 1990; Hu et al., 2017). CMNH 6201 is a very young individual of *D. terrelli*; given this taxon shows a progressive elongation of the postorbital cheek throughout ontogeny (see Heintz, 1932 and below), this condition would likely be exaggerated in adults. Because of the shape of the adductor fossa, its longest axis is oriented increasingly perpendicular to the anteroposterior axis of the mouth as the jaw opens, increasing the mechanical advantage of the anterior fibers of the *m. adductor mandibulae* (Figure 4).

#### 3.1.2 Jaw articulation

The jaw articulation of *Dunkleosteus* is formed by the palatoquadrate (on the upper jaw) and articular cartilage (on the lower). The cranial position of this joint can be identified by the large thickening for the reception of the quadrate on the internal face of the postsuborbital plate (Figure 3d: quad). The quadrate articulation of *Dunkleosteus* is located substantially more posteriorly on the head compared to other arthrodires, almost at the level of the cranio‐thoracic joint (see Overall size of the cheek unit and mandibular arch).

The articular cartilage is located at the posterodorsal margin of the mandible (Figure 3f–h) and forms the mandibular portion of the jaw articulation of *Dunkleosteus*. There are two distinct articular facets: a dorsal one and a (ventro)lateral one. This configuration is observed in multiple specimens of *D. terrelli* (CMNH 5768, Figure 3f,g; CMNH 7054, Figure 3h). The dorsal articular facet may be absent in other specimens (e.g., CMNH 7129), but this appears to be due to taphonomic damage to the specimen rather than intraspecific variability. The two articular facets observed here appear to represent the same structures seen on the mandibles of other arthrodires (see Johanson, 2003).

The more ventral articular facet appears to correlate with the lateral glenoid, and thus represents the main articulation point for the quadrate. When the mandibles are in life orientation, this structure faces almost directly lateral (Figure 3f,h). The articular facet itself has a simple circular shape, with a thicker anteroventral lip. In CMNH 7054, the articular facet appears less flattened, with a robustly rimmed glenoid fossa directed more posterolaterally (Figure 3f: r.2, gl.f). By contrast, in other arthrodires, including buchanosteids (Hu et al., 2017) and most of the Gogo forms, the lateral glenoid has a more posterolateral orientation.

The dorsal articular facet of *D. terrelli* is located anterior and slightly medial to the lateral glenoid, with the articular surface being slightly subdivided. Whereas the ventral articulation connects to the main condyle of the palatoquadrate, the dorsal articulation is thought to form a secondary joint with the detent process of the palatoquadrate (Stensiö, 1963, p. 236). It should be noted, however, that some authors have argued the dorsal articulation could not have contacted the palatoquadrate and instead proposed it articulated with the hyomandibula or another epihyal element (Gardiner & Miles, 1990, p. 196; Hu et al., 2017, p. 10). No detent process is observable on the available *Dunkleosteus* palatoquadrates; the corresponding region appears to have been poorly ossified. Additionally, more ventrally, a large elongate surface is visible on the articular cartilage, representing a large mandibulohyoid facet for a ligament running from the back of the mandible to attach to the ceratohyal; as the ceratohyal is rotated posteriorly, the mandibulohyoid ligament depresses the mandible.

**FIGURE 5:**
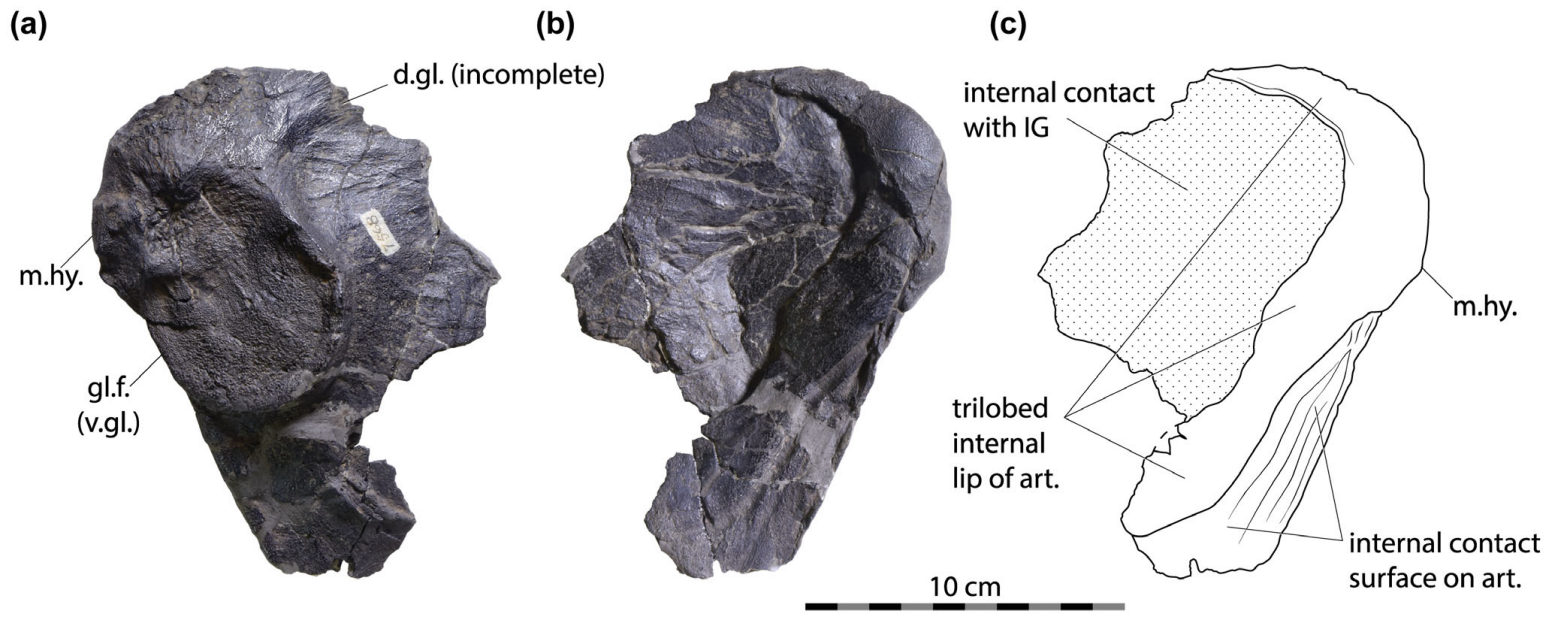
Photographs (a–b) and line drawing (c) of the right articular cartilage of *Dunkleosteus terrelli* (CMNH 7658) in external (a) and internal (b–c) views, showing the trilobate, internally overhanging lip of the articular cartilage around the posterior edge of the infragnathal and the internal contact face (for the ceratohyal?). d.gl, dorsal glenoid facet; IG, infragnathal; m.hy., attachment for mandibulohyoid ligament.

In one specimen of *Dunkleosteus* (CMNH 7568, Figure 5), the articular shows a trilobed lip in internal view that would have wrapped around the posterodorsal edge of the infragnathal and covered it in internal view. A similar arrangement is observed in some other arthrodire articulars (Western Australian Museum, Perth, Australia [WAM] 86.9.683, *Incisoscutum ritchei*; Johanson, 2003, fig. 4g), though in these forms the lobes extend across a greater proportion of the infragnathal due to the proportionally larger size of the articular cartilage. Ventral to this, the articular in CMNH 7568 displays a prominent contact surface for another structure, with an overall roughened texture and well‐developed ridges giving it a corrugated appearance. Once again, a similar structure appears to be present in at least some other eubrachythoracid arthrodires (WAM 86.9.683, *I. ritchei*; Johanson, 2003, fig. 4g), though perhaps not as well‐developed. Exactly what is attached here is unclear. On the one hand, this could represent another contact with the intermediate Meckelian cartilage. On the other hand, this could represent a contact or articulation face between the mandible and the ceratohyal. This is potentially supported by the articulation for the mandibulohyoid ligament being located roughly along the same line as the long axis for this contact face in both *Incisoscutum* and *Dunkleosteus*. Direct articulation between the mandibular arch and ceratohyal is rare in fishes, but has been reported in ptyctodonts (Forey & Gardiner, 1986; Long, 1997; Trinajstic et al., 2012). Nevertheless, such a relationship does not appear to be present in buchanosteids (Hu et al., 2017) or phyllolepids (Carr et al., 2009), though the two elements are closely associated in the latter. Such a contact between the ceratohyal and mandible—if it existed—might be a way of stabilizing the mandibular arch posteriorly given its large size and otherwise seemingly limited posterior stabilization in *Dunkleosteus* (see below). However, the ceratohyal is poorly known in eubrachythoracid arthrodires, and thus whether its morphology supports this conclusion cannot be evaluated.

In many arthrodires, anterior to the contact with the articular on the internal face of the infragnathal, there is a distinct groove running between the end of the contact with the articular and the base of the Meckelian groove (WAM 86.9.683, *I. ritchei*; Johanson, 2003, fig. 4g), roughly where the bulk of the Meckelian cartilage passes from the external to the internal face of the infragnathal (Lebedev et al., 2023). This groove has often been regarded as a groove for the ramus mandibularis internus VII nerve (Gardiner & Miles, 1990; Miles & Dennis, 1979; Miles & Westoll, 1968). However, the fact that this groove runs between the Meckelian groove and the articular cartilage, as well as the fact that the bone exhibits a smoother texture and is seemingly inset ventral to this groove, raises the question of whether it might actually mark an internal contact face with the intermediate Meckelian cartilage on the internal face of the mandible. This is supported by the groove for this nerve in buchanosteids being significantly higher on the mandible and located entirely on the Meckel's cartilage (Hu et al., 2017). A similar contact face may be present in *Dunkleosteus*, but is relatively low and poorly defined in most specimens. This suggests the contact with the intermediate Meckelian cartilage may have been less extensive internally.

#### 3.1.3 M. adductor mandibulae

The main jaw‐closing muscle in lower vertebrates is the *m. adductor mandibulae*. The biomechanical origin of this muscle in eubrachythoracid arthrodires has been debated by several authors. Heintz (1932, pp. 190–191) considered the *m. adductor mandibulae* (= “*m. levator gnathalis*” of that study) to originate within the suborbital groove (see “Suborbital groove and *m. preorbitalis*” below), the relative narrowness of which is one reason he suggested the jaw adductors of *Dunkleosteus* were relatively weak and primarily served to stabilize the more powerful actions of the muscles spanning the cranio‐thoracic joint (= *m. cucullaris*, see below). Historically, many authors considered most if not all of the *m. adductor mandibulae* to originate on the lateral consolidated region of the internal headshield, including Dunkle and Bungart (1943, p. 81, 1946), Stensiö (1963, p. 376), Miles (1969), and Anderson and Westneat (2007, 2009). However, other studies preferred an arrangement where some or all of the *m. adductor mandibulae* attached to the palatoquadrate (Long, 1995; Miles, 1967; Miles & Westoll, 1968; Vézina, 1988). Long (1995) addressed the disparity in these two interpretations the most directly, suggesting that the *m. adductor mandibulae* almost exclusively originated on the palatoquadrate, within the adductor fossa, noting that the presence of a palatoquadrate bordering the adductor chamber dorsally along the dorsal margin of the cheek unit would have separated the adductor chamber and the skull roof and prevented the *m. adductor mandibulae* from extending to the lateral consolidated region. Datovo and Johnson (2025) further proposed the dorsal head of the adductor mandibulae in arthrodires was divided into a *pars buccalis* and *pars facialis* separated by the trigeminal nerve, resembling the arrangement seen in living gnathostomes. This was based on their interpretation of the morphology in buchanosteids (Hu et al., 2017), but if and where this division was present in *Dunkleosteus* cannot be evaluated as the foramen for the trigeminal nerve appears to have been located on the unmineralized middle region of the palatoquadrate.

Most, if not all, of the *m. adductor mandibulae* in *Dunkleosteus* appears to have inserted on the intermediate portion of the Meckelian cartilage of the lower jaw (Figure 2). Unlike the anterior (mentomandibular ossification) and posterior (articular) parts of the Meckel's cartilage, the intermediate portion is unossified in arthrodires (Miles & Westoll, 1968), and previous studies have considered it absent in *Dunkleosteus* (Anderson & Westneat, 2009; Heintz, 1932; Hussakof, 1906; Young, 2010). However, several features suggest this cartilage was present in *D. terrelli* and help constrain its potential morphology. Contact faces for the intermediate Meckelian cartilage are present on the mentomandibular ossification and articular cartilage (Figure 3f–h, File S2), defining its anterior and posterior boundaries. A Meckelian groove is present on the ventral surface of the oral region of the infragnathal, marking the location where the intermediate cartilage passed underneath the infragnathal from its lateral to its medial face (Lebedev et al., 2023; see also Figure 1, File S2, this study). And finally, the ventral half of the external face of the adductor lamina of the infragnathal shows a broad area of contact for the intermediate Meckelian cartilage, marked by a slightly inset region with a smoother bone texture suggestive of an overlying cartilaginous contact (Lebedev et al., 2023, fig. 4; see also Figure 1, File S2, this study). This region is separated from the dorsal half of the adductor lamina, which apparently was not covered by cartilage based on its more rugose bone texture, by a distinct crescent‐shaped groove marking the dorsal boundary of the infragnathal–Meckelian cartilage contact. This groove is largely continuous with the anterior border of the articular cartilage (File S2), further suggesting it represents a landmark for the borders of the intermediate Meckelian cartilage rather than a path for a nerve or blood vessel. Based on the extent of these features, at least half of the external face of the adductor lamina was covered by the intermediate Meckelian cartilage in life.

**FIGURE 6:**
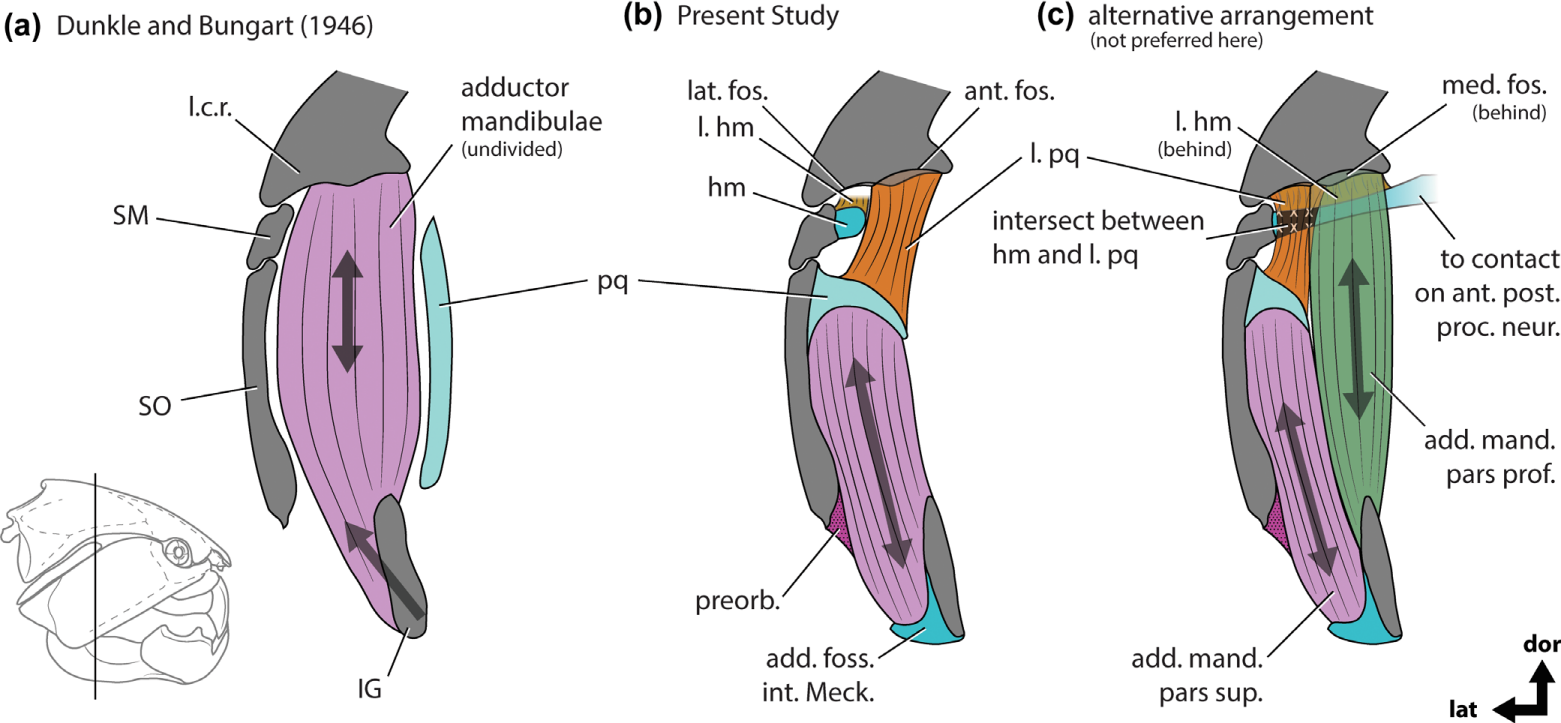
Muscle reconstruction of *Dunkleosteus terrelli* in transverse view, comparing the reconstruction in Dunkle and Bungart (1946) (a, redrawn and modified from that study) to that favored in the present analysis (b), and an alternative arrangement where the *m. adductor mandibulae* is divided into multiple branches with a deep branch originating on the lateral consolidated region (c). Note that (b, c) allow for straighter contractile paths of the branches of the *m. adductor mandibulae*. Abbreviations follow Figures 1 and 2 with the addition of add. mand. pars prof., deep branch of the *m. adductor mandibulae* (*pars profundus*); add. mand. pars sup., superficial branch of the *m. adductor mandibulae* (*pars superficialis*); ant.fos., anterior fossa (of lateral consolidated region); ant. post. proc. neur., attachment for anterior postorbital process of neurocranium; lat, lateral; lat.fos., lateral fossa; l. hm., *m. levator hyomandibulae*; med.fos., medial fossa.

In chondrichthyans (Motta & Wilga, 1995, 1999) and buchanosteid arthrodires (Hu et al., 2017; Young et al., 2001), the lateral surface of the Meckelian cartilage forms a well‐developed adductor fossa. This allows the *m. adductor mandibulae* to contract in a straight path between the upper and lower jaws (Ferrara et al., 2011). The lateral face of the infragnathal in *Dunkleosteus* is slightly convex (Figure 6b,c), suggesting the adductor fossa was formed almost entirely by the intermediate Meckelian cartilage. This is supported by a ridge on the articular cartilage roughly following the dorsal infragnathal–Meckelian cartilage contact (Figure 3f, r.1.), which, based on comparisons with buchanosteids (Hu et al., 2017, fig. 2b), may represent (part of) the dorsal border of the adductor fossa. If there was no adductor fossa, the *m. adductor mandibulae* would have to insert directly onto the flat‐to‐convex surface of the infragnathal. This would require the fibers of the *m. adductor mandibulae* to pull at oblique angles near their insertion to close the jaw, greatly reducing jaw efficiency. For example, in Dunkle and Bungart (1946, fig. 4e,f), the intermediate Meckelian cartilage is not restored, and the fibers of *m. adductor mandibulae* are required to pull at oblique angles (Figure 6a), whereas, if an adductor fossa was present, the insertion of the muscles is shifted laterally so that the muscles are now capable of contracting along a straight line (Figure 6b,c).

Although most of the *m. adductor mandibulae* inserted on the intermediate Meckelian cartilage, at least part of it inserted onto the dorsal portion of the infragnathal not covered by cartilage. The infragnathal of *D. terrelli* has a distinct eminence at the dorsal side of the adductor lamina resembling the coronoid process in other vertebrates (Lebedev et al., 2023). This process is most strongly pronounced in juvenile individuals, but is poorly defined in adults. Examination of adult individuals suggests this structure is still present but has been obscured through hyperossification and remodeling of the surrounding bone throughout ontogeny. Most of the dorsal surface of the infragnathal does not show strong indications of attachments for muscles, but there is a distinct patch of irregular surface texture near this eminence in multiple infragnathals (e.g., CMNH 6194 and CMNH 7069) that suggests a soft tissue structure, most likely a muscle, attached here. Similarly, traces of muscle attaching to the adductor lamina of the infragnathal are known from the pachyosteomorph taxon *Eastmanosteus calliaspis* (Trinajstic et al., 2007; Trinajstic, Briggs, & Long, 2022) and *Incisoscutum ritchiei* (Trinajstic, Long, et al., 2022, supplementary fig. S1). A bony attachment for the *m. adductor mandibulae* on the dorsal half of the adductor lamina in *Dunkleosteus* would be expected to allow the medial fibers of this muscle to apply much higher forces because of the higher pullout strength of a bony enthesis relative to a cartilaginous attachment (Huber et al., 2009; Liem & Summers, 1999). This might potentially allow higher bite forces in *Dunkleosteus* and other eubrachythoracids compared to taxa in which the entire origin of the *m. adductor mandibulae* is cartilaginous, such as chondrichthyans and non‐eubrachythoracid arthrodires (Ferrara et al., 2011; Miles & White, 1971; Wilga et al., 2007; Young et al., 2001). However, we have been unable to identify any bony origin for the *m. adductor mandibulae* on the cranium of *Dunkleosteus* and other eubrachythoracids (see Lateral consolidated region and *m. levator palatoquadrati*, below). This would make the limiting factor in such an arrangement the cartilaginous origin for these fibers of the *m. adductor mandibulae*, and thus the bony insertion would not result in higher bite force.

#### 3.1.4 Overall size of the cheek unit and mandibular arch

The cheek unit and mandibular arch of *Dunkleosteus* are elongate relative to total head length when compared to other arthrodires, making the mouth comparatively large. In more generalized eubrachythoracids (e.g., coccosteomorphs), the suborbital plate is relatively short, resulting in a small adductor fossa and a cheek unit much shorter than the head (Figure 8, Files S3–S5; Dennis‐Bryan, 1987, Gardiner & Miles, 1990, fig. 21). The length of the cheek, if only considering elements associated with the mandibular arch (suborbital and postsuborbital plates), is proportionally even smaller in generalized non‐eubrachythoracids (File S4), though in these forms the submarginal plate is large and plate‐like (Young, 2010). However, at least some of the smaller cheek in generalized non‐eubrachythoracids may be due to the smaller orbits in these forms, rather than a smaller adductor region (File S4). The cheek unit is somewhat larger relative to head size in pachyosteomorphs (average cheek length is 63.9% head length in coccosteomorphs, 80.7% in pachyosteomorphs; group membership in Coccosteomorpha versus Pachyosteomorpha is significant if added as an additional variable in an allometric scaling relationship, *t* = 5.614, *p* < 0.001). However, in many of these forms, such as selenosteids, the adductor chamber remains small due to the large orbits (File S5A).

By contrast, in *Dunkleosteus* the cheek unit is comparatively elongate, with the posterior margin of the postsuborbital plate reaching the level of the cranio–thoracic joint (Figure 8a,b). The orbits are comparatively small, resulting in most of the space between the posterior cheek and the adductor chamber being occupied by the adductor chamber. This contrasts with many aspinothoracidans, which also have a posteriorly extensive cheek but a smaller adductor region because of their large orbits (File S4). The mouth of *D. terrelli* is proportionally longer (but not wider) than in other arthrodires relative to either head length or estimated total length (TL; Engelman, 2023a, 2023b), including *Eastmanosteus calliaspis* and *Heintzichthys gouldii*, and became more exaggerated across ontogeny due to positive allometry of cheek unit and infragnathal length (Boyle et al., 2016; Engelman, 2023a; Heintz, 1932). This appears to be a true change in proportions rather than driven by choice of scaling proxy because the cheek unit also changes shape with growth, becoming progressively more elongate in larger individuals (Engelman, 2024, fig. 5; Heintz, 1932, p. 141). Examination of *Dunkleosteus* specimens suggests this change in proportions is almost entirely driven by elongation of the postorbital region of the suborbital plate (Engelman, 2024, fig. 5; Heintz, 1932, p. 141)—that is, the region of the adductor fossa—and thus reflects an enlargement of the adductor musculature relative to head or body size across ontogeny.

*Eastmanosteus calliaspis* displays an intermediate state: while the size of the cheek unit is somewhat more coccosteomorph‐like and still substantially shorter than the head shield, Dennis‐Bryan (1987, p. 3, 21) notes that among coeval Gogo Formation arthrodires this taxon has a comparatively elongate postorbital cheek (Files S3C and S4). For reference, while the postorbital division of the cheek represents ~65.7% of total cheek unit length in *E. calliaspis* (Dennis‐Bryan, 1987, p. 21), this same proportion is 72.7% in juvenile *Dunkleosteus terrelli* (CMNH 7424), 78%–80% in subadults/small adults (CMNH 6090 and CMNH 7054), and 83.1% in large adults (CMNH 5768) (File S4). The one caveat is that cheek proportions for *E. calliaspis* are only available for individuals about half maximum size (Dennis‐Bryan, 1987), and thus this proportion may have been slightly higher in larger individuals. Scaling the postorbital length of the cheek by head length seemingly standardizes this value and removes confounding variation due to allometry of the orbit; under this metric the length of the postorbital cheek in *Eastmanosteus* is similar to other arthrodires (i.e., similar to *Coccosteus* and *Plourdosteus*, smaller than *Hadrosteus*, but larger than in *Heintzichthys* or *Torosteus*; File S4). However, the postorbital cheek of *Dunkleosteus* still stands out as unusually long and positively allometric, only comparable to *Titanichthys* in proportional size (File S4). The cheek unit of *Dunkleosteus* is relatively elongate among arthrodires regardless of whether this is measured as total cheek unit length as a proportion of head size, postorbital cheek unit length as a proportion of head size, or postorbital cheek (File S5).

Cheek unit dimensions are positively allometric in arthrodires, with the allometric slope of total cheek length relative to head length being 1.10 ± 0.02, postorbital cheek length relative to head length being 1.23 ± 0.04, and postorbital cheek length relative to total cheek length being 1.11 ± 0.03 (File S6). Positive ontogenetic allometry within *Dunkleosteus terrelli* is more extreme than interspecific allometry across arthrodires: the allometric slope for total cheek length versus head length is 1.19 ± 0.04, for postorbital cheek versus head length is 1.43 ± 0.05, and for postorbital cheek length versus total cheek length it is 1.20 ± 0.05 (green lines in File S6). In both cases, scaling relationships between the postorbital cheek and head size (approximately overall body size) show the strongest allometry. If membership in Coccosteomorpha versus Pachyosteomorpha is added as an additional variable, membership is significant (*t* = 5.614, *p* < 0.001) but total cheek length scales close to isometry (1.03 ± 0.02). Positive allometry remains for the postorbital cheek if scaled versus head length (1.22 ± 0.05) or total cheek length (1.19 ± 0.03); group membership is not significant in the former (*t* = 1.98, *p* = 0.844) but is in the latter (*t* = −3.971, *p* < 0.001). As mentioned above for *D. terrelli*, most of these patterns appear driven by elongation of the postorbital region of the suborbital plate, roughly corresponding to the size of the adductor fossa on the palatoquadrate (Dennis & Miles, 1979b; Gardiner & Miles, 1990), and thus represents an allometric enlargement of the adductor musculature within Eubrachythoraci.

This positive allometry in cheek dimensions (especially the postorbital cheek) is better explained by functional adaptation rather than developmental constraints. Cheek scaling patterns observed here are not independent of lifestyle but are closely correlated with reconstructed dietary habits. The smallest taxa (*Millerosteus*, camuropiscids) have relatively gracile mouthparts and are thought to have fed on small prey, slightly larger taxa (*Coccosteus*, *Compagopiscis*, and *Incisoscutum*) are regarded as generalist predators, larger arthrodires (*Eastmanosteus*, *Hadrosteus*, *Plourdosteus*, and *Torosteus*) have been interpreted as small macropredators or “facultative piscivores” analogous to reef sharks, and most of the very largest arthrodires (*Dunkleosteus*, *Gorgonichthys*) are regarded as specialized predators of large vertebrates (Anderson, 2008b, 2010; Anderson & Westneat, 2009; Desmond, 1974; Dunkle & Bungart, 1946; Miles, 1969; Trewin, 1986; Trinajstic, Briggs, & Long, 2022). The one exception to this pattern—*Titanichthys*—would also be expected to show a large mouth/mandibular arch relative to body size due to its inferred planktivorous habits (Coatham et al., 2020). Notably, in *Titanichthys* while the cheek is relatively long, the postorbital region of the suborbital plate is short (comparable to a juvenile *Dunkleosteus*‐like CMNH 7424) and the great length of the cheek unit is driven by an autapomorphic posterior extension of the postsuborbital plate (Boyle & Ryan, 2017, figs. 3.1 and 8). Thus, while the mouth and mandibular arch of *Titanichthys* are long, the adductor fossa is not particularly large.

This pattern broadly resembles ecological and biomechanical patterns in other vertebrates. A somewhat similar correlation between relative prey size and body size is broadly seen in extant elasmobranchs (compare orectolobiforms versus small carcharhinids versus large carcharhinids and lamnids), but has not been quantitatively examined (J. Gayford and P. Sternes, personal communication, 2024). Increasing relative prey size with body size would be expected from an energetic perspective, as larger organisms often feed on relatively larger prey to meet their caloric needs (Carbone et al., 1999). By contrast, positive allometry of the adductor chamber would not be expected in a vacuum based on biomechanical considerations without an increase in relative prey size being involved. Assuming a conserved prey size and cranial bauplan, the adductor muscles of vertebrates are expected to scale isometrically or even show negative allometry, as absolute bite force is generally positively allometric but mechanical properties of food do not change with size (Mitchell et al., 2024). The relative size of the adductor muscles would not be expected to increase with body size unless larger species were feeding on proportionally larger and/or more resistant prey items (Mitchell et al., 2024, pp. 510–511). Thus, postorbital cheek length (= adductor chamber size) in arthrodires appears to be correlated with relative prey size, which is in turn correlated with body size, rather than postorbital cheek length and body size showing a direct, allometrically driven covariance.

The larger, more elongate mandibular arch of *Dunkleosteus* compared to other eubrachythoracid arthrodires appears to be the result of specializations for macropredation. Similar patterns of variation occur in elasmobranchs, where specialized macropredatory forms (e.g., Carcharhinidae) have proportionally larger mandibular arches than more generalized taxa (e.g., Squalidae) (Moss, 1972, p. 433). In non‐macropredatory sharks (e.g., *Chiloscyllium*, *Ginglymostoma*, *Heterodontus*, *Scyliorhinus*, and *Squalus*), the posterior end of the mandibular arch is located at or anterior to the posterior margin of the chondrocranium (De Iuliis & Pulerà, 2011; Dearden et al., 2021; Motta & Wilga, 1999; Staggl et al., 2022; Summers et al., 2004), whereas in many large, macropredatory species like lamnids (Mollen et al., 2012, figs. 2–6; Wilga, 2005), odontaspids (Winkel et al., 2024), sphyrnids (Mara et al., 2015, fig. 4), and large carcharhinids (McQuiston et al., 2017, fig. 3d; Moss, 1977), the mandibular arch extends significantly posterior to the braincase. Some smaller piscivores such as *Carcharhinus melanopterus* (former collections of the Zoölogisch Museum Amsterdam in the Naturalis Biodiversity Center, Leiden, the Netherlands [ZMA.PISC.]108682, ZMA.PISC.111010), *Rhizoprionodon terranovae* (ZMA.PISC.111362), *Prionace glauca* (former collections of the Rijksmuseum van Natuurlijke Historiche in the Naturalis Biodiversity Center, Leiden, the Netherlands [RMNH.PISC].34404), and *Triaenodon obesus* (ZMA.PISC.108703, ZMA.PISC.110760) seem to show an intermediate state; the mandibular arch extends slightly posterior to the cranium but not to the same extent as in larger sharks (Kamminga et al., 2017). The similarity of this pattern is striking, as in both large, macropredatory sharks and *Dunkleosteus* the enlargement of the mandibular arch appears to reflect a posterior extension of this structure relative to conserved landmarks like cranial nerve outlets (Carr et al., 2009).

However, despite the mandibular arch of *Dunkleosteus* being relatively large among eubrachythoracid arthrodires, it is not so large as to be unusual among Paleozoic fishes. The mandibular arch in CMNH 5768 is roughly 16% estimated TL (using estimates for this specimen in Engelman, 2023a, 2024), comparable to the ctenacanths *Dracopristis* (16.6% TL; Hodnett et al., 2021, fig. 12) and *Goodrichthys* (16.3% TL; Maisey et al., 2017, fig. 5), the stethacanthid symmoriform *Akmonistion* (approximately 16.3%–17.1% TL; Coates & Sequeira, 2001, figs. 1 and 15), an unnamed ischnacanthiform acanthodian (UALVP 43245, 16.9% TL; Blais et al., 2015, fig. 2a), the actinopterygian *Cheirolepis* (16.2% TL; Pearson & Westoll, 1979), the rhizodont sarcopterygians *Strepsodus* (17.7% TL; Andrews, 1985, though this material is juvenile) and *Gooloogongia* (17.5% TL; Johanson & Ahlberg, 1998), and the tristichopterid sarcopterygian *Eusthenopteron* (17.5%–22.4% TL; Andrews & Westoll, 1970; Jeffery, 1998, p. 233; Schultze, 1984). As a point of contrast, in *Coccosteus cuspidatus* (ROM VP 52667) the mandibular arch (cheek unit) is ~14.5% TL and the infragnathal is 13.2% TL, which is reflected in the shorter mandibular arch relative to the dermal skeleton. While historical methods of estimating body size in *Dunkleosteus* are often unclear (Engelman, 2023a, pp. 38–40), it seems likely that isometric scaling from the infragnathal of smaller arthrodires without considering potential interspecific variation in mandibular arch size may have been responsible for larger historical size estimates, especially as mandibular length is often used as a body size proxy in Paleozoic fishes (e.g., Ørvig, 1967). This broader similarity of relative jaw proportions across taxa agrees with previous observations that many Paleozoic piscivores have proportionally large mouths/mandibular arches compared to typical proportions in modern fishes (Engelman, 2023b).

The proportionally large and elongate mandibular arch in *Dunkleosteus* has two major effects on jaw function. First, because the origins for the adductor muscles of arthrodires are largely located between the quadratomandibular articulation and the mouth, elongation of the postorbital cheek facilitates an enlargement of the adductor fossa and its associated musculature—and thus bite force. Second, the more posterior location of the jaw joint enhances gape (Engelman, 2023b), as it creates a greater separation between the tips of the upper and lower jaw at similar gape angles. This is especially important in *Dunkleosteus* because it allows clearance between the well‐developed, “fang‐like” odontoids on the anterior supragnathal and infragnathal. Miles (1969, p. 151) proposed a similar explanation when discussing differences in relative cheek length in Scottish coccosteids. Increasing the size of the head/mandibular arch relative to a conserved body shape has been noted as a way macropredatory taxa may increase bite force and gape—and thus possible prey size—with otherwise minor alterations in body shape and morphology (Engelman, 2023b, pp. 20–21; Huber et al., 2009).

It is unlikely the more elongate mandibular arch of *Dunkleosteus* relative to head size resulted in a significant increase in bite velocity or a decrease in mechanical advantage compared to other arthrodires. Unlike many vertebrates with elongated jaws, where jaw elongation occurs via extension of the rostrum and a longer out‐lever relative to the in‐lever of the adductor muscles, intra‐mandibular arch proportions of *Dunkleosteus* do not differ greatly from other eubrachythoracid arthrodires. The oral and adductor regions of the infragnathal in *Dunkleosteus* are nearly equal in length (Engelman, 2023a, supplementary file S2), and these proportions do not appear to change significantly across ontogeny. Similar proportions are widespread among eubrachythoracids, including non‐camuropiscid incisoscutoids (Dennis & Miles, 1981, tab. 1; Gardiner & Miles, 1990, tab. 1; Gardiner & Miles, 1994: tab. 1; Miles & Dennis, 1979, p. 36), *Eastmanosteus calliaspis* (Dennis‐Bryan, 1987, tab. 2), *Hussakofia minor* (Miles, 1969), *Heintzichthys gouldii* (oral region is ~48% total infragnathal length; CMNH 5266, CMNH 6025, CMNH 6352, NHMUK PV P9335), *Steneosteus angustopectus* (Carr, 1996, fig. 8a), *Brachyosteus dietrichi*, leiostids, and leptosteids (Miles, 1969, p. 150), among others. Miles (1969, p. 150) suggests this conserved proportion reflects “the best compromise between the conflicting requirements of a powerful bite and long gnathal margins with a wide gape … in little‐specialized, predacious Brachythoraci,” which is generally supported by the observations here. Because of these conserved proportions, the relationship between the in‐lever (the adductor muscles, if their center of mass is assumed to be at the midpoint of the adductor fossa) and out‐lever (represented by the symphyseal margin/odontoid) remains consistent, and thus mechanical advantage is preserved. As noted above, the adductor fossa of *Dunkleosteus* is asymmetric and deeper anteriorly, meaning the center of mass of the adductor muscles and thus in‐lever is shifted anteriorly (~42% of infragnathal length in *D. terrelli* versus ~30% in *Compagopiscis*). However, this would be due to a shift in muscular anatomy, rather than a change in jaw length or proportions. Thus, even considering adductor chamber shape, elongation of the mandibular arch in *Dunkleosteus* would not result in decreased mechanical advantage compared to smaller arthrodires.

#### 3.1.5 Median raphe

The dorsal and ventral heads of the *m. adductor mandibulae* were likely separated by a band of connective tissue: a median raphe (sometimes referred to as a mid‐lateral raphe; Wilga, 2005). Median raphes are present or inferred in most chondrichthyans (excluding holocephalians, but including symmoriiform stem‐holocephalians; Chappell & Seret, 2021; Lane & Maisey, 2012; Motta & Wilga, 1995; Wilga, 2005), but their broader distribution in gnathostomes is unclear. The interpretation of a median raphe in *Dunkleosteus* is based on the quadrate articulation being positioned immediately posteroventral to the adductor chamber. Without a median raphe, the quadrate articulation would intersect with the body of the *m. adductor mandibulae* at anything more than very narrow gapes (Figure 4, see also “Gape” and Anderson & Westneat, 2009, pp. 259–260), making the jaws non‐functional. A similar issue is faced by chondrichthyans, which is solved in these taxa by the median raphe allowing muscle fibers of the *m. adductor mandibulae* to change their angle of insertion relative to the muscle's contractile path and thus avoid intersecting the jaw articulation (Ferguson et al., 2015; Ferrara et al., 2011, see also Figures 2 and 4). The morphology of the suborbital groove also favors a median raphe. This structure, which contains the contractile path of the *m. preorbitalis*, ends at the soft‐tissue angles of the jaws (see “Suborbital groove and *m. preorbitalis*” below, and Figures 2 and 4), where the *m. preorbitalis* inserts into the median raphe in most chondrichthyans (Motta & Wilga, 1995; Wilga, 2005), further favoring its presence in arthrodires.

### 3.2 Functional group 2: Suborbital groove and *m. preorbitalis*

**FIGURE 7:**
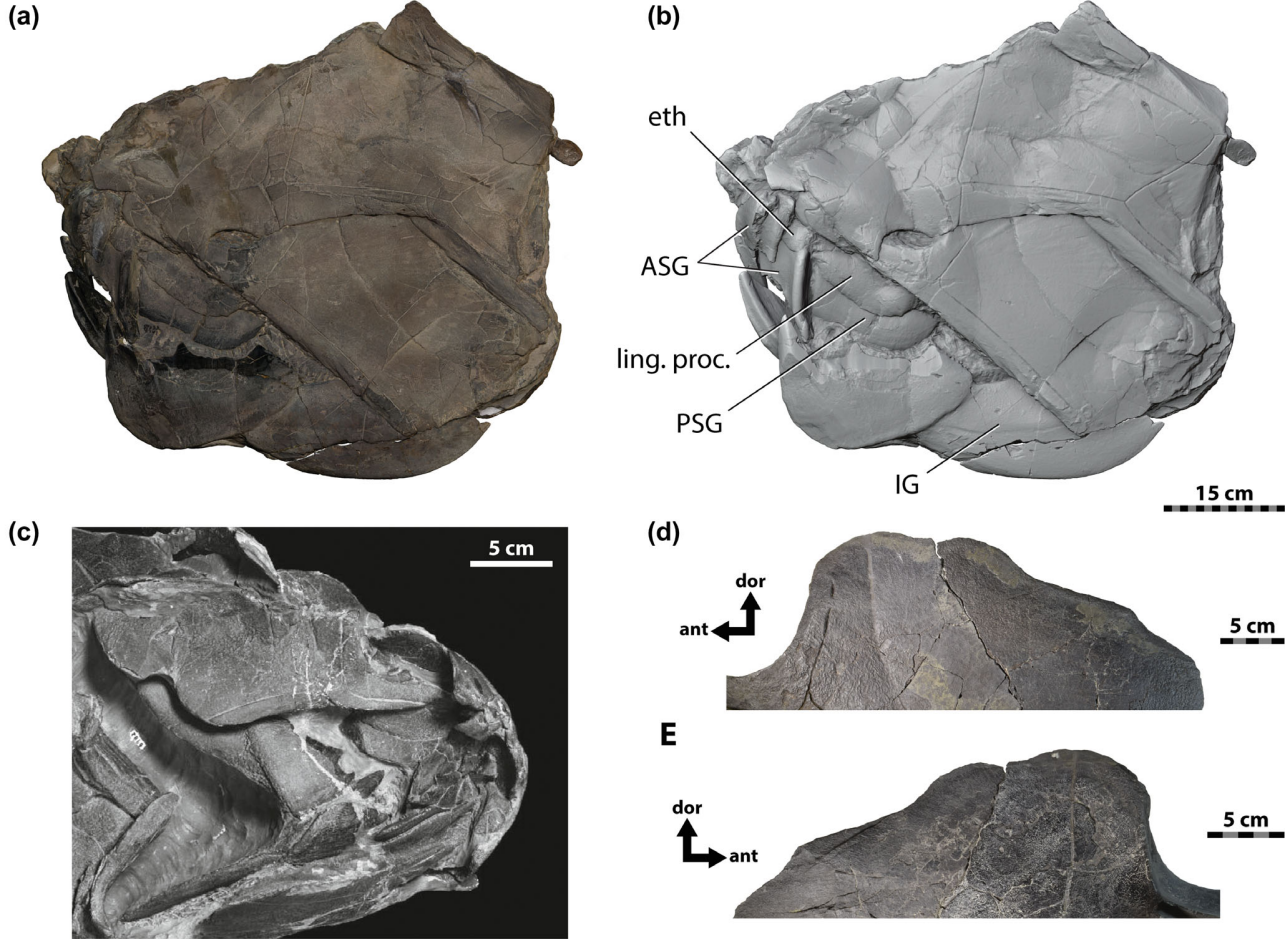
(a, b) Photograph (a) and surface scan (b) of *Dunkleosteus terrelli* (CMNH 8131), showing the internal position of the gnathals relative to the linguiform process. (c) CMNH 8982, showing the gnathals located internal to the linguiform process and suborbital groove. (d, e) Suborbital of *D. terrelli* (CMNH 9900) in external (d) and internal (e) view, showing the absence of an overlap facet for the skull roof on this plate. ASG, anterior supragnathal; eth, ethmoid ossification; IG, infragnathal; ling. proc., linguiform process of suborbital; PSG, posterior supragnathal.

The external border of the oral region in *Dunkleosteus* has a distinct, inset margin relative to the rest of the headshield. This structure, termed here the suborbital groove (Figures 3 and 9), is bordered internally by the ethmoid ossification of the braincase and linguiform process of the suborbital and dorsally by the rims of the inset regions of the suborbital and postnasal plates. In ventral view, the groove widens posteriorly and eventually merges into the adductor chamber at the approximate level of the angle of the jaws, creating a distinct shelf in this view (blue highlighted region in Figure 9). Anteriorly, the groove narrows such that it is poorly developed at the medial border of the postnasal plate and anterior supragnathals. *Dunkleosteus* specimens with gnathals preserved in situ (CMNH 8131, CMNH 8982; Figure 7a–c) demonstrate the posterior supragnathals articulate on the autopalatine portion of the palatoquadrate, situated on the internal face of the linguiform process, as in other eubrachythoracids. This indicates the suborbital groove is a structure external to the gnathals (Figure 7a–c), constraining the possible structures that could have filled it.

The suborbital groove of *Dunkleosteus* is very large and well‐developed, being 2–3 cm in width or more in larger specimens (CMNH 6090, CMNH 7054, CMNH 5768) and 4–6 cm in width where it meets the adductor fossa. Suborbital grooves are not unique to *D. terrelli* but are present to some degree in all eubrachythoracids (e.g., Gardiner & Miles, 1990, fig. 21; Miles & Dennis, 1979, fig. 12). Their presence in non‐eubrachythoracids is more difficult to evaluate because of the limited jaw material available. However, in buchanosteids there is a small but distinct suborbital groove separating the supragnathals from the external margin of the suborbital plate (Hu et al., 2017, p. 8, fig. 2b; Young et al., 2001, p. 673); though a linguiform process is absent. Similarly, in *Dicksonosteus*, the supragnathals are inset from the margin of the head shield (Goujet, 1984, pp. 109–110, fig. 50) and there is a continuous inset margin, bordered dorsally by the ventral face of the postnasal plate (similar to the condition in eubrachythoracids), extending between the adductor chamber and the nasal capsule (Goujet, 1984, fig. 50). This suggests suborbital grooves may be widely distributed among Arthrodira.

**FIGURE 8:**
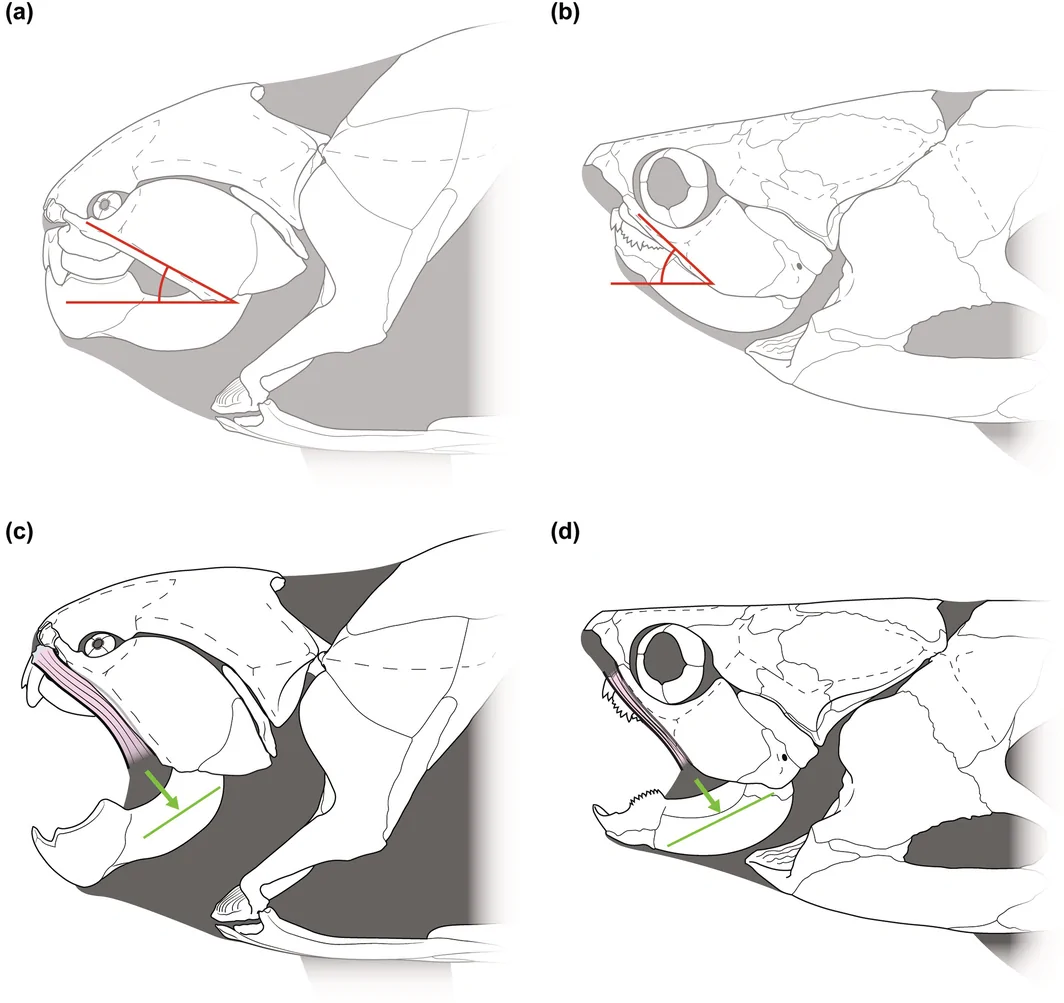
Heads of *Dunkleosteus* (a, c; CMNH 6090) and *Compagopiscis* (b, d; modified from Gardiner & Miles, 1994 and WAM 70.4.263) showing the more oblique angle of the suborbital groove/mouth when the mouth is closed and smaller linguiform process of *Compagopiscis* (a, b) and the location and angle of pull of the *m. preorbitalis* (c, d) when the mouth is at maximum gape.

Although arthrodires show significant variation in the shape and size of the suborbital groove and associated linguiform process, *Dunkleosteus terrelli* consistently stands out among eubrachythoracids in these structures being unusually large in both relative and absolute size. This is best demonstrated by the relative development of the linguiform process. In most arthrodires, the linguiform processes are very small and often barely protrude externally beyond the dermal face of the suborbital plate (Figure 8b, File S3A,B,E; see also Dennis & Miles, 1979b, fig. 11b; Dennis & Miles, 1980, fig. 18; Dennis & Miles, 1981, fig. 7a,b; Gardiner & Miles, 1990, fig. 8; Miles & Dennis, 1979, fig. 12a; Miles & Westoll, 1968, fig. 13; Stensiö, 1963, among others). By contrast, in *Dunkleosteus*, the linguiform processes are large and tall, protruding orally a significant distance from the dermal face of the suborbital (Figure 8a, see also Figure 2, ling. proc.). In addition, the suborbital grooves in many of these other eubrachythoracids are more ventrally opening. By contrast, the suborbital groove of *D. terrelli* is dorsoventrally deeper and more open externally due to the large, projecting linguiform process bordering it internally. In at least some of these arthrodires, the suborbital groove may be partly formed by the autopalatine (Miles & Dennis, 1979, fig. 12), but in *D. terrelli*, the extent of the linguiform process and its close association with the gnathals in situ (Figure 7c) suggests most of the groove was formed by the suborbital. The large size of the linguiform process in *D. terrelli* cannot be attributed purely to allometry, as other large eubrachythoracid arthrodires such as *Heintzichthys* (CMNH 5266, CMNH 6352; see also Carr, 1991), *Alienacanthus* (Jobbins et al., 2024, fig. 2a), *Bungartius* (CMNH 6666, CMNH 7573), *Tafilalichthys* (Lehman, 1956, p. 20), and *Titanichthys* (CMNH 50319; Boyle & Ryan, 2017, fig. 7, p. 326) have very small linguiform processes and narrow suborbital grooves. The condition of the linguiform process cannot be evaluated in the large, macropredatory arthrodires *Gorgonichthys*, *Dinichthys*, or “*Dunkleosteus*” *magnificus*, as the suborbital plate is unknown in these taxa (Carr & Hlavin, 2010; Dunkle & Bungart, 1946; Hussakof & Bryant, 1918). The linguiform process of *Dunkleosteus* is significantly larger and more protruding than almost any other arthrodire. The taxon most closely resembling the condition in *Dunkleosteus* is the dunkleosteoid *Eastmanosteus calliaspis*, in which the linguiform process is large and orally protruding but to a slightly less extreme degree (Dennis‐Bryan, 1987, fig. 13; but see Jobbins et al., 2025, regarding phylogenetic placement).

Unlike the rest of the external face of the suborbital plate, the linguiform process of arthrodires lacks dermal ornamentation, indicating it was overlain by soft tissue in life (Stensiö, 1934). The sheer size of the suborbital groove in *Dunkleosteus* indicates this structure likely contained soft tissue structures beyond simple oral epithelium. This groove is so extensive in width that the gnathals are positioned significantly inset to the skull margin (Figure 10). If this region was not filled with soft tissue, it would disrupt streamlining of the animal in viv and potentially create serious drag. These issues are present to a lesser extent in smaller arthrodires (compare the sunken area of the face corresponding to this region in Long, 1988, fig. 15), but are much more obvious in *Dunkleosteus* due to the greater size of the suborbital groove. It is unlikely the groove was occupied by oral epithelium alone because most fishes (as well as most non‐mammals) have a thin, non‐muscularized oral epithelium that does not greatly protrude beyond the boundaries of the underlying skeletal or soft tissue structures and typically does not produce excavations of the magnitude seen in the skull of *Dunkleosteus*. Thick, highly muscularized lips, as seen in mammals, are mostly dermal musculature apomorphically derived from the *m. interhyoideus* of lower vertebrates and are generally not present in non‐mammals (Diogo & Powell, 2019; Mallatt, 1996).

Based on other taxa, there are two possible options for the soft tissue structures occupying the suborbital groove: (1) well‐developed labial cartilages or (2) an antorbital jaw muscle akin to the *m. preorbitalis* of chondrichthyans. These are not mutually exclusive, as the labial cartilages are typically embedded in and mobilized by the *m. preorbitalis* in chondrichthyans (Anderson, 2008a; Dearden et al., 2021; Wu, 1994), but see below. Of the two possible options, an antorbital jaw muscle seems more likely. Labial cartilages are unknown in the large (600+ specimens) hypodigm of *Dunkleosteus terrelli*, including articulated specimens (CMNH 8982, CMC VP 8543, CMC VP 8924) that have the gnathals in life position, preserve other perichondrally ossified elements, and even preserve soft tissue traces (Carr et al., 2010; Engelman, 2024, fig. 19; Storrs et al., 2008). Taphonomic evidence suggests the gnathal plates of *D. terrelli* readily disarticulated from the body during decay, likely because they articulated with the head shield and trunk armor across cartilage (Carr, 2010). If large labial cartilages were present in *D. terrelli*, it would be highly unusual for these cartilages to be consistently lost while, at the same time, the gnathal plates, another set of oral structures that readily disarticulate from the dermal skeleton, are preserved in situ. Indeed, labial cartilages are not known in any eubrachythoracid arthrodire, even those from localities such as the Gogo Formation or the Orcadian Basin of Scotland (e.g., Miles & Westoll, 1968) where multiple, near‐complete specimens have been recovered with parts of the perichondral/cartilaginous skeleton preserved. This is despite labial cartilages being preserved in other fish taxa from the same localities (ptyctodonts; Trinajstic et al., 2012). Moreover, the anterior extent of the groove means the cartilages would potentially block the gnathal plates when the mouth was open (see “Was *Dunkleosteus* a suction feeder” below for a more extensive discussion).

Prior references to possible labial cartilages in eubrachythoracid arthrodires are speculative. Stensiö (1963) restored arthrodires with labial cartilages, but these were described as hypothetical and based on assumptions of a chondrichthyan‐like morphology rather than the observation of fossilized labial cartilages in situ (Stensiö, 1963, pp. 263–268). Miles and Westoll (1968, pp. 418–419) suggested that *Coccosteus* had “broad, fleshy labial folds … supported at the gape by labial cartilages,” but this was based on the existence of the suborbital groove and inset position of the gnathals, as well as following the interpretations of Stensiö (1963). Fossil evidence that the suborbital groove specifically held labial cartilages was not presented—and as discussed below this feature has alternative possible interpretations. While it remains possible that some small, poorly mineralized labial cartilages were present and provided structure to the soft tissue angle of the jaws, as in polypterids (Allis Jr., 1919) and large macropredatory sharks (Klimpfinger & Kriwet, 2023), well‐developed labial cartilages in arthrodires seem unlikely at this time.

**FIGURE 9:**
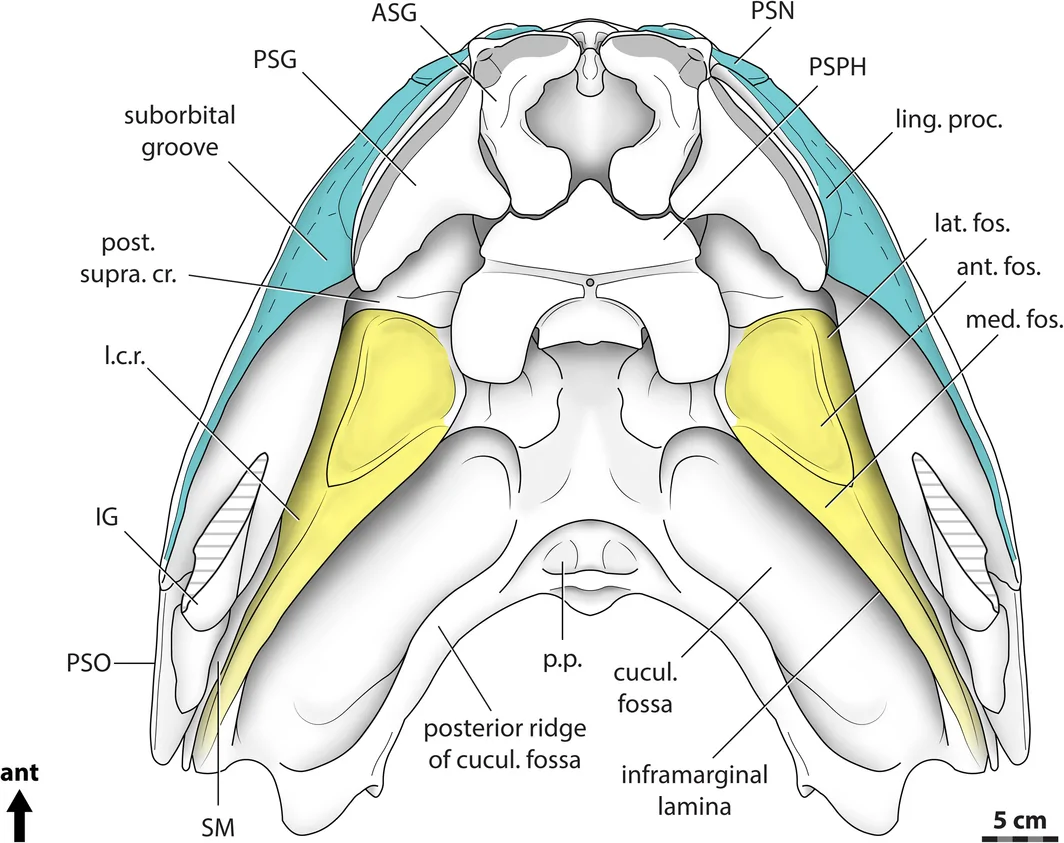
Skull of *Dunkleosteus* in palatal view, showing the suborbital groove (in teal) and extremely large lateral consolidated region of the skull (in yellow). Skull roof sutures are not delineated, and no attempt was made to reconstruct jaw cartilages due to preservation. Skull mostly modeled after CMNH 6090, with missing details partially restored after CMNH 6194, CMNH 7054, Heintz (1932), and Dunkle and Bungart (1946). Solid gray areas represent wear facets on the supragnathals. The shape of the grooves on the ventral face of the parasphenoid are unclear and partially reconstructed. There is some evidence these structures were present, but specimens are often broken or reconstructed in this area, so the exact morphology is uncertain. Abbreviations follow Figures 1, 2, and 6 with the addition of post. supra. cr., posterior supraorbital crista; p.p., paired pits on the visceral surface of the nuchal plate; and PSN, postnasal plate.

Several features suggest the suborbital groove contained a muscle likely homologous with the *m. preorbitalis* of chondrichthyans (= *m. levator labii superior* of some authors; Dearden et al., 2021; Soares & de Carvalho, 2013). The endpoints of the suborbital groove are nearly identical to the origin and insertion of the *m. preorbitalis* in most chondrichthyans, which are located on the ectethmoid process and median raphe at the angle of the jaws, respectively (Dearden et al., 2021; Mallatt, 1996; Motta & Wilga, 1995; Soares & de Carvalho, 2013; Wilga, 2005; Wu, 1994, though some variation exists within this group). The ethmoid ossification of arthrodires represents the anterolateral corner of the ventral part of the ethmoid, and thus based on position, appears to be homologous with the ectethmoid process of chondrichthyans (Carr, 2004a; Dupret, 2010; Goujet, 1984; Stensiö, 1963; Zhu, Zhu, & Wang, 2016), implying the potential endpoints for these muscles are homologous. Although the suborbital groove grades toward the adductor chamber from its external side (Figure 9), it is separated from the adductor fossa dorsally (Figure 3b). This is very similar to the positional relationship between the *m. preorbitalis* and *m. adductor mandibulae* in sharks, in which the former is a superficial muscle that attaches to the *m. adductor mandibulae* anteroexternally (Datovo & Johnson, 2025; Mallatt, 1996, 1997). The structure occupying the suborbital groove appears to have been innervated by the maxillary or possibly the mandibular branch of the trigeminal nerve (CNV2 and CNV3), as Long (1988) describes several foramina opening into the suborbital groove of *Latocamurus* that the author considered to represent foramina for these nerves. This, again, resembles extant chondrichthyans, in which the *m. preorbitalis* is innervated by these nerves (Dearden et al., 2021; Diogo et al., 2018; Ziermann et al., 2014).

Although the *m. preorbitalis* has been most extensively studied in chondrichthyans, homologs of this muscle may be more widely distributed. Anderson (2008a) suggests that a homologous preorbital facial muscle may be plesiomorphic for gnathostomes, whereas Ziermann et al. (2014) conclude that at least some labial muscles (including potentially an *m. preorbitali*s) were likely present in the last common ancestor of Gnathostomata. The *m. preorbitalis* has been suggested to be homologous with the buccal constrictor of lampreys (Mallatt, 1996, 1997; Ziermann et al., 2014), potentially making arthrodires bracketed by taxa possessing this muscle. However, the homologies of any oral muscles between “agnathans” and gnathostomes remain contentious (Miyashita et al., 2025). Other authors have considered the *m. preorbitalis* to represent the most anterior division of the *m. adductor mandibulae* (Datovo & Johnson, 2025; Lauder Jr., 1980; see discussion in Diogo & Abdala, 2010, pp. 205–206). Still other studies have considered the *m. preorbitalis* a labial muscle distinct from the *m. adductor mandibulae*, yet not homologous with the oral constrictors of cyclostomes (Diogo et al., 2018, p. 71; Ziermann et al., 2014). Developmental studies in *Scyliorhinus* find the *m. preorbitalis* begins development close to the anlange of the *m. adductor mandibulae* (Ziermann et al., 2017, p. 7), which could support an adductor identity, though at the same time that study refers to the *m. preorbitalis* as a labial muscle rather than explicitly as part of the *m. adductor mandibulae* (Ziermann et al., 2017, tab. 1). Fate‐mapping experiments in lamprey (Kuratani et al., 2004, p. 466) report “a part of the mandibular mesoderm secondarily grows anteriorly and laterally and migrates into the upper lip domain,” resembling the developmental path of the *m. preorbitalis* in sharks. However, Kuratani et al. (2004) explicitly reject homologies between the upper lip of lamprey ammocoetes and gnathostomes due to differences in innervating motor neurons and suggest the labial muscles in these taxa are due to developmental pathways unique to both groups.

Because the *m. preorbitalis*/*m. levator labii superior* in chondrichthyans supports and mobilizes the labial cartilages (Anderson, 2008a; Dearden et al., 2021; Motta & Wilga, 1999), the presence of the latter may depend on the former (though not vice versa; Klimpfinger & Kriwet, 2023; Wilga, 2005). This means that the presence of labial cartilages in ptyctodonts (Trinajstic et al., 2012) and *Ramirosuarezia* (Pradel et al., 2009) might imply a *m. preorbitalis*‐like muscle may have been widely distributed in stem‐gnathostomes. Given suggestions that labial cartilages might be plesiomorphic for gnathostomes and homologous with oral hood cartilages in cyclostomes (Lund & Grogan, 1997), the association between the *m. preorbitalis* and labial cartilages might imply an antorbital muscle + labial cartilage complex derived from “agnathan” oral elements may be symplesiomorphic for gnathostomes. An oral groove resembling the suborbital groove of arthrodires is present on the suborbitals of euantiarchs and has also been interpreted as supporting soft tissues associated with the upper lip (Lebedev et al., 2022; Stensiö, 1948; Young, 1984). However, it is unclear if it is homologous with the suborbital groove of arthrodires, as it is significantly narrower and not present in yunnanolepidoids (unless the groove interpreted as the adductor fossa is partially homologous with this structure; Wang & Zhu, 2021).

The presence of a *m. preorbitalis* in osteichthyans is contentious. Some authors have proposed the antorbital muscles of actinopterygians are homologous with the chondrichthyan *m. preorbitalis* (Datovo & Johnson, 2025; Lauder, 1982; Lauder Jr., 1980), but others have disputed this (Datovo & Rizzato, 2018; Diogo et al., 2018). Millot and Anthony (1958) and Anderson (2008a) report an upper lip muscle in coelacanths (*m. levator labii superioris*), which Anderson (2008a) homologized with the *m. preorbitalis* of chondrichthyans. However, later studies did not identify this muscle (Datovo & Johnson, 2025) and instead proposed a postorbital branch of the *m. adductor mandibulae* (*pars orbitalis*) was homologous with the *m. preorbitalis*, similar to what had been proposed for actinopterygians. A major issue to homologizing these muscles to the *m. preorbitalis* of chondrichthyans is that the latter is superficial to the main mass of the *m. adductor mandibulae* and originates anteroexternal and ventral to the orbit, whereas the proposed homolog in osteichthyans is deep to much of the *m. adductor mandibulae* and typically has a postorbital origin. For arthrodires, given the strong similarities in tissue position and seemingly homologous origins and insertions to the antorbital muscles of chondrichthyans (as well as avoid contributing to the profusion of different names for homologous muscles across gnathostones; Anderson, 2008a; Datovo & Johnson, 2025; Diogo & Abdala, 2010), tentatively treating them as functionally equivalent and potentially homologous seems the best available option at present, though the latter needs further evaluation.

**FIGURE 10:**
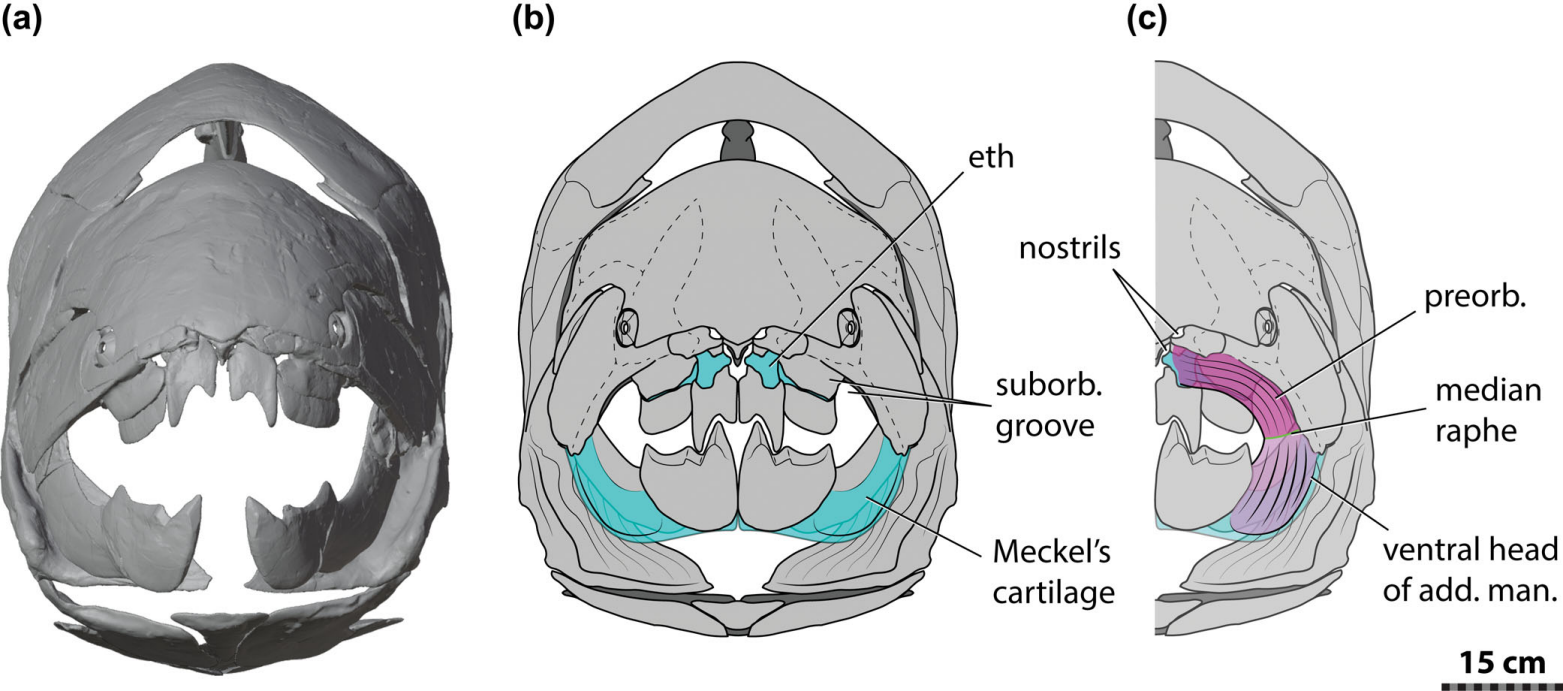
Retrodeformed specimen of *Dunkleosteus terrelli* (CMNH 6090) in anterior view. (a) 3D scan of specimen as mounted. (b) Anatomy corrected for deformation. (c) Specimen with musculature restored. While the mount may have been retrodeformed, the great depth of the suborbital groove is natural. Abbreviations follow Figure 2.

**FIGURE 11:**
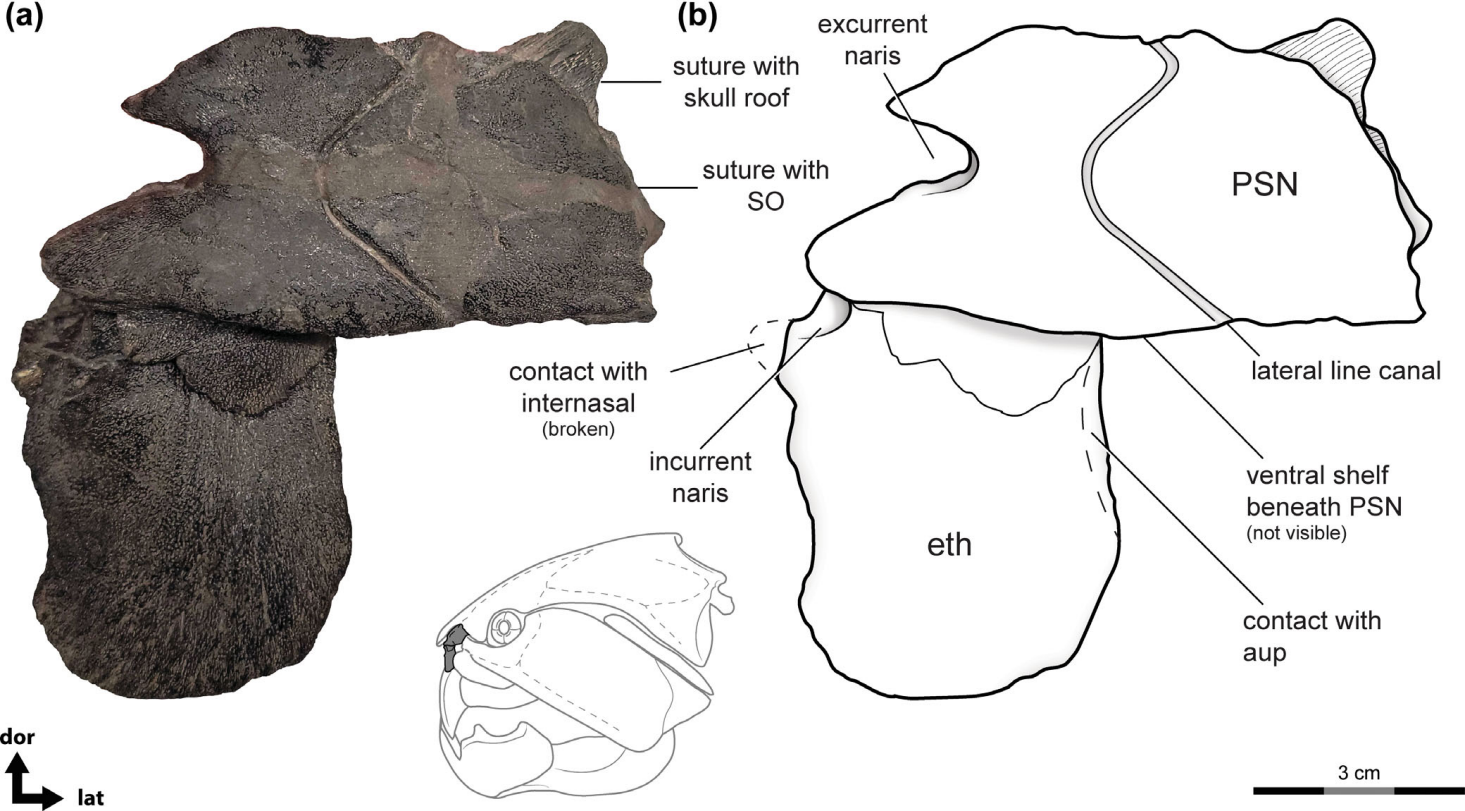
Photograph (a) and interpretive drawing (b) of a left postnasal and ethmoid ossification of a large (~3.5 m) individual of *Dunkleosteus terrelli* (CMNH 7568). Abbreviations as in Figure 2.

**FIGURE 12:**
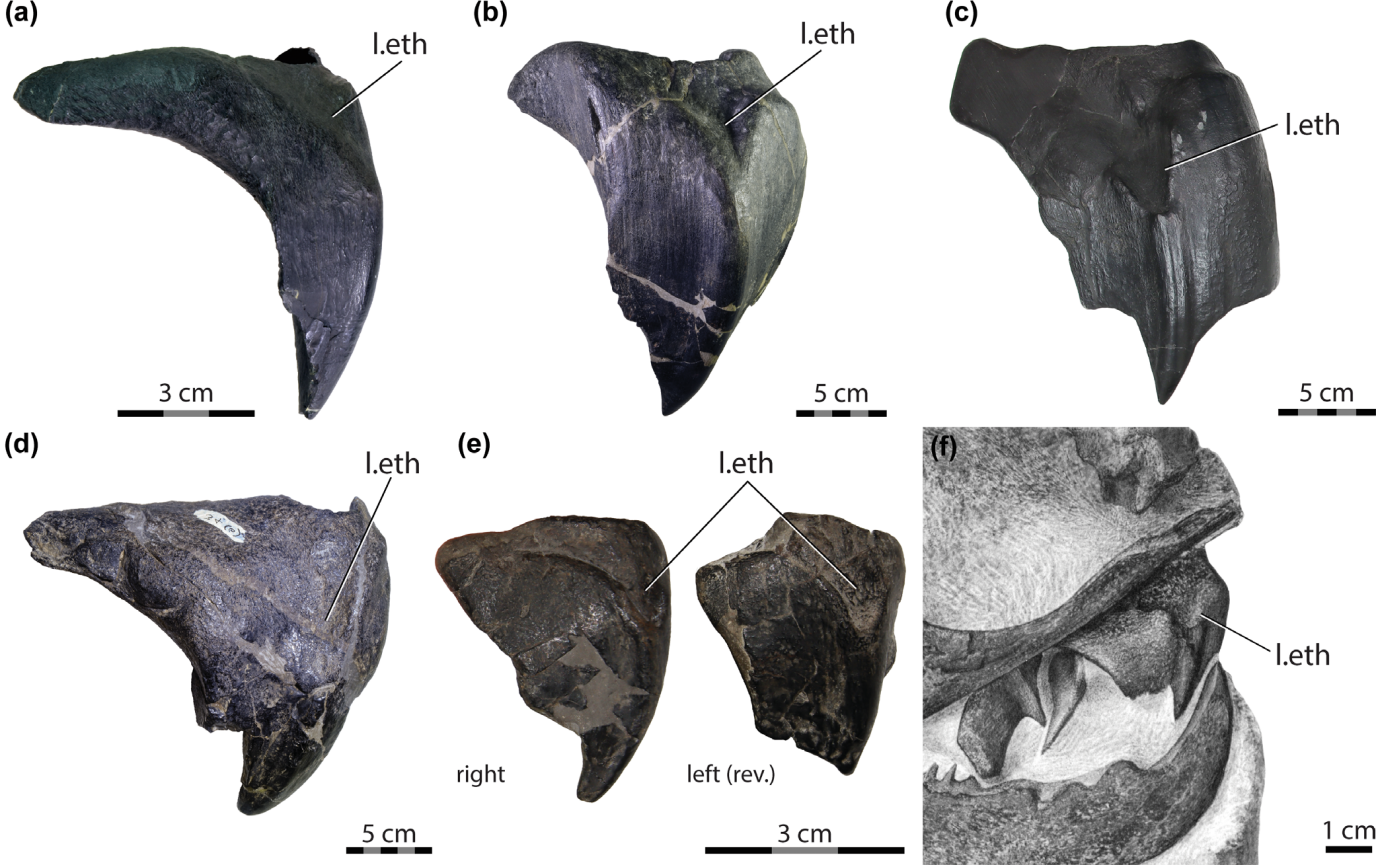
Anterior supragnathal in macropredatory arthrodires, showing the enlarged, ventrally descending contact for the ethmoid (l.eth). (a, b) *Dunkleosteus terrelli* in lateral (a, CMNH 8319 [reversed]) and oblique anterolateral (b, CMNH 5984) views; (c) *Gorgonichthys clarki* (CMNH 7129); (d) *Dinichthys herzeri* (AMNH FF 34, reversed); (e) *Heintzichthys gouldii* (CMNH 5266, right and left [reversed]); (f) *Hadrosteus rapax* (MB.f.163), modified from Stensiö (1963, pl. 21.3). Anterior is to right in all images.

The *m. preorbitalis* in *Dunkleosteus* appears to have originated on the ethmoid ossification and the flattened shelf on the ventral face of the postnasal (Figures 10 and 11). The ethmoid ossification has a corrugated articulation with a ventrally projecting process of the postnasal dorsally, contacts the linguiform process of the suborbital laterally, and fits into a triangular fossa on the dorsal surface of the anterior supragnathal ventrally. In one specimen (USNM V 21314) the ethmoid ossifications also articulate with the internasal plate medially, with the internasal (or possibly the more medial part of the ethmoid) showing a pair of pronounced lateral excavations (https://doi.org/10.6084/m9.figshare.29330135) that appear to form the medial border of a continuous fossa between this plate, the ventral surface of the postnasal, and the external surface of the ethmoid (though this could be an artifact of restoration as its presence cannot be confirmed in other specimens and the internasal plate of USNM V21314 could not be examined in close detail due to being on exhibit). The ethmoid of *Dunkleosteus* differs from most non‐macropredatory arthrodires in having a strongly developed, lip‐like ventral projection descending down the anterolateral face of the anterior supragnathal (Figure 12, File S7A,B). This feature is present throughout ontogeny in the CMNH *Dunkleosteus* hypodigm, even in some of the smallest examined individuals of *D. terrelli* (CMNH 6194, File S7). By contrast, this ventrally extending contact is absent in both juveniles (Dennis‐Bryan, 1987, fig. 18a,b) and adults (Dennis‐Bryan, 1987, fig. 19a–d) of *Eastmanosteus calliaspis* and seemingly also *Kiangyousteus yohii* (Zhu & Zhu, 2013, fig. 3c) but is present in *E. pustulosus* (Hussakof & Bryant, 1918, p. l. 12.1) and seemingly *Elmosteus lundarensis* (Jobbins et al., 2025, fig. 6a,c). Although anterior supragnathals are known for “*Dunkleosteus*” *magnificus* and “*D.” newberryi*, the state of its contact with the ethmoid cannot be determined based on available photos (Hussakof & Bryant, 1918). It is also present, seemingly convergently, in the macropredatory aspinothoracidans *Gorgonichthys clarki* (CMNH 7129, 7288; Figure 12c) and *Dinichthys herzeri* (AMNH FF 34; Figure 12d). A less extreme version of this extended contact occurs in *Heintzichthys gouldii* (CMNH 5266; Figure 12e) and *Hadrosteus rapax* (Figure 12f). The morphology of this extension differs slightly between dunkleosteoids and macropredatory aspinothoracidans; in the former the external extension is shallow and formed by the anterolateral corner of the contact tipping ventrally (Figure 12a; Hussakof & Bryant, 1918), whereas in the latter it is deeply incised and often sharply defined relative to the rest of the anterior supragnathal–ethmoid contact (Figure 12c–f). This projection of the ethmoid appears to be the origin for the *m. preorbitalis* in *D. terrelli*. It is also possible the *m. preorbitalis* could have partially originated on the external surface of the linguiform process given this structure forms the internal border of the suborbital groove, but this is unclear given the morphology of this structure.

As mentioned above (under “median raphe”), the *m. preorbitalis* most likely inserted onto a median raphe, given the suborbital groove widens and ends roughly at the level where the boundary of the two heads of the *m. adductor mandibulae* would be. However, one alternative possibility must be mentioned. In some squalomorph elasmobranchs, the insertion of the *m. preorbitalis* has shifted such that it passes medial to the *m. adductor mandibulae* and inserts via a tendon onto the mandible (Diogo et al., 2018, p. 67; Wu, 1994). This is worth mentioning because of the rugose, coronoid‐like process observed on the infragnathal of *Dunkleosteus* (Lebedev et al., 2023), which implies some kind of soft tissue structure (part of the *m. adductor mandibulae*, a ligament, a tendon, etc.) attached medial to the adductor chamber. It is possible this could represent an alternative origin for the *m. preorbitalis* or a branch of the *m. preorbitalis* if this muscle was subdivided as seen in many living chondrichthyans (Diogo et al., 2018; Wilga, 2005). However, we do not favor this interpretation, as the coronoid‐like process is least prominent in larger specimens of *Dunkleosteus terrelli*, whereas the suborbital groove becomes larger, and the posteriorly widening shape of the suborbital groove would suggest a broad attachment onto the adductor mass, as has been described for lamniforms and carcharhiniforms (Wilga, 2005; Wu, 1994). Nevertheless, we note this possibility as it may help future workers understand the anatomy of *Dunkleosteus*.

In chondrichthyans, for which the function of the *m. preorbitalis* has been most extensively studied, this muscle serves one of three functions: protraction of the palatoquadrate, mobilizing the labial cartilages, and closing the jaws (Dearden et al., 2021; Diogo et al., 2018, p. 37; Moss, 1972; Soares & de Carvalho, 2013; Wilga, 2005; Wu, 1994), often performing multiple roles or being split into multiple heads with different functions. Given the lack of evidence for labial cartilages in arthrodires, the first function can be ruled out for *Dunkleosteus terrelli*. Protraction of the cheek unit via the *m. preorbitalis* was also not possible. The cheek unit of *Dunkleosteus* is heavily integrated: the postnasal and suborbital plates articulate via an interdigitating suture (Figure 11), the anterior and posterior supragnathals are in close physical contact, and the autopalatine region of the palatoquadrate articulates with the ethmoid ossification and suborbital, likely filling the small gap between the ethmoid ossification, suborbital, and postnasal visible in external view. This means contraction of a muscle originating on the ethmoid and postnasal ethmoid would not move or rotate the rest of the cheek unit.

The path of the inferred *m. preorbitalis* suggests this muscle functioned in jaw closure in eubrachythoracid arthrodires. Indeed, given the presence of this muscle in several taxa with non‐protractable jaws (e.g., non‐neoselachian chondrichthyans and *Chlamydoselache*; see Datovo & Johnson, 2025 for the latter), previous suggestions that preorbital labial muscles characterized the most recent common ancestor (MRCA) of Gnathostomata, and its possible homology with the buccal constrictor of lampreys, mouth closure and/or oral constriction may have been its original function. This agrees with Wu (1994), who found an inverse relationship between the size of the *m. preorbitalis* and degree of jaw protraction in orectolobiform sharks (a group known for highly protrusible jaw kinematics) and concluded the primary function of the muscle in that group was probably jaw closure. The location and contractile path of the *m. preorbitalis* in arthrodires is almost ideally situated to compensate for the problem of low mechanical advantage of the *m. adductor mandibulae* at high gape angles. Mechanical advantage of the *m. adductor mandibulae* is high when the mouth is nearly closed but decreases as the jaws open due to the increasing angle between the palatoquadrate (and thus adductor fossa) and mandible. This is a particular problem for taxa such as *Dunkleosteus* due to its wider gape. The shape of the palatoquadrate in *Dunkleosteus* allows the anterior fibers of the *m. adductor mandibulae* to be more verticalized when the mouth is open, but the posterior parts of this muscle still have to pull at an oblique angle. This solution would also not be possible for other arthrodires which lack the specialized palatoquadrate morphology of *Dunkleosteus*. However, the position of the suborbital groove means mechanical advantage of the *m. preorbitalis* increases as that of the *m. adductor mandibulae* decreases. This would allow the *m. preorbitalis* to initiate the process of jaw closure at wide gapes, with the *m. adductor mandibulae* increasingly taking over this function at lower angles where its mechanical advantage is higher.

Our reconstruction finds that the *m. preorbitalis* of *Dunkleosteus* is capable of applying maximum torque when the jaws are near their maximum gape (Figure 8c,d), as the line of action for this muscle is oriented at a 90° angle to the long axis of the lower jaw (the ideal angle of pull for rotary force; Kirby & Roberts, 1985). Although the morphology of the suborbital groove is variable, a comparable situation appears to occur across arthrodires more generally, with variation in the morphology of the suborbital groove correlating with interspecific variation in mouth size. *Dunkleosteus* has a particularly large mouth among arthrodires (Engelman, 2023b) and its suborbital groove is oriented at a low angle to the anteroposterior axis of the body (Figure 8a). This allows the *m. preorbitalis* to retain a more perpendicular (= more ideal angle of pull) orientation to the lower jaw at larger gapes (Figure 8c). By contrast arthrodires like *Coccosteus* or *Compagopiscis*, which have smaller mouths (Engelman, 2023b), have a more obliquely oriented suborbital groove (Figure 8b), and thus the optimal angle of pull in these taxa is achieved at narrower gapes (Figure 8d).

The large size of the linguiform process and the resulting depth of the suborbital groove suggests a very large *m. preorbitalis* in *Dunkleosteus*, especially compared to other arthrodires (Figure 8). This may also be the reason for the prominent anteroventrally descending lip‐like extension of the ethmoid in this taxon, resulting in a greater surface area for the origin of this muscle along the external face of the snout. One possibility is this is related to allometry, with a proportionally larger muscle required to initiate jaw closure of a more massive head, as cranial mass will scale cubically with head length assuming geometric similarity. Another possibility is this is due to the wider gape of *Dunkleosteus*, as the *m. preorbitalis* would be required to provide a greater amount of the jaw closing torque at higher gape angles due to the lower mechanical advantage of the *m. adductor mandibulae*. In addition, although its primary function was likely initiating jaw closure, the *m. preorbitalis* probably also contributed to bite force along with the *m. adductor mandibulae*. These interpretations are supported by the independent evolution of an anterolaterally descending lip of the ethmoid in macropredatory aspinothoracidans, based on the contact for this element on the anterior supragnathal. Carr (2004a) suggested the anteroventral expansion of the anterior supragnathal/ethmoid contact was related to providing a more extensive attachment to support the large anterior supragnathals, though these explanations are not mutually exclusive.

The potential presence of an *m. preorbitalis* in arthrodires has been mentioned by some prior authors, though often only in passing. Miles (1969, pp. 137–138) considered an *m. preorbitalis* to be absent but concluded this solely on the absence of elasmobranch‐like jaw protraction in arthrodires; the possibility of an *m. preorbitalis* solely involved in jaw closure was not considered. Stensiö (1963, p. 376) considered an *m. preorbitali*s present in placoderms but does not discuss its morphology or reasoning for its presence beyond suggesting it was absent in aspinothoracidans with a fixed cheek plate. Long (1995) considered the *m. preorbitalis* (= *m. suborbitalis* of this author) to be present in *Mcnamaraspis* based on a “slender suborbital division of bone on the suborbital plate” (Long, 1995, p. 54). He proposed the *preorbitalis* served to protract the entire cheek unit and acted as an antagonist for the *m. levator palatoquadrati*, with the opposing action of the two muscles used to pump water over the gills. He also suggested the *preorbitalis* originated on a midline raphe formed by its bilateral counterpart. However, this is unlikely in *Dunkleosteus* as the suborbital groove is very narrow anteriorly, and if the *m. preorbitalis* extended beyond the ethmoid ossification, it would potentially block the more ventral pair of nostrils. Other authors do not discuss the *m. preorbitalis* in their functional models (Anderson & Westneat, 2009; Dunkle & Bungart, 1945; Heintz, 1932).

### 3.3 Functional group 3: Lateral consolidated region and *m. levator palatoquadrati*

**FIGURE 13:**
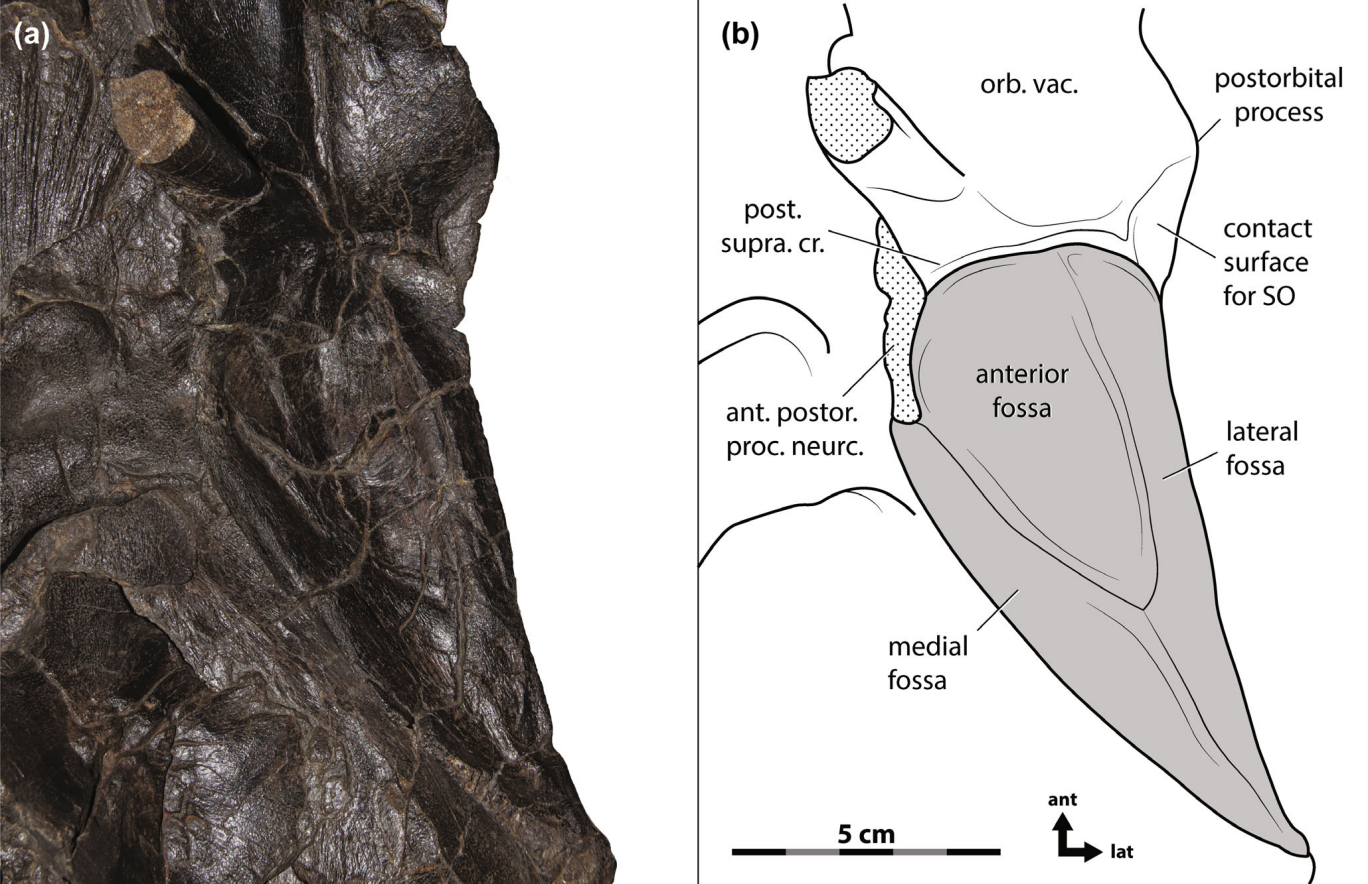
Photograph (a) and line drawing (b) of the left lateral consolidated region of *Dunkleosteus terrelli* (CMNH 5831) in ventral view, showing the subdivision of the surface into multiple fossae. Orb. vac., orbital vacuity; other abbreviations follow Figures 2 and 6.

The ventral surface of the head shield in most arthrodires has a large, laterally positioned triangular fossa termed the “lateral consolidated region” (sensu Heintz, 1932; Miles & Dennis, 1979; Miles & Westoll, 1968) located posterior to the orbital vacuity and separated from it by the posterior supraorbital crista (see area shaded in yellow in Figure 9). This surface in *Dunkleosteus* is divided into three fossae (Figures 9 and 13). The anterior fossa is located directly posterior to the orbital vacuity and is more deeply excavated than the other two fossae. The lateral fossa is the narrowest of all three and extends the length of the entire lateral consolidated region from the posterior supraorbital crista/contact surface for the suborbital plate anteriorly to the end of marginal/postmarginal plates posteriorly. The medial fossa begins more posteriorly but, like the lateral fossa, also ends at the posterior end of the lateral consolidated region. The lateral and medial fossae meet approximately at the level of the internal contact between the nuchal and central plates and remain separated by a slight ridge all the way to the end of the marginal/postmarginal plates. This results in the combined areas of the lateral and medial fossae being V‐shaped, encircling the more deeply excavated anterior fossa posteriorly (Figure 13). This tripartite division is present in every *Dunkleosteus* head shield examined (CMNH 5768, CMNH 5831, CMNH 7524, among others) and can be distinguished by differences in bone texture, suggesting it is not an artifact of crushing.

Similar arrangements may be present in other arthrodires. For example, the triangular postorbital concavity of many camuropiscids (Dennis & Miles, 1979a, 1979b; Long, 1988) is in an appropriate location to be homologous with the anterior fossa of *Dunkleosteus*. *Westralichthys uwagedenis* shows a clear *Dunkleosteus*‐like complexity to the lateral consolidated region, with a pronounced ridge on its surface resembling the ridge dividing the medial and lateral fossae (Long, 1987). Whether an anterior fossa was present is unclear; Long (1987) describes an anterior articular surface in *Westralichthys* for the *m. levator palatoquadrati*, but this structure is positioned more medial than the anterior fossa in *Dunkleosteus—*it may represent part of the rugosity for the anterior postorbital process of the neurocranium (Long, 1987, fig. 5). The dunkleosteoid *Golshanichthys* may have a tripartite lateral consolidated region with distinct anterior, lateral, and medial fossae (Lelièvre et al., 1981, fig. 2; Long, 1987), though the quality of available images makes this difficult to confirm. A medial ridge on the lateral consolidated region was also noted for *Kendrickichthys* and *Bullerichthys* (Long, 1987, p. 534), which Long (1987) proposed was a convergent development related to high bite forces in these durophagous taxa and “dinichthyids” sensu lato. The lateral consolidated region of at least one specimen of *Elmosteus lundarensis* (Jobbins et al., 2025, fig. 3c) also shows a ridge similar in morphology and position to that separating the anterior and medial fossae in *D. terrelli*.

The strongest evidence for this arrangement being more widely distributed in arthrodires can be seen in *Mcnamaraspis*, which appears to show a tripartite lateral consolidated region similar to what is described here for *Dunkleosteus*. The lateral consolidated region of *M. kaprios* is dominated by a triangular depression occupying the anterior and central parts of the lateral consolidated region (Long, 1995, p. 46, 53–54) bounded by two additional, narrow, less depressed contact surfaces encircling this fossa posteriorly and from the sides and extending to the posterolateral corner of the marginal/postmarginal plates (Long, 1995, p. 54, fig.7). This triangular fossa is similar in position and morphology to the anterior fossa described here whereas the other surfaces resemble the lateral and medial fossae. Long (1995, pp. 53–55) explicitly considered this triangular fossa to represent the origin of the *m. levator palatoquadrati* and suggested at least some of the remaining space represented the origins for an opercular muscle or *m. levator hyomandibulae* (Long, 1995, considered the element attaching to the submarginal plate to be an opercular cartilage or epihyal), with the former assumedly originating on the lateral space and the latter the medial one.

The lateral consolidated region shows significant variation within eubrachythoracid arthrodires. Compared to morphologically plesiomorphic coccosteomorphs and *E. calliaspis* (Dennis‐Bryan, 1987, figs. 9 and 11), this region is much more posteriorly extensive in large, macropredatory Cleveland Shale arthrodires *Dunkleosteus* (File S3, Dunkle & Bungart, 1946), *Gorgonichthys* (Dunkle & Bungart, 1940, 1946), and *Heintzichthys* (Dunkle & Bungart, 1946; CMNH 5648), almost reaching the posterolateral margins of the skull roof. This may be related to the more posteriorly extensive cheek unit and mouth in these taxa (Engelman, 2023b). Notably, in non‐macropredatory aspinothoracidans which have a long cheek unit but a smaller adductor region and mouth, such as *Bullerichthys* (Dennis & Miles, 1980, fig. 6) and several selenosteids (Rücklin et al., 2015, fig. 2; Stensiö, 1963, fig. 113a–c), the lateral consolidated region is much less extensive and does not reach the posterior margin of the skull roof. There is also significant variation in the shape of the lateral consolidated region within Cleveland Shale arthrodires (Dunkle & Bungart, 1946, fig. 4): the region in *Gorgonichthys* is extremely narrow, in *Heintzichthys* it is elongate and almost sub‐rectangular in shape, whereas in *Dunkleosteus* it is triangular and much more medially extensive than in these other taxa. Indeed, the lateral consolidated region of *Dunkleosteus* is significantly larger than in most other arthrodires (File S9), occupying 17%–18% of the total surface area of the ventral skull roof in large individuals versus <9% in most taxa. The only arthrodire examined that approaches *Dunkleosteus* in relative lateral consolidated region size is *W. uwagedensis* (Long, 1987). By contrast, arthrodires with highly integrated cheek plates and skull roofs like camuropiscids and selenosteids have proportionally smaller lateral consolidated regions (Dennis & Miles, 1979a; Rücklin et al., 2015; Stensiö, 1963). Dunkle and Bungart (1946) suggested the variation in lateral consolidated region size and morphology reflected functional differences between arthrodires; we agree with this conclusion but disagree with their interpretation that this variation relates to differences in adductor muscle anatomy, as the identity of the muscles originating on the lateral consolidated region is unclear. Previous studies considered this region as the origin of the *m. adductor mandibulae* (Anderson & Westneat, 2007, 2009; Dunkle & Bungart, 1946, p. 8; Miles, 1969, p. 137; Stensiö, 1963). However, an anatomical reasoning was never provided for this interpretation besides noting the lateral consolidated region represented a clear, large attachment site for muscle. Such an interpretation also overlooks the adductor fossa formed by the palatoquadrate when this cartilage is preserved in arthrodires (Brazeau et al., 2023; Dennis & Miles, 1979b; Gardiner & Miles, 1990; Hu et al., 2017; Miles & White, 1971; Young et al., 2001).

By comparison, Long (1995) noted if the *m. adductor mandibulae* of arthrodires attached to the lateral consolidated region, its path would be obstructed by the palatoquadrate. Instead, he suggested this fossa represented an origin for the *m. levator palatoquadrati*. This muscle attaches posterior to the orbit in sharks (De Iuliis & Pulerà, 2011; Dearden et al., 2021), roughly in the same area as the lateral consolidated region. This interpretation is supported by the discovery of a postorbital muscle in this location in Gogo Formation arthrodires (WAM 12.8.1; Trinajstic et al., 2013, fig. 3k,l), interpreted as representing the *m. levator palatoquadrati* (= *m. levator arcus palatini* in that study; this muscle is considered homologous with the *m. levator palatoquadrati*; Anderson, 2008a).

The tripartite division of the lateral consolidated region in *Dunkleosteus* suggests multiple muscles attached in this region, though the possibility of multiple divisions of a single muscle body cannot be ruled out. The anterior fossa likely corresponds to the origin of the *m. levator palatoquadrati*, given this is the location of the origin of this muscle in the Gogo Formation arthrodires (Trinajstic et al., 2013), as well as a similar postorbital origin for this muscle in many fishes (Datovo & Johnson, 2025). The large anterior fossa of *Dunkleosteus* suggests the *m. levator palatoquadrati* was proportionally larger in *Dunkleosteus* than either in the Gogo Formation arthrodires or chondrichthyans (Motta & Wilga, 1995; Motta & Wilga, 1999), in which these muscles are relatively small. The muscles attaching to the lateral and medial fossae are more difficult to determine. Given its position immediately dorsal to the submarginal plate (and thus the hyomandibula), the elongate lateral fossae could serve as the origin of the *m. levator hyomandibulae*. However, the lateral fossa seems to extend significantly further anteriorly than the submarginal plate (Figure 9), which would argue against this being the case. Alternatively, given the anterior end of the medial fossa is located near the anterior postorbital process of the neurocranium, which is considered the articulation between the hyomandibula and neurocranium (see “Submarginal plate and hyomandibula”), the medial fossa could represent the origin of the *m. levator hyomandibulae*. However, much of the contact with the hyomandibula on the submarginal plate is ventral to the lateral fossa, not the medial one. None of the fossae are well positioned to represent a branch of the *m. adductor mandibulae*. Of the three fossae, the anterior would be best suited to represent an origin of this muscle in terms of its position relative to the adductor chamber, shape, and size, but this fossa also has the strongest evidence suggesting a different muscle (the *m. levator palatoquadrati*) originated here.

The functions of the muscles on the lateral consolidated region are also unclear. In chondrichthyans, the *m. levator palatoquadrati* is very small and primarily serves to stabilize and retract the palatoquadrate (Dearden et al., 2021; Motta & Wilga, 1995, 1999). In most arthrodires, the lateral consolidated region is moderately sized relative to the area of the entire head shield (File S9), though the actual muscle body of the *m. levator palatoquadrati* remains small (Trinajstic et al., 2013). In *Dunkleosteus*, the lateral consolidated region is much more extensive, and the anterior fossa, the likely origin of the *m. levator palatoquadrati*, is also very large, suggesting this muscle and other structures attaching to the lateral consolidated region were hypertrophied in *Dunkleosteus*.

These features, if considered by themselves, would imply a more mobile cheek unit and mandibular arch for *D. terrelli*. However, this is at odds with all arthrodires, including *D. terrelli*, exhibiting functional holostyly, with the cheek unit immobile relative to the skull roof and neurocranium (see “Submarginal plate and hyomandibula,” below). This is not true holostyly via fusion of the palatoquadrate and neurocranium as seen in chimaeroids, but is functionally equivalent due to the overlying dermal plates being united by immobile sutures. The dermal cheek unit and skull roof of *Dunkleosteus* have only one true sutural connection between the postnasal plate and preorbital, but this connection is sufficiently complex to prevent any motion of the upper jaw relative to the neurocranium.

Long (1995) proposed the *m. levator palatoquadrati* of arthrodires served to ventilate the gill chamber by protraction and retraction of the cheek unit in combination with antagonistic actions of the *m. preorbitalis*. This interpretation is supported by the fact that all arthrodires with weakly developed lateral consolidated regions and highly integrated skull roofs and cheek units have been interpreted as fast‐swimming nektonic species (Jobbins et al., 2022; Trinajstic, Briggs, & Long, 2022) which could in turn relate to a shift from active buccal pumping to ram ventilation as the primary means of oxygenation (Miles, 1967; J. Long personal communication, 2023). However, this is at odds with the apparent immobility of the cheek plate in all eubrachythoracids (see “Submarginal plate and hyomandibula,” below).

It is possible the *m. levator palatoquadrati* primarily served to stabilize the upper jaw, which is supported by the large lateral consolidated region and poorly connected posterior cheek of *Dunkleosteus*, but it is unclear why muscles would be needed to support and stabilize this region if the mandibular arch is already firmly attached to the neurocranium by bony and ligamentous connections. This is supported by the apparent loss of the *m. levator palatoquadrati* in chimaeroids, which have a fully holostylic jaw attachment and thus no need for muscular stabilization (Anderson, 2008a; Dearden et al., 2021; R. Dearden personal communication 2024; Diogo et al., 2018; Ziermann et al., 2014, p. 23). Similar issues would be present with any interpretation of the lateral or medial fossa as the attachment sites of the *m. levator hyomandibulae*. It is also unclear why such a specialized arrangement would develop in *Dunkleosteus,* given the hypertrophied lateral consolidated region and loosely connected posterior cheek do not seem to provide any functional benefits over a coccosteomorph‐like arrangement. Even if the muscles on the lateral consolidated region served in cheek stabilization, a simpler, stabler, and evolutionarily more cost‐effective solution can be achieved by simply suturing or otherwise integrating the cheek unit, submarginal plate, and skull roof into a single immobile structure, as in camuropiscids, brachydeirids, and selenosteids. There is a great deal of variation in lateral consolidated region morphology in arthrodires, which seems to correlate well with the degree of integration of the posterior cheek, but given the cheek plate apparently remained universally immobile in arthrodires it is unclear what this interspecific variation means.

#### 3.3.1 Potential alternative arrangements

An alternative arrangement to consider is that previous authors (Anderson & Westneat, 2007, 2009; Dunkle & Bungart, 1946; Miles, 1969; Stensiö, 1963) might have been partially correct and at least a branch of the *m. adductor mandibulae* originated on the lateral consolidated region (Figure 6c), specifically the anterior fossa. In this scenario, the *m. adductor mandibulae* would be divided into multiple branches: a superficial division (*m. adductor mandibulae pars superficialis*) originating within the adductor fossa of a relatively narrow palatoquadrate and inserting on the adductor fossa formed by the intermediate Meckelian cartilage, and a deep division (*m. adductor mandibulae pars profundus*) originating on the anterior fossa of the lateral consolidated region and inserting into the dorsal region of the adductor lamina not covered by cartilage. The *m. adductor mandibulae pars profundus* would contract medial to the palatoquadrate, resolving the issue of how the *m. adductor mandibulae* could insert on the skull roof without intersecting the palatoquadrate. Under this model, the medial fossa would represent the origin of the *m. levator hyomandibulae*, with the hyomandibula spanning the region between this element's attachments on the anterior postorbital process of the neurocranium and the submarginal plate (see “Submarginal plate and hyomandibula”). The lateral fossa would then represent the origin of the *m. levator palatoquadrati*, as this fossa is immediately dorsal to the less medially extensive palatoquadrate. This is somewhat reminiscent of the recent proposal by Datovo and Johnson (2025) that a *pars sphenoidalis* of the *m. adductor mandibulae* inserting on the neurocranium is a synapomorphy of eugnathostomes (secondarily lost independently in holocephalians and elasmobranchs). Datovo and Johnson (2025) propose such an arrangement was absent in arthrodires, but given the adductor fossa in these taxa was largely open medially, a scenario could be envisioned where the medialmost fibers of the *m. adductor mandibulae* attach to the skull roof.

This arrangement would provide several advantages over the model proposed here. First, it allows for more verticalized contractile paths for those fibers of the *m. adductor mandibulae* attaching both to the intermediate Meckelian cartilage as well as those attaching to the dorsally exposed region of the infragnathal (Figure 6c). Additionally, it provides a more stable origin and higher pullout strength for the fibers inserting into the exposed dorsal portion of the adductor lamina of the infragnathal because both the insertion and origin of these fibers are on a bony enthesis rather than cartilage (Huber et al., 2009; Liem & Summers, 1999). If this were not the case, the contractile strength of these fibers would be limited by the lower pullout strength of their origin on the cartilaginous palatoquadrate, negating most of the advantages of a bony enthesis on the infragnathal. It is also supported by the ridge on the articular cartilage, potentially implying a separation between the region of the *m. adductor mandibulae* inserting on the Meckel's cartilage and the portion inserting on the infragnathal.

However, there are several anatomical issues with such a model. Perhaps most obviously, it contradicts direct observations of muscle bodies in the Gogo Formation arthrodires that show a *m. levator palatoquadrati* originating just posterior to the orbit (Trinajstic et al., 2013), in a region roughly equivalent to the anterior fossa in *Dunkleosteus terrelli*. Similarly, because of the location of the submarginal plate and its contact for the hyomandibula, fibers of the *m. levator palatoquadrati* originating on the posterior half of the lateral fossa would intersect with the hyomandibula if they inserted onto the palatoquadrate. If the *m. levator palatoquadrati* did not utilize the entire lateral fossa to attach to the posterior palatoquadrate, its ability to stabilize the enlarged mandibular arch and loosely consolidated posterior cheek (see “Submarginal plate and hyomandibula,” below) of *D. terrelli* would be limited, especially in the region immediately dorsal to the mandibular articulation.

Such an arrangement also might require a hypothetical *m. adductor mandibulae pars profundus* to stretch beyond physiologically tolerable limits (110%–125% resting length) during jaw opening, as noted by previous studies which assumed the entire lateral consolidated region served as an origin for the *m. adductor mandibulae* (Anderson, 2010; Anderson & Westneat, 2009). At a gape of 22°, this hypothetical muscle has already reached its maximum stretch (120% of resting length; measured as the distance between the lateral consolidated region and the boundary between the infragnathal and intermediate Meckelian cartilage in lateral view), yet there is almost no clearance between the upper and lower odontoids (see “Gape of *Dunkleosteus*”). Gape angles achieving sufficient clearance for food to pass between the odontoids (>45°) require biomechanically impossible muscle stretch factors of 140% or 150% resting length, respectively. This further argues against any part of the *m. adductor mandibulae* originating on the lateral consolidated region, because a muscle spanning these landmarks could not stretch enough to allow the animal to open its mouth.

Another alternative is if the articulation on the submarginal plate represents a contact for an epihyal, rather than a hyomandibula, as has been proposed by some authors (Long, 1995; Young, 1986). In this scenario, the lateral fossa would serve as an origin for an opercular muscle, whereas the medial fossa would serve as the origin for the *m. levator hyomandibulae*. Much like the previous model, the hyomandibula would be free to follow the path of the medial fossa to its contact with the braincase on the anterior postorbital process of the neurocranium. However, this interpretation seems at odds with specimens showing a hyomandibula attaching to the internal face of the submarginal (Miles & White, 1971). Similarly, if the submarginal represents an opercular cartilage, why would the submarginal of *Dunkleosteus* be rod‐like and have an extensive contact for the underlying cartilage, or be separate yet tightly integrated with the cheek unit in other arthrodires rather than lost (see below)? Young (2010, pp. 531–532) suggests this represents a novel system where the mandibular arch was primarily suspended by a modified opercular cartilage, but if that is the case, why would a significant area of attachment for a *m. levator hyomandibulae* also be present?

As a result, neither of these alternative models is favored here. However, we note them as potential if unlikely possibilities in the hopes that it might better help future authors understand the musculature in this region.

### 3.4 Functional group 4: Submarginal plate and hyomandibula

One plate that has not been previously discussed in *Dunkleosteus* is the submarginal plate (Figure 2). This plate was not described by Heintz (1932) nor considered in subsequent analyses of *Dunkleosteus* (Anderson & Westneat, 2009; Heintz, 1932; Stensiö, 1963), though it was figured without discussion by Dunkle and Bungart (1946) and mentioned in passing by (Miles, 1969, p. 141). In most eubrachythoracid arthrodires, the submarginal plate is a flattened, rectangular element attached to the back of the cheek unit. The submarginal plate is especially important in understanding the oropharyngeal anatomy of arthrodires because it articulated with the hyomandibula internally (Carr et al., 2009; Gardiner & Miles, 1990; Hu et al., 2017; Miles & White, 1971) and is often the only evidence constraining the position of the hyomandibula due to this cartilage rarely preserving. Ancestrally, the submarginal plate is fused to the suborbital/postorbital plates ventrally with an overlap facet on the marginal plate of the skull roof dorsally (File S3A,B), though the latter are not fused (Miles & Westoll, 1968). In some groups such as camuropiscids, brachydeirids, and selenosteids, the submarginal plate can be larger, have an irregular or disc‐like shape, and is either fused to the skull roof and cheek plates or extensively interdigitates with these elements (Dennis & Miles, 1979a, 1979b; Gross, 1932; Rücklin, 2011; Young, 2010), consolidating the cranium into a simple box (File S3E,F).

**FIGURE 14:**
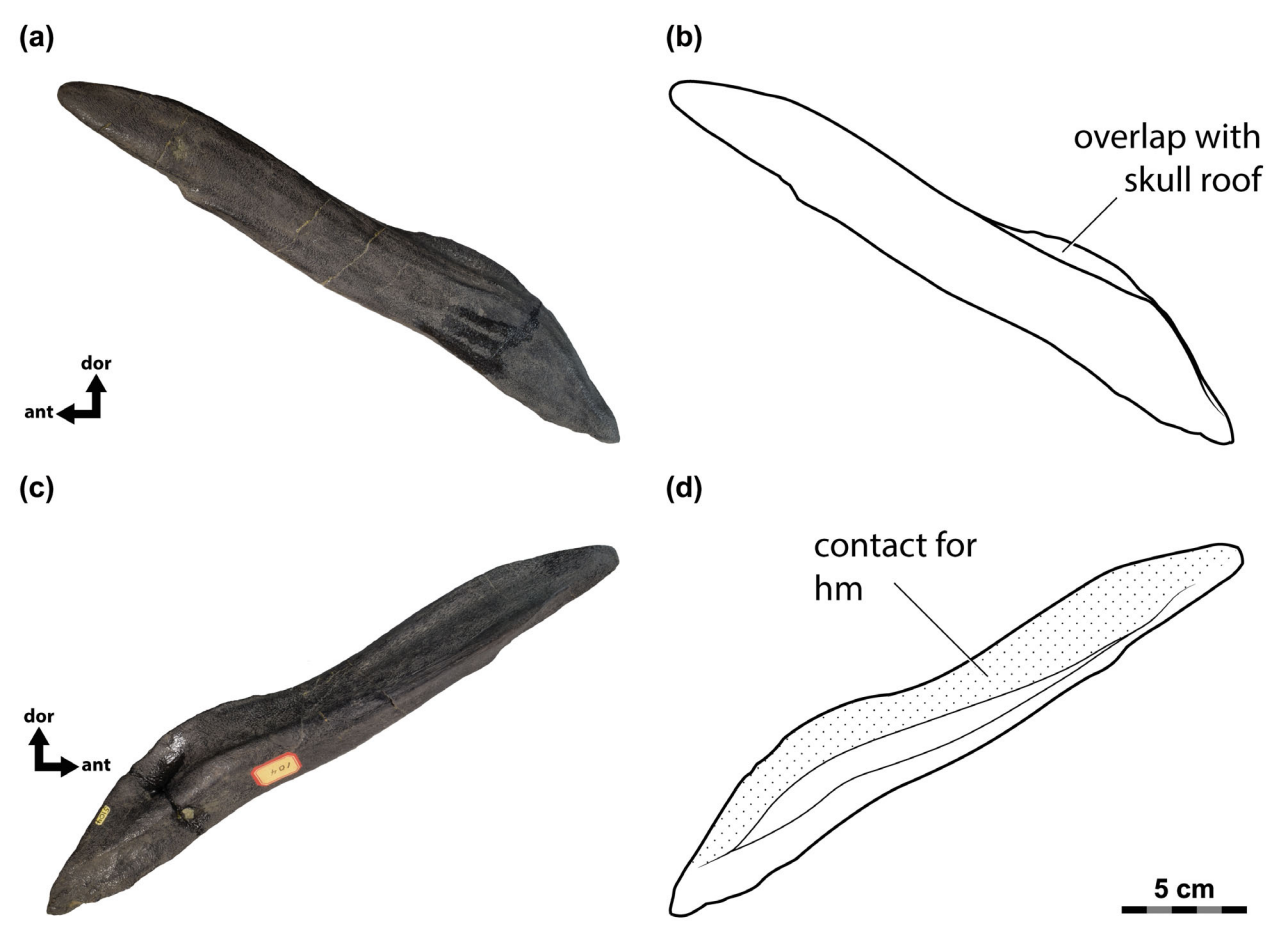
Photographs (a, c) and line drawings (b, d) left submarginal plate of *Dunkleosteus terrelli* (CMNH 5104) in external (a–b) and internal (c–d) view. Abbreviations follow Figure 2.

The submarginal plate of *Dunkleosteus* is unlike any other arthrodire. It is a long, rod‐like element (Figure 14, Files S3D and S10, Table 2), both extremely slender relative to its length (greatest length is approximately 8–9× width in most specimens) and relatively long compared to the anteroposterior length of the head (approximately 40%–50% anteroposterior head length). By contrast, in other eubrachythoracids the submarginal is longer than tall but is still sub‐rectangular in shape (length ≤7× width; Table 2, File S3) and proportionally shorter (approximately 15%–35% head length). Larger specimens of *D. terrelli* have proportionally longer submarginal plates, agreeing with the general positive allometry in mouth and cheek unit size in this taxon (Engelman, 2023b; Heintz, 1932). The submarginal plate of *Dunkleosteus* is also substantially less consolidated with the rest of the skull than in other arthrodires (File S3D). In contrast to other arthrodires, the submarginal plate lacks sutures with either the head shield or cheek unit and its contact faces on these elements are barely present, if at all, making the edges of this plate rounded in cross‐section rather than having a tongue and groove overlap (Figure 6, File S10H,I). In some specimens a very small overlap facet for the skull roof can be seen on the submarginal plate (Figure 14b), but even this can be variable. For example, in CMNH 6194 an overlap facet for the skull roof appears to be present on the left submarginal plate but absent on the right (File S10C,D). The poor development of overlapping contacts means that the submarginal plate and the posterior skull roof and bony cheek were almost completely separated from one another, only being connected by articulations at the anterior end of the skull and underlying cartilaginous elements.

**TABLE 2:** Proportions of submarginal plates in select brachythoracid arthrodires. Length refers to greatest length, with width being perpendicular to this. “Ratio” refers to the length of the submarginal plate as a proportion of total head (skull roof) length.

Taxon	Specimen	Length	Width	*L*/*W*	Head length	Ratio	References
*Holonema westolli*	—	2.07	3.68	0.56	9.64	0.21	Miles and White (1971), figs. 2 and 4
*Stenosteus angustopectus*	—	4.28	2.74	1.56	16.03	0.27	Carr (1996), fig. 1
*Gymnotrachelus hydei*	—	1.34	0.85	1.58	12.53	0.11	Carr (1994), fig. 2
*Latocamurus coulthardi*	WAM 86.9.699	0.65	0.31	2.96	4.60	0.14	Present study
*Harrytoombsia elegans*	WAM 70.4.254	1.70	0.70	2.43	6.18	0.28	Miles and Dennis (1979), fig. 4
*Buchanosteidae* sp.	ANU V244	2.19	0.83	2.63	5.19	0.42	Hu et al. (2017), figs. 6
*Millerosteus minor*	LDUCZ‐V998	0.77	0.27	2.85	2.96	0.26	Desmond (1974), fig. 10
*Titanichthys cf. T. clarki*	CMNH 50319	17.75	5.84	3.04	49.35	0.36	Boyle and Ryan (2017), fig. 2
*Coccosteus cuspidatus*	ROM VP 52664	2.56	0.80	3.20	7.72	0.33	Present study
*Plourdosteus canadensis*	MNHM 2–571	1.64	0.44	3.73	8.66	0.19	Vézina (1988), fig. 11
*Heintzichthys gouldii*	CMNH 9407	12.38	3.32	3.73	—	—	Carr (1991), fig. 5
*Mcnamaraspis kaprios*	WAM 89.9.676	1.84	0.45	4.06	5.63	0.33	Long (1995), pl.1.2
*Torosteus pulchellus*	WAM 91.4.33	2.10	0.55	3.80	6	0.35	Present study
*T. pulchellus*	WAM 91.4.32	1.50	0.50	3.00	5.41	0.28	Present study
*T. pulchellus*	WAM 88.2.7	2.20	0.45	4.89	6.65	0.33	Present study
*Torosteus tuberculatus*	—	2.53	0.58	4.36	7.02	0.36	Gardiner and Miles (1990) fig. 2
*Incisoscutum ritchei*	NHMUK VP P50923	1.28	0.29	4.41	6.121	0.21	Dennis and Miles (1981), fig. 2
*Heintzichthys gouldii*	—	7.01	1.34	5.23	26.1	0.27	Carr (1991), fig. 2
*Compagopiscis croucheri*	WAM 70.4.263	1.69	0.28	6.04	5.44	0.31	Trinajstic and Hazelton (2007), fig. 2
*C. croucheri*	WAM 95.1.3	1.51	0.36	4.19	5.49	0.27	Present study
*C. croucheri*	WAM 86.9.673	1.65	0.31	5.32	6.15	0.27	Present study
*Eastmanosteus calliaspis*	—	5.91	0.94	6.29	15.83	0.37	Dennis‐Bryan (1987), fig. 5
*Coccosteus cuspidatus*	—	2.46	0.39	6.31	7.08	0.35	Miles and Westoll (1968)
*Dunkleosteus terrelli*	CMNH 5104	26.51	4.06	6.53	—	—	Present study
*Parabelosteus* sp.	—	3.41	0.45	7.58	8.6	0.40	Rücklin (2011), fig. 18
*D. terrelli*	CMNH 6090	18.40	2.35	7.83	51.00	0.36	Present study
*D. terrelli*	CMNH 6194 (right)	11.20	1.42	7.89	29.00	0.39	Present study
*D. terrelli*	CMNH 7054	25.51	3.19	8.00	57.00	0.45	Present study
*D. terrelli*	CMNH 6194 (left)	11.55	1.44	8.02	29.00	0.40	Present study
*D. terrelli*	CMNH 5768	29.80	3.71	8.03	61.30	0.49	Present study
*D. terrelli*	CMNH 7424	13.74	1.38	9.96	34.00	0.40	Present study

Abbreviations: ANU, Australian National University, Canberra, Australia; CMNH, Cleveland Museum of Natural History, Cleveland, USA; LDUCZ, Grant Museum of Zoology, University College, London, UK; NHMUK, The Natural History Museum (London), London, UK; ROM, Royal Ontario Museum, Toronto, Canada; WAM, Western Australian Museum, Perth, Australia.

*E. calliaspis* shows an intermediate condition between the ancestral state seen in coccosteomorphs and the extremely loosely consolidated posterior cheek of *Dunkleosteus* (Files S3C and S11). Aside from *Parabelosteus* (see Denison, 1978, fig. 74a), *E. calliaspis* most closely resembles *Dunkleosteus* in its submarginal plate morphology (Table 2). Additionally, in *E. calliaspis* the contact facet between the submarginal and head shield is very small and the submarginal is less incorporated into the cheek unit both in terms of the length of its anteroposterior contact and the size of the contact face between it and the suborbital/postsuborbital plates (File S3; Dennis‐Bryan, 1987, p. 21). The intermediate state of these characters suggests the unusual morphology of *Dunkleosteus* may represent an extreme within a broader evolutionary trend toward a looser posterior cheek in dunkleosteoids.

Despite this, the submarginal plate of *Dunkleosteus* was not vestigial, as it still retains a complex morphology. The internal face of this plate has a prominent longitudinal ridge spanning most of its length that makes the submarginal triangular in cross‐section (File S10H,I). This ridge is present on the submarginal of other eubrachythoracids (Carr, 1991; Gardiner & Miles, 1990) and represents a site for the hyomandibula to attach dorsally (Miles & White, 1971, fig. 111). This suggests the hyomandibula of *Dunkleosteus* was a rod‐like element spanning most of the length of the posterior cheek. The bar‐like shape of the submarginal suggests it was unlikely to have served as a support for a functional operculum, as has been suggested for the submarginal of other arthrodires (Hu et al., 2017; Young, 1979), as its shape and extensive attachment with the hyomandibula mean it could not oscillate to induce water flow over the gills.

It is unclear if the hyomandibula had a more distal articulation for the ceratohyal. The extensive ridge for its attachment on the submarginal indicates the hyomandibula extended to the distal end of this plate, which might imply it articulated with the ceratohyal, but it is also possible the ceratohyal only articulated with the articular cartilage, as proposed by Goujet (1984). The articular cartilage of *Dunkleosteus* shows a clear attachment for the mandibulohyoid ligament (Figure 3f–g), contra Johanson (2003), suggesting a ceratohyal‐mandible connection was present, at the very least.

Despite these specializations for a more loosely connected posterior cheek, the cheek unit of *Dunkleosteus* was still immobile relative to the head shield. The postnasal plate dorsally articulates with the skull roof via a complex suture (Figure 11), which would prevent kinesis. As mentioned under “Suborbital groove and *m. preorbitalis*,” the elements composing the cheek unit (including the postnasal plate) were immobile relative to one another, indicating the rest of the cheek plate could not move relative to the postnasal and its firm connection to the head shield. In *Eastmanosteus*, the suborbital plate also forms a tight suture with the preorbital plate of the head shield, though in *Dunkleosteus* this contact is substantially looser, with the plates in physical contact but lacking a suture.

Additionally, there is a postorbital contact facet between the skull roof and the cheek unit (Figure 13). In flattened Cleveland Shale specimens, this is a laterally open facet positioned directly lateral to the posterior supraorbital crista but would have been opened ventrally if the skull roof was preserved three‐dimensionally. This structure appears to reflect a physical tongue and groove overlap between elements rather than a ligamentous or sutural connection. A corresponding overlap facet is not seen on the external or internal face of the suborbital plate (Figure 7d), suggesting it might have received a dorsally projecting process of the palatoquadrate. This is supported by three‐dimensionally preserved specimens of *Dunkleosteus marsaisi* from Morocco (GPIT/PD/9; Rücklin & Clément, 2017, fig. 5), which show no overlap between the skull roof and cheek unit. This connection in *Dunkleosteus* resembles the postorbital connection between the palatoquadrate and neurocranium in hexanchiids, which biomechanical studies have found prevent any protraction or retraction of the upper jaw (Kryukova & Kuznetsov, 2020). The cheek unit of *Dunkleosteus* is less integrated to the rest of the cranium than in any other arthrodire, but despite this, the cheek apparently remained immobile relative to the skull roof. Given *Dunkleosteus* shows one of the least integrated cheek units/head shields among all of Arthrodira, it is unlikely other members of this group with more integrated skull roofs and cheek units showed significant mobility of the cheek.

The functional significance of the loosely connected cheek of *Dunkleosteus* is unclear. It clearly represents an apomorphic specialization of dunkleosteoids relative to the outgroup, given *E. calliaspis* (an early‐diverging dunkleosteoid) and *D. terrelli* (a late‐diverging one) show progressively more extreme versions of this condition relative to the ancestral state (as seen in coccosteomorphs) (File S11). It cannot be reflective of a vestigial submarginal plate, as the submarginal plate is still large in *D. terrelli* and its extensive contact for the hyomandibula suggests the latter element was still fairly large. Similarly, the anterior cheek, while still incapable of kinesis, appears to have been more loosely attached than other arthrodires. The loosely consolidated posterior cheek also cannot be related to a reduction of the submarginal to stabilize the skull, as *Dunkleosteus* shows the opposite morphological trend from arthrodire taxa with a highly integrated skull such as camuropiscids and selenosteids (File S11), and indeed the condition in *D. terrelli* results in a less stable posterior skull. It does not seem to be a side effect of an elongate cheek unit, as *Titanichthys* has an elongate cheek but retains an almost coccosteomorph‐like submarginal plate morphology (Boyle & Ryan, 2017). Finally, it cannot represent a “spandrel” arising from historical constraint because the cheek morphology seen in *Dunkleosteus* imposes significant additional costs in fitness on the animal beyond the plesiomorphic condition. The lack of sutures or firm articulations between the skull roof/submarginal plate and submarginal plate/cheek unit means the mandibular arch of *Dunkleosteus* is poorly stabilized at its posterior end, making it highly vulnerable to dislocation by perpendicular or torsional forces from struggling prey. While other elements likely played a role in stabilizing the posterior cheek (e.g., the hyomandibula, the ceratohyal, or even a modified opercular cartilage as suggested by Young, 2010), the mandibular arch of *Dunkleosteus* is far less securely attached to the neurocranium/head shield than in any other arthrodire. This suggests the specialized cheek morphology of *Dunkleosteus* reflects a functional adaptation with the fitness benefits of this feature outweighing the functional costs of a less stable posterior cheek, even if the benefits remain unknown.

A related issue is the connection of the hyomandibula to the neurocranium. At the anteromedial edge of the lateral consolidated region, there is a distinct rugosity (Figure 13, ant. postor. proc. neurc.). In other arthrodires, this rugosity has been interpreted as associated with the anterior postorbital process of the neurocranium, which is the site of the articulation between the neurocranium and hyomandibula (Goujet, 1984; Miles & White, 1971). Given the locations of the anterior (anterior postorbital process) and posterior (visceral face of the SM) articulations for the hyomandibula, it is very difficult for a rod‐like element to maintain contact with both of these articulations and still retain a rod‐like shape. Additionally, this element would have to pass through much of the lateral consolidated region, limiting possible insertion sites for any muscle originating on this surface. This is also one reason why the lateral consolidated region cannot represent an origin for the *m. adductor mandibulae*. If the lateral consolidated region or any combination of its fossae served as an attachment for the *m. adductor mandibulae*, its connective path would pass through the hyomandibula and prevent this element from articulating with the neurocranium. The only possible exception would be if the anterior fossa alone housed a branch of the *m. adductor mandibulae*, but that is the structure that other arthrodire fossils suggest is best correlated with the *m. levator palatoquadrati*.

### 3.5 Functional group 5: Head‐trunk musculature (*m. cucullaris*, *m. levator capitis major*, and *m. levator capitis minor*)

The musculature at the head‐trunk interface in arthrodires is also involved in cranial kinematics due to their role in raising and depressing the cranium (Anderson & Westneat, 2009; Heintz, 1932; Miles, 1967). These muscles are of particular importance in arthrodires as the prominent cranio‐thoracic joint makes motion between the head and trunk armor highly integrated (Miles, 1967). The functional role of these muscles in *Dunkleosteus* has been discussed in several prior studies (Anderson & Westneat, 2007, 2009; Heintz, 1932). Previous studies (Adams, 1919; Heintz, 1932) described these muscles based on presumed position and function, remaining agnostic to their homology. More recent studies of arthrodires suggest the jaw adductors/head depressors (*m. depressor capitis*) and jaw extensors/head elevators (*m. levator capitis*) are homologous to the *m. cucullaris* (Trinajstic et al., 2013) and *m. epaxialis* (Anderson & Westneat, 2007, 2009) of other vertebrates, respectively. We follow previous studies (Trinajstic et al., 2013) in preferring the term *m. cucullaris* over *m. depressor capitis* but retain the term *m. levator capitis* as in arthrodires the anterior epaxial musculature is divided into multiple muscles (*m. levator capitis major* and *minor*).

**FIGURE 15:**
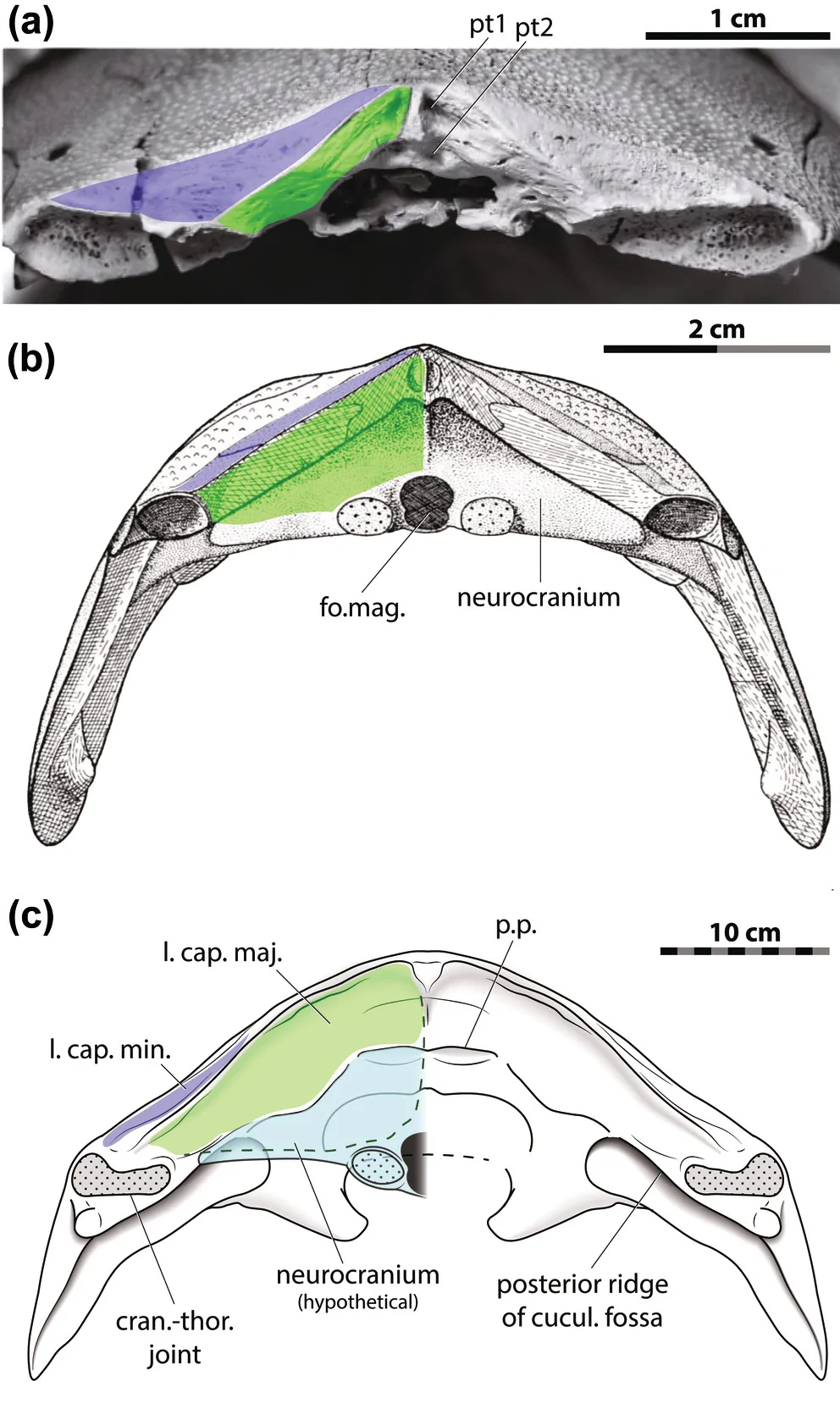
Skull roofs of *Buchanosteus confertituberculatus* (a, NMV P159895, modified from Long et al., 2014, fig. 2e), *Coccosteus cuspidatus* (b, modified from Miles & Westoll, 1968, fig. 24), and *Dunkleosteus terrelli* (c, CMNH 6090) in posterior view, showing the attachment sites for the *m. levator capitis major* and *minor* relative to the cranio‐thoracic joint. Muscle attachment sites for (a) follow Long et al. (2014). For (c), neurocranial shape is hypothetical based on anatomical landmarks for its contact with the skull roof in *D. terrelli* and general morphological patterns in other arthrodires (Goujet, 1984; Heintz, 1932; Long et al., 2014; Miles & Westoll, 1968; Stensiö, 1963). Green dotted line in (c) represents approximate attachment area for *levator capitis major* when neurocranium is restored. Fo.mag., foramen magnum, pt1/2, paired pits discussed in Long et al. (2014), other abbreviations follow Figure 2.

The posterior skull roof of *D. terrelli* is thick, up to 9 cm in very large specimens (CMNH 5768), compared to 2–3 cm thick across most other regions of the armor like more anteriorly on the skull roof at the level of the orbits or across the suborbital, median dorsal, anterior dorsolateral, and adductor lamina of the infragnathal plates (Figure 15). The posterior skull roof can be divided into two regions, separated by the posterior edge of the head shield and distinguishable by their position relative to the cranio‐thoracic joint (Figure 15). The first of these regions is a large, well‐defined fossa located dorsomedial to the cranio‐thoracic joint and spanning most of the head shield, being divided at the midline by the posterior process of the nuchal and a crest descending from this process (Figure 15, green). The second is a much smaller, paired triangular fossa positioned immediately dorsal to the cranio‐thoracic joint and oriented posteroexternally (Figure 15, blue). Despite facing slightly externally this structure is not continuous with the external face of the dermal armor and as in other arthrodires lacks dermal ornamentation (Miles & Westoll, 1968), indicating it was covered by soft tissue in life. A similar division of the nuchal space has been observed in a number of other arthrodires (Long et al., 2014; Miles & Westoll, 1968; Stensiö, 1963, p. 194). The first structure represents a prominent surface for muscle attachment, the second may also be, but this is less clear.

Stensiö (1963) called these two structures the “lateral and medial divisions of the nuchal thickening” whereas Miles and Westoll (1968) called them the “posterior ascending/descending lamina.” We prefer to use the former over the latter, not only because the terminology of Stensiö (1963) more clearly describes the morphology, but the terminology of Miles and Westoll (1968) appears to be based on a misreading of Stensiö (1959). Miles and Westoll (1968) claimed their use of their term came from Stensiö (1959, fig. 31), but this figure appears to be using the term “posterior, descending lamina of the nuchal plate” to refer to the crest separating the lateral and medial divisions of the nuchal thickening (as in, a descending lamina on the posterior face of the nuchal plate) rather than the areas it subdivides (there is no mention of an ascending lamina).

#### 3.5.1 m. levator capitis major

In placoderms, the *m. levator capitis major* has been identified as attaching to the posterior face of the cranium, as its homolog, the *m. epaxialis*, does in other vertebrates (Dutel et al., 2015; Motta & Wilga, 1995; Staggl et al., 2022). This muscle either inserts into the posterior face of the cartilaginous endocranium in non‐arthrodiran placoderms (Castiello et al., 2020, fig. 9) or, in arthrodires, a combination of the thickened posterior face of the nuchal plate and the endocranium (Long et al., 2014; Miles, 1967; Miles & Westoll, 1968; Stensiö, 1963). Stensiö (1963, p. 194, 221, 289–291, figs. 95 and 108) suggested in eubrachythoracids the posterior face of the endocranium and the posterior side of the nuchal thickening (*th.n*. in that study) combined to form a subnuchal space that serves as the insertion of the *m. levator capitis major*. Miles and Westoll (1968, p. 458) consider the *m. levator capitis major* of *Coccosteus* to attach to the subnuchal space on the posteroventral surface of the cranium, formed by the medial division of the posterior thickening of the nuchal plate (posterior ascending lamina of these authors) and the posterodorsal wall of the occipital region of the neurocranium (Figure 15b). Miles (1969, p. 149) further considered the *m. levator capitis* to attach here in arthrodires more generally. White and Toombs (1972, p. 395) identified small depressions on the posterior face of the nuchal plate for this muscle in the buchanosteid arthrodire *Buchanosteus* (Figure 15a, pt1). Long et al. (2014) modified this to suggest the *m. levator capitis major* attached to a much larger region on the posterior face of the nuchal plate (Figure 15a; see Long et al., 2014, fig. 2), including the area of the pits identified by White and Toombs (1972). The *m. levator capitis minor* was identified as attaching to a region lateral to the *m. levator capitis major*, still on the nuchal plate (Castiello et al., 2020; Long et al., 2014). By comparison, Trinajstic et al. (2013) reported preserved cranio‐thoracic muscles (*levator capitis major* and *minor*) in Gogo Formation arthrodires of which the *m. levator capitis major* of *Compagopiscis croucheri*, *Eastmanosteus calliaspis*, and *Incisoscutum ritchei* was reconstructed as extending underneath and significantly anterior to the posterior margin of the head shield (Trinajstic et al., 2013, fig. 1b,d).

A more internal attachment for the *m. levator capitis major/minor* would not have been possible in *Dunkleosteus* based on the location of the paired pits on the visceral face of the nuchal, at the posterior margin of the embayment of the skull roof (Figures 2, 9, and 15c). The neurocranium itself would have extended posteriorly beyond this contact, with the foramen magnum at the level of the cranio‐thoracic joint (Carr et al., 2009; Heintz, 1932). These pits are often interpreted as housing the occipital endocranial process (Goujet, 1984; Miles & Westoll, 1968; Rücklin, 2011), though Stensiö (1963, p. 289) and Young (2004) interpreted them as housing the levator muscles. In many eubrachythoracids these pits face anteroventrally or even strongly anterior in some coccosteomorphs (Gardiner & Miles, 1994), suggesting an inserting structure such as an endocranial process of the braincase, rather than a posteriorly directed levator muscle derived from the axial musculature (Stensiö, 1963, p. 194). At a minimum, these pits can be considered to denote the maximum possible anterior extent of the origin of the *m. levator capitis major*. But the posterior location of these pits in *Dunkleosteus*, marking the posterior extent of the neurocranial‐skull roof contact, would prevent any head‐elevating musculature from inserting underneath the head shield. On balance, these observations suggest that the levator muscles in *Dunkleosteus* inserted onto the combined area formed by the posterior braincase and posterior face of the nuchal thickening, described above. Compared to reconstructions in other arthrodires, the contribution of the nuchal thickening to this space seems to have been larger in *Dunkleosteus* than in *Coccosteus* (Figure 15b,c; Miles & Westoll, 1968, fig. 24b) though perhaps more similar to aspinothoracidans (Stensiö, 1963, Figs. 95, 101e, 107c, and 108d).

The *m. levator capitis major* of *Dunkleosteus* and other arthrodires was originally considered to originate on the carinal process of the median dorsal plate (Adams, 1919; Heintz, 1932), which in *Dunkleosteus* is extremely large and posteroventrally prominent. However, Miles (1969) expressed skepticism about this, noting the muscles would have to contract by only 2% of their resting length to elevate the head. This skepticism was later confirmed by direct observation of preserved muscle fibers in other arthrodires, which found the origin of the *m. levator capitis major* was located well anterior to the carinal process (Trinajstic et al., 2013). The origin of the *m. levator capitis major/minor* is in a fairly awkward position in arthrodires, as their origin on the flattened internal surface of the mediodorsal trunk shield plate requires the muscle fibers to pull at an oblique angle and low mechanical advantage (the insertion is otherwise parallel to the muscles' contractile path), but the muscle fibers still clearly insert here and transition to the epaxial musculature. It is possible this is the reason for the palmate origin of the muscle fibers in this region (Trinajstic et al., 2013). The function of the carinal process in arthrodires remains poorly understood.

#### 3.5.2 m. levator capitis minor

The *m. levator capitis minor* of *Dunkleosteus terrelli* likely occupied the triangular‐shaped lateral division of the nuchal thickening. This is largely based on Long et al. (2014, p. 36, fig. 2e), who interpreted the *m. levator capitis minor* as occupying the homologous region in buchanosteids. Trinajstic et al. (2013) report the *m. levator capitis minor* originates within the cucullaris fossa in *Compagopiscis croucheri*, *Eastmanosteus calliaspis*, and *Incisoscutum ritchei* (WAM 85.9.668; Trinajstic et al., 2013, fig. S1H,K,L), but this would not be possible for *Dunkleosteus*. In *Dunkleosteus*, the cucullaris fossa has a strongly developed posterior ridge (Figures 9 and 15), which would prevent any structures originating on the visceral face of the median dorsal plate from attaching inside the cucullaris fossa. Like the Gogo Formation arthrodires, the *m. levator capitis minor* of *Dunkleosteus* appears to be much smaller than the *m. levator capitis major*, though in buchanosteids this relationship is reversed (Long et al., 2014).

Miles and Westoll (1968, p. 393) consider the lateral division of the nuchal thickening to represent the site of articulation between the head shield and the extrascapular plates. However, extrascapular plates are unknown in the large hypodigm of *D. terrelli* (R. Carr and R. Engelman, personal observation 2024), even in articulated specimens (CMC VP 8294, CMNH 8982). One possibility is that the extrascapular plates ancestrally floated on top of soft tissue that attached to this surface (i.e., the *m. levator capitis minor*), whereas in *Dunkleosteus* the extrascapular plates may have been lost but the underlying soft tissue structures remained. It is worth noting that extrascapular plates have only been reported from coccosteomorphs and *Eastmanosteus calliaspis* among eubrachythoracids (Desmond, 1974; Gardiner & Miles, 1990, p. 189; Miles & Westoll, 1968; Stensiö, 1963). Stensiö (1963), despite figuring hypothetical extrascapulars in his reconstruction, did not report them from any pachyosteomorph he examined and admitted they may have been vestigial or absent in these forms (Stensiö, 1963, p. 216). Other studies on pachyosteomorphs have considered these plates probably absent in the taxon examined (e.g., Carr, 1991, p. 353). However, comparatively small extrascapular plates were figured for the pachyosteomorph *E. calliaspis* (Trinajstic et al., 2013, fig. 3a,b), confirming their presence in at least some dunkleosteoids. Even if extrascapular plates were present in *Dunkleosteus*, these contact faces are so small it seems unlikely they would affect gape.

#### 3.5.3 Posterior embayment of the head shield

The skull roof of *Dunkleosteus* has a deep posterior embayment (embayment >10% head shield length), largely formed by the nuchal plate. The nuchal embayment is, on average, ~14% of total head shield length, and there is no clear pattern of positive or negative allometry of nuchal embayment depth with size (File S12). The size of the embayment in *Dunkleosteus* is relatively modest compared to arthrodires like *Heintzichthys* or *Stenosteus*, where it can exceed 25% of total head shield length, but it is much larger than in coccosteomorphs, where the nuchal embayment is poorly developed or absent (<10% head shield length).

A deeply embayed nuchal plate appears to have evolved independently in Dunkleosteoidea and Aspinothoracidi. A posterior embayment of the nuchal plate is absent or very small in *Eastmanosteus calliaspis* (Dennis‐Bryan, 1987), *E. pustulosus* (Eastman, 1898), *“Dunkleosteus” magnificus* (Hussakof & Bryant, 1918, figs. 8 and 9), *Golshanichthys asiatica*, and the possible dunkleosteoid *Westralichthys* (File S12), contra the condition in *D. terrelli*. Similarly, the nuchal is only slightly embayed in the most basal aspinothoracidans like *Bullerichthys*, *Kendrickichthys*, and *Trematosteus*. By contrast, it is prominent in all macropredatory aspinothoracidans where the nuchal plate is known, as well as other early‐diverging aspinothoracidans like *Titanichthys*, *Tafilalichthys*, and *Mylostoma* (File S12). The development of the posterior embayment is highly variable in selenosteids and related forms (File S12), being large and prominent in some species (*Amazichthys*, *Driscollaspis*, *Gymnotrachelus*, *Stenosteus*) and small to absent in others (Brachydeiridae, *Parabelosteus*, *Pholidosteus*). It is not possible at this time to evaluate whether the condition in the latter represents a secondary reversal or if deeply embayed nuchal plates evolved multiple times within aspinothoracidans, especially as most of the forms with highly embayed head shields come from the Famennian and most with poorly developed nuchal embayments are Frasnian.

A posterior embayment of the head shield and larger nuchal gap has often been suggested to be correlated with a greater freedom of movement at the cranio‐thoracic joint and thus a larger gape (Miles, 1969). However, as noted by Miles (1967, p. 62) and Anderson and Westneat (2009, p. 264), it may also increase the mechanical advantage of the *m. levator capitis major*. By displacing the attachment site of the medial fibers of the *m. levator capitis major* anteriorly relative to the point of rotation (the level of the foramen magnum and cranio‐thoracic joint), this creates a longer in‐lever that enhances the leverage of this muscle in cranial rotation and elevation, similar to what has been proposed for some living fishes (Sonnefeld et al., 2014).

#### 3.5.4 m. cucullaris

The cucullaris fossa is located on the ventrolateral side of the endocranium, which most authors have considered the insertion site of the *m. cucullaris* (Heintz, 1932; Miles, 1967, 1969; Stensiö, 1963; Trinajstic et al., 2013). This fossa is deep in *Dunkleosteus* (Figure 9), bordered by a well‐developed inframarginal crista anteroventrally (Xue et al., 2025) and a well‐developed posterior ridge derived from the nuchal thickening posterodorsally (Figure 9, hind thickening in Heintz, 1932, figs. 13 and 14). This posterior ridge is absent in most other arthrodires, including the dunkleosteoids *Eastmanosteus calliaspis* (Dennis‐Bryan, 1987, figs. 7 and 8) and *Golshanichthys* (Lelièvre et al., 1981), with the cucullaris fossa being more open posteriorly. However, it is possibly present in *Westralichthys* (Long, 1987, figs. 4 and 5). The condition in *Mcnamaraspis* suggests it is possible a posterior ridge was present in other taxa but formed by the cartilaginous supravagal processes of the endocranium (Long, 1995, figs. 2 and 7). The deep cucullaris fossa suggests the cranial depressor muscles of *Dunkleosteus* were particularly well‐developed, as has been proposed in previous studies (Anderson & Westneat, 2007, 2009; Heintz, 1932; Xue et al., 2025). The *m. cucullaris* likely inserted onto the mesial ridge continuous with the postbranchial lamina on the internal face of the anterior lateral plate (Heintz, 1932, p. 52), as has been proposed for other eubrachythoracid arthrodires (Miles, 1967, 1969; Trinajstic et al., 2013).

In *Incisoscutum* (WAM 86.9.688), where the *m*. *cucullaris* is preserved, it occupies the entirety of the cucullaris fossa (Trinajstic et al., 2013, fig. S1I,‐J). However, in the phyllolepid *Cowralepis* this fossa is partially denticulated, indicating this fossa in arthrodires may not have solely contained the *m. cucullaris* but also the dorsal part of the parabranchial cavity (Carr et al., 2009). The thickness of the *m. cucullaris* in arthrodires is unclear, but given that at least some of the cucullaris fossa appears to be part of the parabranchial cavity in some taxa, it appears to have been much thinner than in previous reconstructions (Anderson & Westneat, 2007; Heintz, 1932). This was partially discussed by Anderson (2010) with regards to bite force calculations, though not overall jaw kinematics. The cucullaris fossa of *Dunkleosteus* (and other eubrachythoracid arthrodires) needs to be re‐evaluated to determine how much of this region served as an attachment for the *m. cucullaris* (and this muscle's size). *Dunkleosteus* lacks denticles in the cucullaris fossa, but some specimens (CMNH 5544, CMNH 5831) show what appears to be a very slight ridge and difference in bone texture on the surface of the cucullaris fossa, approximately dividing this region in half. This could represent the boundary between the insertion for the *m. cucullaris* and parabranchial chamber, but this feature is too ambiguous to determine for certain and could be an artifact of preservation.

### 3.6 Functional group 6: Mandibular symphysis

Like all lower vertebrates, the mandibles of *Dunkleosteus* articulated with one another across a partially ossified cartilage, the mentomandibular ossification. The two infragnathals did not directly articulate with one another, nor is there evidence that the symphyseal facets of the symphyseal odontoid were connected by ligaments or other soft tissues in life (Hussakof, 1906). The infragnathal has two main contacts with the mentomandibular ossification. The first is a large, slightly concave facet covered in rugose striations located on the internal face of this plate (Lebedev et al., 2023, fig. 3). The second is the Meckelian groove, located on the ventral side of the oral region of the infragnathal and into which a projecting tongue of the mentomandibular ossification fits (Dunkle & Bungart, 1942a; Lebedev et al., 2023).

**FIGURE 16:**
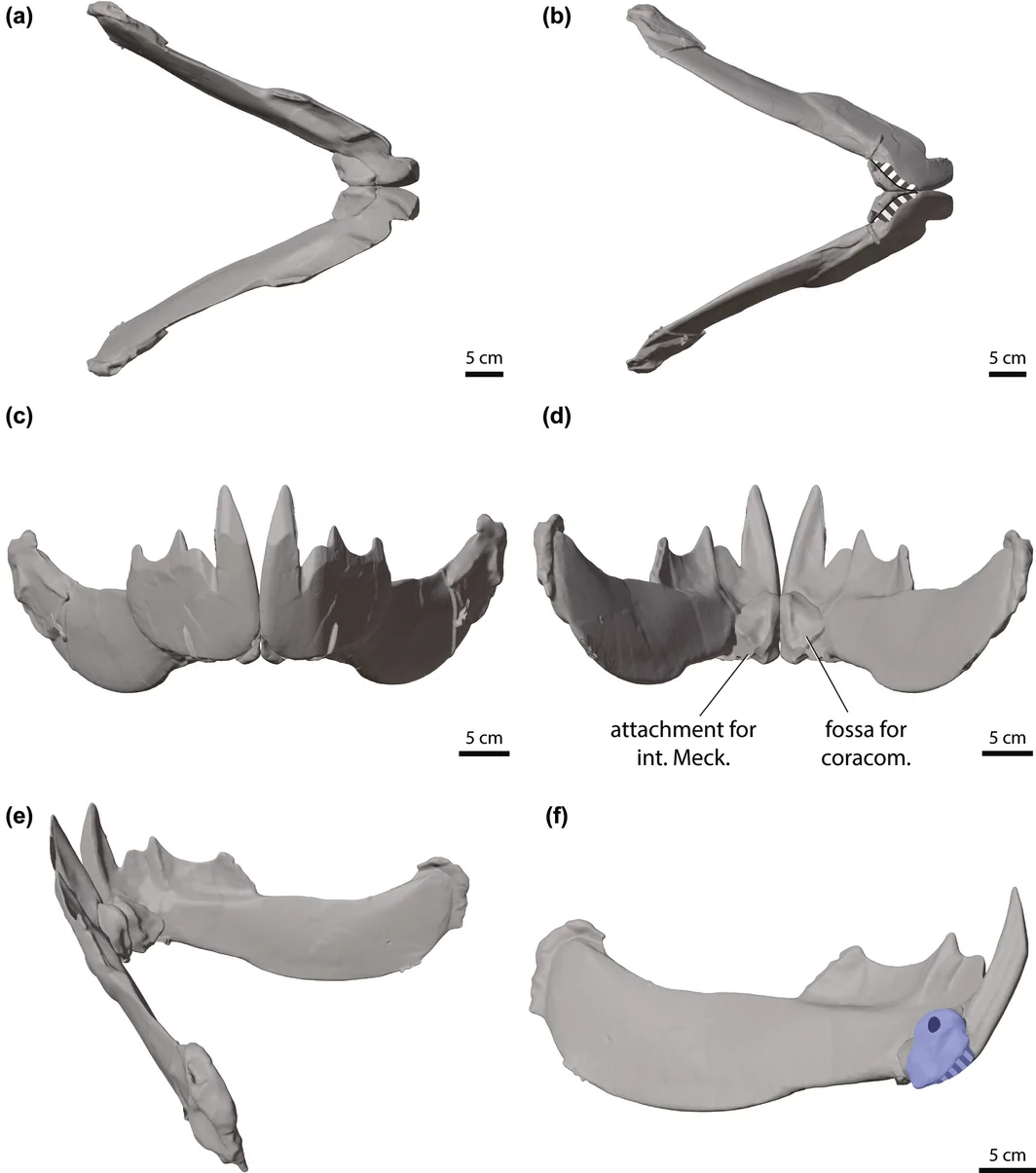
Digital models of the left infragnathal of CMNH 7054 (mirrored) with the mentomandibular ossification placed back in anatomical position, shown in dorsal (a), ventral (b), anterior (c), posterior (d), posterolateral (e), symphyseal (f) views. Symphyseal facet of the mentomandibular ossification is highlighted in blue in (f). Hatched fill represent areas of the infragnathal or mentomandibular ossification covered up in the original model, leaving gaps in the surface mesh when the mentomandibular ossification was rotated back into anatomical position. Medial foramen in (f) artificially emphasized due to its depth failing to be captured on the original surface scan. Abbreviations as in Figures 1 and 2.

The mentomandibular ossification is preserved in several mounted specimens of *Dunkleosteus* (CMNH 5768, CMNH 6090, CMNH 7054), albeit slightly ajar from life position. When the mentomandibular ossification is adjusted in digital models to properly fit the contact face for this element on the infragnathal, the large, flattened facet on its internal face is oriented directly medial (Figure 16f). The overall morphology of the symphyseal face in *Dunkleosteus* resembles that of other arthrodires, particularly in the presence of a large, medially oriented foramen (Johanson, 2003, figs. 4g and 5a; Miles & Dennis, 1979, fig. 11c). In *Eastmanosteus calliaspis* (NHMUK PV P50893) the bone around this foramen forms a medially protruding process that creates a small medial gap between the two infragnathals, but in *Dunkleosteus* this foramen is recessed and the flattened faces of the two elements are able to be in direct contact.

In CMNH 7054, which has one of the best‐preserved mentomandibular ossifications, once these elements are restored in life position the hemimandibles form a well‐developed articulation across the mandibular symphysis (Figure 16). This suggests there was no space for a basimandibular, or at best it was a small element only a few centimeters thick. The condition in *Dunkleosteus* resembles comments by Dennis and Miles (1979a, p. 311) and (1981, p. 229), who note that when the mandibles of *Rolfosteus canningensis* and *I. ritchei* are placed back into articulation the mentomandibular ossifications directly articulate and there is no room for a basimandibular as suggested by Stensiö (1963). Articulating the hemimandibles of *Dunkleosteus* across this symphyseal facet results in the dorsal border of the mentomandibular ossification smoothly grading into the anterior lingual fossa of the infragnathal (Lebedev et al., 2023) to form a continuous fossa (Figure 16d,e), the lower gnathal blades being in the appropriate position to occlude against the opposing upper gnathals, a lateral orientation of the ventral glenoid facet on the articular cartilage (Figure 16c), and the posterior ends of the hemimandibles being appropriately spaced mediolaterally to articulate with the quadrate process of the postsuborbital plate (Figure 16). It also results in the infragnathals being splayed slightly (Figure 16c) with the symphyseal odontoids separated at their tips, replicating the orientation proposed by Heintz (1932, pp. 186–187).

A fossa on the posterior face of the mentomandibular ossification appears to represent the insertion of the *m. coracomandibularis* (Figure 16d,e). Contacts for the unossified intermediate portion of the Meckelian cartilage are also visible on the posterior face of the mentomandibular ossification. Based on preserved entheses in Gogo specimens, the *m. coracomandibularis* of arthrodires appears to have originated on the internal face of the interolateral (Sanchez et al., 2013), and along with the *m. coracohyoideus* and *m. levator capitis major/minor* played a major role in opening the mouth (Carr et al., 2009; Johanson, 2003). In addition, the *m. coracomandibularis* and *m. coracohyoideus* would have contributed to lowering the cranium if the mandible was stabilized by the *m. adductor mandibulae*, as has been described for chimaeroids (Romanov et al., 2024).

**FIGURE 17:**
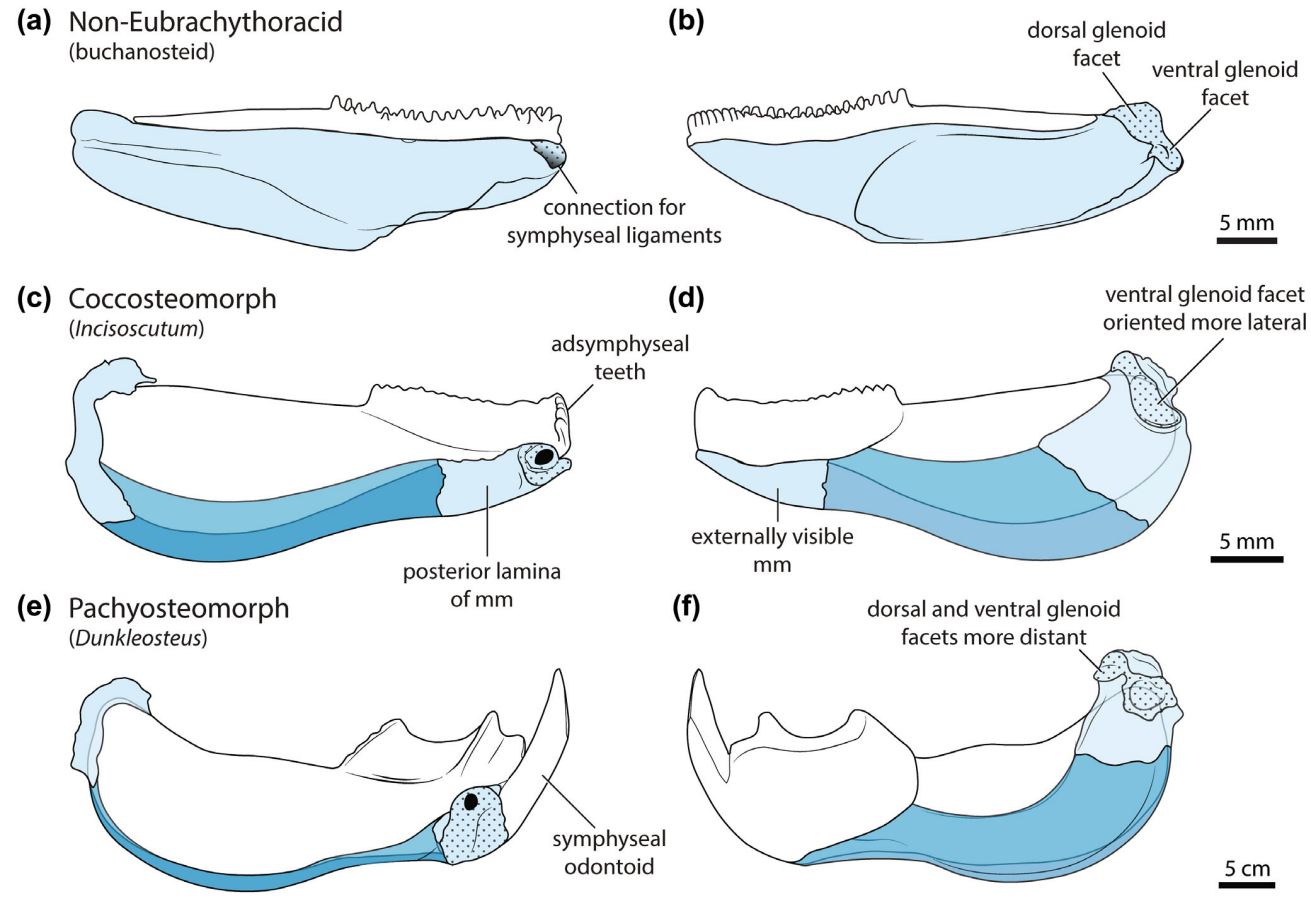
Patterns of change in mandibular morphology across arthrodire evolution, redrawn and modified from Young (2010), fig. 2). Color reflects degree of mineralization; Unmineralized cartilages depicted in dark blue, perichondrally ossified cartilages in light blue, whereas the infragnathal (bone) is depicted in white. Articular sites represented by stippled areas. (a, c, e) in internal view, and (b, d, f) in external view. (a, b) Unnamed buchanosteid (ANU V244), modified from Hu et al. (2017); (c, d) Coccosteomorph (*Incisoscutum*) from Johanson (2003) (WAM 86–9‐683); (e, f) Pachyosteomorph (*Dunkleosteus*) from present study (drawn from CMNH 7054). Abbreviations as in Figure 1.

The mentomandibular ossification of *Dunkleosteus* is less extensive in overall size than other arthrodires. In most eubrachythoracid arthrodires, the mentomandibular ossification is relatively large and is externally prominent beneath the oral region of the infragnathal (Figure 17d) and has a large posterior lamina equal to or greater than the symphyseal surface (Figure 17c). These features occur in, for example, *Harrytoombsia elegans* (Miles & Dennis, 1979, fig. 11), *Eastmanosteus. calliaspis* (NHMUK PV P50893; Dennis‐Bryan, 1987), *Rolfosteus canningensis* (Dennis & Miles, 1979a, fig. 9a), *Compagopiscis croucheri* (Trinajstic & Hazelton, 2007, fig. 5.b2), *Incisoscutum ritchei* (Johanson, 2003, fig. 4g), *Mcnamaraspis kaprios* (Long, 1995, pl.1.3), *Kendrichthys cavernosus* (Dennis & Miles, 1980, fig. 21), *Plourdosteus canadensis* (Ørvig, 1951, pl. 1‐2), and *Coccosteus cuspidatus* (Miles & Westoll, 1968, figs. 21–22). By contrast, in *Dunkleosteus* when the mentomandibular ossification is placed in life position, this element is no longer visible in external view, or at most only a small process is visible (Figures 2 and 17f). Some of this may be related to the great depth of the oral region in *Dunkleosteus*. Similarly, the posterior lamina on the mentomandibular ossification is significantly shorter than the symphyseal face in *Dunkleosteus*, to the point where this structure is almost absent (Figures 16f and 17e). However, in spite of the small size of the mentomandibular ossification as a whole, the symphyseal face of the mentomandibular ossification in *Dunkleosteus* is proportionally larger relative to the infragnathal than in any other arthrodire where this element is known (Figure 17e), including *E. calliaspis* (NHMUK PV P50893; Dennis‐Bryan, 1987, fig. 9). The size of the symphyseal facet in *E. calliaspis* (NHMUK PV P50893) appears to be proportionally larger relative to the infragnathal than in coeval Gogo coccosteomorphs, though more similar in size to these taxa than *D. terrelli*.

The infragnathal of *Dunkleosteus* is also unusual among arthrodires in lacking a series of adsymphyseal teeth along its symphyseal face (Figure 17c). These structures are present in most eubrachythoracid arthrodires (e.g., Dennis & Miles, 1981; Hussakof, 1906; Miles & Dennis, 1979, and see phylogenetic matrices in and see phylogenetic matrices in Boyle & Ryan, 2017, Carr, 1991, Zhu & Zhu, 2013, and Zhu, Zhu, & Wang, 2016), and based on their phylogenetic distribution appear to be present in the MRCA of Eubrachythoraci (File S13). Losses of adsymphyseal teeth are uncommon in eubrachythoracids and generally occur at specific nodes associated with specialized life habits (e.g., filter‐feeding—*Titanichthys*, extreme specializations for durophagy—*Mylostoma*) (File S13). Perhaps most notably, adsymphyseal teeth have been independently lost in the two major groups of large, macropredatory arthrodires—dunkleosteoids and the macropredatory aspinothoracidans (the latter of which may or may not be monophyletic; Carr, 1991)—and in both cases, based on the distribution of this character, appear to be lost within the group. Adsymphyseal teeth are present in the basal dunkleosteoids *Eastmanosteus calliaspis* (Dennis‐Bryan, 1987) and *E. pustulosus* (Hussakof & Bryant, 1918, p. 51) but absent in later members of the group like *D. terrelli*, “*D. newberryi*,” *Golshanichthys*, and present but vestigial (extremely small and likely non‐functional) in “*Dunkleosteus*” *magnificus* (Hussakof & Bryant, 1918, pp. 43–44, fig. 12). In *Elmosteus lundarensis*, adsymphyseal teeth are absent but there is a prominent, raised ridge on the internal face of the symphyseal odontoid that appears to correspond to the remnants of this tooth field (Jobbins et al., 2025, fig. 6e). Similarly, they are absent in the macropredatory aspinothoracidans *Heintzichthys*, *Gorgonichthys*, and *Holdenius*, but present, albeit vestigial, in *Dinichthys herzeri* (Hussakof, 1906). Their condition is unknown in *Hadrosteus*; the symphyseal face of the infragnathal does not appear to be exposed in the holotype and only known specimen (MB.f.163; Stensiö, 1963, pl. 22–24). Although these are not the only arthrodires to have lost the adsymphyseal teeth, dunkleosteoids and macropredatory aspinothoracidans show significant morphological convergences in their cranio‐mandibular anatomy related to macropredatory habits (Carr & Hlavin, 1995, 2010), such as the independent development of symphyseal odontoids. This suggests the convergent loss of the adsymphyseal teeth in these groups may be related to macropredatory specializations. The loss of the adsymphyseal teeth may even be correlated with the development of symphyseal odontoids, though a few species like *D. herzeri* and *E. pustulosus* have both (Hussakof, 1906, fig. 25c; Hussakof & Bryant, 1918, p. 13.2).

The exact function of the adsymphyseal teeth in arthrodires remains unknown. The presence of these teeth suggests the symphyseal faces of the infragnathals were not linked by soft tissue (Hussakof, 1906), with the two hemimandibles articulating only across the mentomandibular ossification. The position of these teeth is odd because they point toward the midline without any clear antagonist (except potentially the symphyseal teeth on the opposite infragnathal). In some taxa (*Mcnamaraspis kaprios*, Long, 1995, pl. 1.6–7; *Plourdosteus canadensis*, Vézina, 1986, fig. 4; *Protitanichthys rockportensis*, R. Carr personal observation 2024; *Torosteus* sp., AMNH FF 19499), there may also be adsymphyseal teeth on the anteroexternal edge of the anterior supragnathals as well. These teeth tend to be more frequent in juvenile specimens but rarer in older individuals. As with the adsymphyseal teeth on the lower jaw, the orientation of these teeth toward the midline suggests they had little occlusion against teeth from the mandible. Newberry (1878, 1889, p. 159) and Hussakof (1906, p. 122) suggested the adsymphyseal teeth of the infragnathal meant the two halves of the mandible were prehensile at the symphysis and could be used to grip food, particularly based on their examination of *Diplognathus*. However, this function seems unlikely as gripping and swallowing food between the adsymphyseal teeth would result in prey bypassing the gripping teeth, shearing blades, or crushing surfaces on the occlusal margin. Similarly, the adsymphyseal teeth may not be capable of prehension if the infragnathals were separated by a significant medial gap. Hussakof (1906, p. 118, 122) concluded the adsymphyseal teeth were functional and clearly played an important role in the functional morphology of the arthrodire jaw but concluded “[w]hat the exact function of the symphyseal denticles was cannot be definitely ascertained.” We agree the adsymphyseal teeth were functional, given they are a complex structure that affected jaw function enough for natural selection to independently favor their loss in macropredatory arthrodires and other forms associated with specialized feeding habits, but like Hussakof (1906) we are unable to offer a reason for their existence.

## 4 DISCUSSION

### 4.1 Long axis rotation of the jaws in eubrachythoracid arthrodires

Long axis rotation of the mandible in jaw kinematics is a common feature among vertebrates (Bhullar et al., 2019; Lebedev et al., 2022; Scott et al., 2022; Summers et al., 2004), particularly those with wide mouths. This includes arthrodires, for which some degree of long axis rotation at the symphysis has been proposed for a number of species (Dean, 1901; Heintz, 1932; Hussakof, 1906; Vézina, 1986, but see Miles, 1969). Dean (1901, pp. 105–107) considered long axis rotation of the mandible necessary in the durophagous arthrodire *Mylostoma* to produce observed wear patterns on the fossils. This interpretation was criticized by Eastman (1906, p. 136) but the latter interpretation has been questioned in turn because Eastman's interpretation was based on unassociated gnathal plates (B. Dean in Hussakof, 1906, pp. 119–120). Hussakof (1906, p. 115, 118, 123) considered the mandibles of most arthrodires to be mobile or separated at the symphysis due to the presence of symphyseal teeth, but at the same time noted wear patterns in *Dunkleosteus* suggested the mandible did not rotate during jaw closure (Hussakof, 1906, p. 113) and did not consider the effect of the mentomandibular ossification (which was unknown at that time). Miles (1967, p. 58); Miles (1969, p. 137) considered arthrodires to lack long axis rotation due to the shearing wear facets present in many species and an assumed scissor‐like action of the jaw. Vézina (1986, fig. 7, p. 378) provided the most extensive evaluation of long axis rotation in any arthrodire (specifically *P. canadensis*). Vézina (1986) noted the anterior supragnathals of *Plourdosteus* showed greater wear than the posterior, which indicates the jaws could not have closed with a scissor‐like action (which would result in the posterior supragnathals contacting the infragnathal first and thus showing greater wear). Similarly, the supragnathals were noted to show more wear than the infragnathals, which was interpreted as showing a more complex jaw motion. Instead, after reviewing the available anatomical evidence, Vézina (1986) proposed the most likely pattern of jaw closure in *P. canadensis* involved externally directed long axis rotation of the mandible due to kinesis at the cranio‐mandibular articulation and symphysis, with significant rotation occurring at the symphysis near the level of the adsymphyseal teeth (Vézina, 1986, p. 378, fig. 7a).

Long axis rotation in vertebrates often involves obliquely oriented, mediolaterally asymmetrical articulations between the cranium and mandible. The shape of these joints often forces the mandibles to rotate along their long axis as the jaws open to maintain proper contact between the articular facets or prevent parts of the jaw articulations from intersecting (Frey et al., 2020; Schade et al., 2023). Such an arrangement is readily visible in buchanosteids (Hu et al., 2017, fig. 4e), in which the dorsal and ventral mandibular articulations are poorly separated and form a broad dorsomedial–ventrolaterally oriented articular surface, with the more dorsal articulation being more medially and anteriorly positioned, suggesting long axis rotation was present in this form. Eubrachythoracids keep the positional relationship between these dual articulations (Figure 17d), but the two articular surfaces are more distinct. The ventral articulation typically has a more lateral orientation and is often well‐excavated, forming the cotyle of a ball‐and‐socket joint with the articular condyle on the palatoquadrate (Johanson, 2003, fig. 4f; Miles & Dennis, 1979; Stensiö, 1963). There is some variation in this condition within Eubrachythoraci; Miles and Dennis (1979) note that while in *Harrytoombsia* the ventral articular facet is laterally positioned and well separated from an unossified anterodorsal area that based on other arthrodires appears to represent the dorsal articulation (as is the case in most incisoscutoids and pachyosteomorphs; see Dennis & Miles, 1979a; Johanson, 2003; Stensiö, 1963, present study), they also note the ventral articular facet in *Coccosteus* is more posterodorsal, poorly distinguished from the dorsal facet, and lacks a well‐excavated fossa (Miles & Westoll, 1968, p. 415). The latter condition is roughly intermediate between the state in buchanosteids and incisoscutoids/pachyosteomorphs. Chondrichthyans—both extant taxa (Dearden et al., 2021; Moss, 1972; Motta & Wilga, 1995, 1999) and the extinct *Denaea*, *Symmorium*, and *Ferromirum* (Frey et al., 2020)—show a near‐identical arrangement to arthrodires with an anterior and medially positioned dorsal facet and a more posterior and lateral ventral one, and in some taxa the two facets may be well differentiated as in eubrachythoracids (Motta & Wilga, 1995; Motta & Wilga, 1999). Frey et al. (2020, p. 8) found this morphology produced significant external long axis rotation during biting and suggested such a morphology might be suggestive of long axis rotation more generally, especially if movement at the symphysis is not restricted (as is the case in most arthrodires). The near‐identical morphology of this joint in arthrodires suggests long axis rotation of the mandible in these forms.

Kinesis across the mandibular symphysis of *Dunkleosteus* appears to have been limited to absent, suggesting little if any long axis rotation was present. The symphyseal contact on the medial face of the mentomandibular ossifications of *D. terrelli* is large, flat, and about half of the total dorsoventral height of the body of the infragnathal, which would have restricted movement between the two hemimandibles beyond the limited flexibility allowed by perichondrally ossified cartilage. In some specimens, the left and right mentomandibular ossifications are even fused at the midline of the animal (Carr et al., 2009, p. 795), which would prevent long axis rotation altogether. The great height of the symphyseal odontoids would also prevent inward rotation to a degree, and these structures would have needed to maintain a precise, consistent orientation in order to slot into the concave anterointernal face between the two pairs of odontoids on the anterior supragnathals, as wear facets indicate was the case (Dunkle & Bungart, 1946, pp. 3–4; Heintz, 1932; Hussakof, 1906, pp. 111–112). By contrast, significant external long axis rotation would result in the symphyseal odontoid being obliquely oriented relative to jaw motion during biting, and thus poorly positioned to penetrate prey.

A lack of significant long axis rotation is further supported by wear patterns on *Dunkleosteus* gnathals (Hussakof, 1906, p. 113), which suggest a relatively simple orthal shearing motion. Unlike the pattern for *Plourdosteus* described by Vézina (1986), the gnathals in *Dunkleosteus* do not show more wear anteriorly than posteriorly (beyond that induced by the odontoids) (File S14). This agrees with suggestions by Anderson (2008a) that the gnathals of *Dunkleosteus* may have closed with a more uniform, “chopping” motion than a scissor‐like action. However, contra Anderson (2008b) the center of the dominant (ventral) glenoid facet appears to be roughly in line with the occlusal plane on the infragnathal (Figure 17c), which would support a scissor‐like action. Rates of wear are roughly comparable between paired posterior supragnathal and infragnathals in small (CMNH 6194), moderately large (CMNH 7054), and very large (CMNH 5768) individuals, though in some cases wear on the posterior supragnathal may be slightly greater (File S14). This slight difference may be a consequence of the contact between the gnathals being oriented more oblique than vertical in life (Heintz, 1932, pp. 186–187).

It is very surprising then, that *Dunkleosteus* shows the same type of articulation thought to produce long axis rotation in chondrichthyans and other arthrodires, with a dorsal articular facet positioned anterior to a ventral one (Figure 3f,h). One possibility is that *Dunkleosteus* inherited this type of articular arrangement from ancestors that did show significant long axis rotation, but secondarily lost or limited the ability for long axis rotation. The dual jaw articulation might instead be retained for buttressing the jaw. The dorsal and ventral articulations of *Dunkleosteus* appear less mediolaterally separated than other arthrodires, which might support this interpretation as it would reduce possible rotation at the cranio‐mandibular joint. However, it is possible the lesser separation of these facets mediolaterally in *Dunkleosteus* is due to the articular cartilage being flattened mediolaterally during preservation. This broadly agrees with Frey et al. (2020, p. 8), who suggest that such an articular arrangement might suggest long axis rotation only if not already restricted by other factors such as an extensive mandibular symphysis. Evidence supporting this comes from extant chimaeroids, which retain the dorsal and ventral cranio‐mandibular articulation seen in other chondrichthyans (Dearden et al., 2021) despite long axis rotation being impossible due to fusion of the mandibular symphysis. Testing these hypotheses requires better preserved mandibular arch cartilages and direct modeling of jaw kinematics in *Dunkleosteus*. The effect of a dual arthrodire/chondrichthyan‐type jaw articulation on long axis rotation needs to be investigated in more detail.

The apomorphic, extensive symphyseal contact and seemingly reduced long axis rotation in *Dunkleosteus* compared to the general eubrachythoracid condition resemble patterns in other vertebrates. In many groups, taxa that feed on large prey and have high bite forces have more extensive and restrictive mandibular symphyses that stabilize the jaw (Gregory & Conrad, 1936; Holliday & Nesbitt, 2013; Lappin et al., 2017; Scapino, 1981; Therrien, 2005), especially in animals that engage in oral processing—like arthrodires—and do not simply swallow their prey whole. Neoselachians are a major exception to this (but see below), but this is primarily because the protractable jaw kinematics of these taxa necessitate extensive long axis rotation across the mandibular symphysis (Scott et al., 2022; Summers et al., 2004) regardless of dietary habits. A more robust symphysis allows for better unilateral transmission of muscle forces across the symphysis, dissipation of stress, resisting reaction forces, and keeping the mandibles from dislocating when dealing with large prey (Gregory & Conrad, 1936; Huber et al., 2008; Rosen et al., 2025). Similarly, in organisms with a “double guillotine” system of oral occlusion like *Dunkleosteus*, the jaw must be kept highly stable during the bite stroke or else the blades will be pushed out of alignment by the food item and fail to cut (Anderson & LaBarbera, 2008, p. 3625). The patterns of variation seen in the symphyses of arthrodires resemble those of other vertebrates: the more extensive and restrictive mandibular symphysis of *Dunkleosteus* with previous suggestions that this taxon hunted large prey (e.g., Capasso et al., 1996; Hall et al., 2016; Hussakof, 1906), whereas the more ecologically generalized arthrodires from the Gogo Formation have a more mobile symphysis.

The differences in symphyseal morphology between *Dunkleosteus* and generalized, non‐macropredatory eubrachythoracid arthrodires can be loosely analogized to what is seen in mammals, where generalized, plesiomorphic forms have relatively unrestricted symphyseal and cranio‐mandibular articulations and are capable of more complex jaw movements (Bhullar et al., 2019), whereas macropredatory forms restrict complex jaw movements in favor of simple translational rotation and orthal shear, related to maintaining precise occlusion of the shearing dentition (Crompton et al., 2006; Evans & Fortelius, 2008; von Koenigswald, 2016). These similarities are likely due to similar functional demands in these otherwise disparate taxa, as, unlike most vertebrates, both mammals and arthrodires rely on precise occlusion for prey processing, and the sharp edges of the sectorial dentition are maintained by honing between opposing, unreplaced elements (gnathal blades and unreplaced carnassial teeth) rather than polyphyodonty. Indeed, the morphology of the occlusal facets formed between the posterior supragnathal and infragnathal in *Dunkleosteus* (Heintz, 1932, fig. 77) is almost identical to that formed between the carnassial teeth of carnivorous mammals (von Koenigswald, 2016, fig. 2). This, in turn, is probably related to the consistently tough yet ductile material properties of vertebrate flesh (Anderson & LaBarbera, 2008), and therefore the types of adaptations best suited to process it.

Similarly, the more robust, restrictive jaw symphysis and highly ossified jaws of *Dunkleosteus* resemble the condition in tiger sharks (*Galeocerdo cuvier*). Among living sharks, tiger sharks (*G. cuvier*) are notable in having broad jaws that are highly calcified with two to four layers of multiple‐monolayered tessellated calcified cartilage (Dingerkus et al., 1991; Moss, 1972; Witzell, 1987) and—unlike most extant chondrichthyans—fused at the mandibular symphysis (Moss, 1972, p. 427). Whether the symphyseal morphology of tiger sharks also precludes long axis rotation is unclear, though plausible. This increased calcification and robust symphysis enable tiger sharks to bite through hard‐shelled prey like cheloniid sea turtles (Witzell, 1987, p. 25), which are otherwise avoided by large sharks (Heithaus et al., 2008). One could easily envision the extensive and robust mandibular symphysis of *Dunkleosteus* allowing a similar function, given the abundance of armored placoderms in Devonian ecosystems (Carr & Jackson, 2008).

### 4.2 Was *Dunkleosteus* a suction feeder?

Previous paleobiological studies on *Dunkleosteus* have suggested this taxon “may have utilized an early form of suction feeding” (Anderson & Westneat, 2007, p. 77) or “possibly utilized suction forces during prey capture” (Anderson & Westneat, 2009, p. 263). This conclusion was primarily based on biomechanical modeling, specifically calculations of rapid gape expansion as well as large changes in oropharyngeal cavity volume across the gape cycle (Anderson, 2010; Anderson & Westneat, 2007, 2009). However, some of the anatomical assumptions used to create these models have been challenged (see Carr et al., 2009, as well as the present study, particularly regarding the *m. adductor mandibulae* and lateral consolidated region), suggesting a need for a re‐evaluation of the evidence for suction feeding in *Dunkleosteus* from an anatomical perspective.

Several aspects of the functional anatomy of *Dunkleosteus* are seemingly at odds with suction feeding. Perhaps most notably, the dentition of *Dunkleosteus* is very unlike a suction feeder. Suction‐feeding fishes typically have dentitions consisting of large, flat patches of villiform teeth and lack large piercing structures like caniniform teeth or odontoids (Gosline, 1973; Mihalitsis & Bellwood, 2019, 2021; Wilga et al., 2007). Indeed, large caniniforms on the marginal dentition might impede suction feeding by disrupting suction flows bringing food into the mouth, and vice versa with suction feeding potentially drawing prey in beyond the oral rim and making the marginal dentition functionless (Berkovitz & Shellis, 2017, p. 47; Gosline, 1973). The tooth plates of suction feeders serve to hold prey after it has been drawn into the mouth until it can be repositioned and swallowed whole (Gosline, 1973; Mihalitsis & Bellwood, 2019, 2021; Wilga et al., 2007). Because of this, most suction‐feeding fishes are also gape‐limited and engage in little if any oral processing of prey—that is, they do not bite or tear their prey into swallowable pieces while feeding and are limited to consuming prey narrower than the diameter of their mouth (Grubich et al., 2008; Mihalitsis & Bellwood, 2017; Wilga et al., 2007). By contrast, fishes that actively pursue their prey and bite chunks out of them, such as barracudas, predatory characiforms, and large sharks, tend to be ram feeders and make little, if any, use of suction (Gosline, 1973; Grubich et al., 2008; Klimpfinger & Kriwet, 2020). The tusk‐like odontoids and bladed oral regions of *Dunkleosteus* are much more similar to extant ram feeders that orally process prey (“dismemberment” or “chopping” predators, sensu Engelman, 2023b; Helfman et al., 2009). This is further supported by functionally analogous features in the dentitions of living animals like hyaenid and felid mammals. The carnassial teeth of these taxa, which like the oral blades of large arthrodires like *Dunkleosteus* are hypertrophied, blade‐like structures posterior to the caniniforms, are primarily used to cut through tougher tissues like skin and break prey items down into manageable pieces (Van Valkenburgh, 1996). By contrast, other arthrodires for which suction‐feeding has been proposed (e.g., *Cowralepis*) differ from *Dunkleosteus* in having broad, flattened, and denticle‐covered mouthparts resembling the villiform tooth patches of living suction‐feeding fishes (Carr et al., 2009, p. 800; Ritchie, 2005). This suggests the dentitions of placoderms and crown‐group gnathostomes exhibit similar functional patterns and the dissimilarity between the dentitions of *Dunkleosteus* and living suction‐feeding fishes cannot be explained by phylogenetic distance. While other evidence against suction feeding in *Dunkleosteus* might be challenged by differing reconstructions of unpreserved soft tissues (i.e., labial cartilages) or paleoenvironment, gnathal morphology represents a clear, directly observable character state that argues against suction feeding in this taxon.

The large mouth and posterior position of the jaw articulation might also argue against suction feeding in *Dunkleosteus*. In general, suction feeding performance tends to show an inverse correlation with the relative size of the oral opening. Larger mouths enable fishes to consume larger prey but reduce the pressure disparity created across the oropharyngeal cavity (Wainwright et al., 2007; Wainwright & Day, 2007). Lateral occlusion of the gape is particularly important, as otherwise water will enter the oropharyngeal cavity lateral to the mouth during feeding (Janovetz, 2002), reducing effective suction force on prey items directly in front of the predator. However, such an arrangement is beneficial for ram feeders, as it allows water to more easily escape from the oral cavity and avoid generating a bow wave during a feeding strike (Ferry et al., 2015, pp. 4–5; Gosline, 1973; Wainwright et al., 2015, p. 140). As a result, most suction feeding fishes have some kind of structure restricting the oral opening, which increases pressure differentials across the oral/extraoral boundary (Ferry et al., 2015; Moss, 1977). Teleosts accomplish this with a highly protractile premaxilla or maxilla (Lauder, 1979), whereas in sharks this is achieved with prominent labial cartilages (Klimpfinger & Kriwet, 2023; Moss, 1977; Wilga et al., 2007). Ram‐feeding pursuit predators such as barracuda, mackerels, predatory characiforms (*Hydrocynus*, Serrasalminae) and several groups of sharks (lamnids, carcharhinids, and hexanchiids) tend to lose or never develop these features, either via a loss of cranial kinesis (in osteichthyans; Gosline, 1973; Grubich et al., 2008; Westneat, 2004) or highly reduced/vestigial labial cartilages (in sharks; Klimpfinger & Kriwet, 2020).

The lack of lateral gape occlusion is a particular issue for *Dunkleosteus* given its extremely large mouth and posteriorly placed jaw angles relative to head/oropharyngeal size (Engelman, 2023b). This arrangement would be expected to result in poorer suction performance compared to other arthrodires, though it might assist ram feeding. Anderson and Westneat (2009) acknowledged this as a potential weakness in their model, though they suggested it might be resolvable if *Dunkleosteus* had soft tissue structures occluding the sides of the mouth, although anatomical evidence for such structures was not presented.

In general, evidence for structures restricting the oral opening in *Dunkleosteus* is poor. The mouthparts of *D. terrelli* were not protractible and there is little evidence for the presence of well‐developed labial cartilages. As mentioned above under “Suborbital groove and *m. preorbitalis*” labial cartilages have not been found in eubrachythoracid arthrodires, even in those preserving endoskeletal elements or soft tissues (Trinajstic et al., 2013; Trinajstic, Long, et al., 2022). One possibility is that the labial cartilages were small and/or unmineralized, as some other cartilaginous elements like gill arches are rarely preserved in arthrodires and their absence is thought to reflect poor mineralization (Brazeau et al., 2023; Carr et al., 2009; Johanson, 2003; Stensiö, 1963, p. 374). However, in suction‐feeding sharks, the labial cartilages are typically very large and robust in order to endure the high suction forces generated during feeding (Klimpfinger & Kriwet, 2023). The degree to which labial cartilages are mineralized across chondrichthyans has not been extensively investigated, but in at least some suction‐feeding species with prominent labial cartilages, these structures are also highly mineralized (e.g., *Heterodontus*; Summers et al., 2004, p. 6). If analogous labial cartilages were present in *D. terrelli*, they would be expected to be large, robust, and highly mineralized (i.e., perichondrally ossified, like the mentomandibular ossification), and thus readily preserved. However, as noted above, labial elements are unknown in specimens of *Dunkleosteus*, even those with articulated mouthparts. Similarly, in many sharks with prominent labial cartilages, the proximal ends of the upper and lower labial cartilages often (but not always) have well‐developed ligamentous attachments on the lateral surface of the mandibular arch (Klimpfinger & Kriwet, 2023; Motta & Wilga, 1999). Analogous ligamentous attachment sites are not apparent on the mandibular arch of *D. terrelli*.

Additionally, if large labial cartilages were present in *Dunkleosteus*, they would potentially risk occluding the gape. In chondrichthyans, the labial cartilages must protract the soft tissue angles of the jaw anteriorly as the mouth opens to keep from dislocating (Moss, 1977; Motta & Wilga, 1999, p. 58; Wu, 1994). However, given the posterior location of the angle of the jaws in *Dunkleosteus*, this would result in the labial cartilages and associated soft tissues moving so far forward when the mouth opened that they potentially obstruct the blades on the posterior supragnathal and infragnathal, preventing the gnathal blades from functioning upon prey contact. Lateral occlusion of the gape by labial cartilages would also result in *Dunkleosteus* being gape‐limited, which is contradicted by fossil evidence. Bite traces from large arthrodires have been recorded on other large organisms, including other arthrodires (Anderson & Westneat, 2009; Capasso et al., 1996; Hall et al., 2016; Hussakof, 1906) and ctenacanth sharks (Hlavin, 1990). Preserved stomach contents suggest smaller arthrodires were not gape‐limited, either. For example, in the Gogo fauna, phyllocarid crustaceans (15 cm in length, approximately half the length of the individual) have been found as gut contents of *Compagopiscis croucheri* (Trinajstic, Long, et al., 2022), and in Russia, plates from a juvenile ptyctodont *Ctenurella*, apparently swallowed whole, have been found in the gut of a plourdosteid (Zakharenko, 2008). Thus, arthrodires are unlikely to have been gape‐limited predators, suggesting they are unlikely to have had prominent labial cartilages.

Liem (1993), focusing on actinopterygians, discussed morphological features characterizing the three main feeding modes in fishes: suction feeding, ram feeding, and biting. Some of the features seen in *Dunkleosteus* resemble those Liem (1993) identifies in suction feeders, including large *m. adductor mandibulae*, *m. levator palatoquadrati/levator arcus palatini*, and *m. levator capitis major*/*m. epaxialis*, short preorbital length, wide head, and deep suspensory apparatus. However, most of these are also shared with taxa specialized for high bite force (Janovetz, 2002; Liem, 1993). *Dunkleosteus* also shows several features Liem (1993) considers not typical of suction feeders and more similar to ram feeders, including a lack of protrusible jaws, a robust rather than slender mandible, a terminal and non‐superiorly oriented mouth, an anteroposteriorly elongate mandibular arch (especially relative to other arthrodires), and a large gape. The latter two features are expected to significantly compromise suction feeding.

Interpretation of other features seemingly indicative of suction feeding may be contradicted by arthrodire or actinopterygian‐specific features and constraints. For example, while suction‐feeding actinopterygians tend to have large cranial elevator muscles (Liem, 1993; Sonnefeld et al., 2014), in arthrodires this correlation is confounded by the connection between the head and trunk armor requiring significant cranial elevation for any degree of mouth opening (Heintz, 1932; Miles, 1967, 1969). In fact, in chondrichthyans, ram‐feeding species typically show high rates of cranial elevation related to increasing vertical gape, whereas suction‐feeding species show little to no cranial elevation (Motta & Wilga, 2001; Wilga et al., 2007), contrary to the pattern seen in actinopterygians. This suggests a straightforward relationship between epaxial muscle size and feeding style cannot be uncritically applied across gnathostomes. Indeed, given the broad‐scale (plesiomorphic) similarities in the feeding apparatus of arthrodires and chondrichthyans compared to actinopterygians, the large epaxial muscles of *Dunkleosteus* could be interpreted as related to increased gape and thus supporting ram feeding. The short preorbital length may also be a conserved plesiomorphy, given arthrodires generally show statistically shorter snouts than extant eugnathostomes (Engelman, 2023a), though some taxa did develop elongate rostra (Carr, 2004a). In summary, *Dunkleosteus* shows a combination of features typical of fishes that use ram feeding and suction/biting. Given some of these features actively reduce the effectiveness of suction feeding but compromise ram feeding to a much lesser degree, the cranio‐mandibular morphology of *Dunkleosteus* is probably best described as the result of a series of tradeoffs between ram feeding and biting.

Suction feeding in *Dunkleosteus* is unlikely from a paleobiological perspective. The effectiveness of a suction current is proportional to both suction force and prey mass, meaning larger prey are more resistant to suction and can more easily generate thrust to escape a current. Suction currents are only able to draw in prey items directly in front of the mouth (Nauwelaerts et al., 2008; Wilga et al., 2007), and as a result, are less effective against highly elusive, nektonic prey such as neritic‐pelagic fishes and cephalopods. Suction force also declines exponentially with distance (Ferry et al., 2015; Wainwright et al., 2001; Wilga et al., 2007), meaning fishes relying on suction to feed must be very close to their prey (0.5–1 gape diameters; Jacobs & Holzman, 2018; Motta et al., 2008; Wilga et al., 2007; Yaniv et al., 2014) before opening their mouth. This makes suction feeding unreliable in open water habitats, with fishes in these environments usually chasing prey down and attacking it tail‐first (Mihalitsis & Bellwood, 2017, 2021). Indeed, most open water piscivores, including carcharhinid and lamnid sharks, istiophoriforms, most scombrids, sphyraenids, and some larger carangids, are ram‐feeders (Grubich et al., 2008; Juanes et al., 2002; Klimpfinger & Kriwet, 2020). Convergent tendencies toward ram‐feeding behaviors in distantly related open water chondrichthyans and bony fish suggest placoderms would be expected to show similar patterns. Indeed, if suction was present in any eubrachythoracid, it would be more expected in the more generalized, non‐pelagic taxa like coccosteomorphs than in pelagic taxa like *Dunkleosteus*, given the functional patterns seen in modern fishes (see Anderson, 2010, pp. 1001–1002 for discussion about potential suction feeding in these forms).

The Cleveland Shale—the stratigraphic unit which has produced the vast majority of *Dunkleosteus terrelli* specimens—is thought to have formed in an open water paleoenvironment (see discussion in Carr, 2010; Engelman, 2024). Fossilized gut contents from other fishes from this environment like *Cladoselache* resembled modern ram‐feeders in swallowing their prey tail‐first (Williams, 1990). However, while feeding traces are known, in situ gut contents that could determine prey orientation in Cleveland Shale arthrodires are unknown (Williams, 1990). Suction is also thought to be rare in nektonic sharks due to these taxa lacking a swim bladder, which prevents them from remaining stationary in the water column and thus more precisely positioning themselves relative to prey as needed for a suction strike (Wilga et al., 2007, p. 66). Swim bladders are also apparently absent in arthrodires (Trinajstic, Long, et al., 2022), arguing against suction feeding.

Other authors have questioned the presence of suction feeding in *Dunkleosteus*. Scott (2016) suggested *Dunkleosteus* was incapable of true suction feeding, using mathematical modeling to show opening the mouth alone would not result in a sufficient change of the three‐dimensional volume of the oropharyngeal cavity to produce suction. Many fishes accomplish this by retracting their branchial arches in tandem with opening the mouth, but Scott (2016) suggested *Dunkleosteus* may not have been capable of this due to the bony postbranchial lamina of the interolateral plate restricting posterior retraction of the pharynx. Similarly, the present study suggests lateral expansion of the gape, which also helps increase oropharyngeal volume during suction (Lauder Jr, 1985), would not have been possible due to the postorbital and rostral connections between the cheek and neurocranium. Instead, Scott (2016) suggested what little pressure differential was generated when the mouth opened served to compensate for drag during ram feeding, specifically the drag produced when a large, blunt‐headed arthrodire opened its mouth to bite. Similar suggestions have been made regarding the role of oropharyngeal pressure differentials in bite kinematics of otherwise ram‐feeding fishes (Wainwright et al., 2007). Anderson and Westneat (2009) also suggested something similar as an alternative functional model for their findings: an initial ram‐feeding strike with suction used to transport prey deeper into the pharynx, similar to snapping turtles (Chelydridae).

One area that might prove fruitful for future research on this topic and the functional morphology of *Dunkleosteus* in general is the morphology of the branchial chamber and gill arches. The extreme development of the mandibular arch posteriorly, the expansion of the lateral consolidated region medially, and the deeper cucullaris fossa might all be expected to restrict available space for the branchial arches, despite their extent being posteriorly limited by the postbranchial lamina (Carr et al., 2009; Heintz, 1932; Johanson & Smith, 2003; Storrs et al., 2008). This might result in a longitudinally narrower gill chamber compared to other arthrodires (File S8B,C), though at the same time the deeper head and trunk of *Dunkleosteus* might increase the size of the chamber dorsoventrally (Engelman, 2024). Such an arrangement could imply a reliance on ram ventilation over buccal pumping to meet respiratory needs, which might further support proposals here. This is not even touching on how the branchial skeleton would be expected to inform feeding behavior due to its role in suction in fishes (Scott, 2016; Wilga et al., 2007) or serving as attachment points for the *m. coracohyoideus* and *m. clavobranchialis* (Johanson, 2003), as well as the proposed osteichthyan‐like ligamentous connection between the branchial skeleton and mandibular arch (Carr et al., 2009). Unfortunately, as mentioned, branchial elements are only known from a handful of arthrodires, due to seemingly having been poorly mineralized (Brazeau et al., 2023; Carr et al., 2009; Johanson, 2003; Stensiö, 1963, p. 374). Some basibranchial elements have been reported for *Dunkleosteus* (Storrs et al., 2008), but these are fragmentary, isolated, and yet to be fully described.

### 4.3 Gape of Dunkleosteus

Ram‐feeding and biting/oral processing are often paired with a wide gape (Wilga et al., 2007). The jaw muscles as reconstructed here result in a relatively wide gape for *Dunkleosteus*. Early studies on arthrodire feeding behavior suggested, but did not quantify, a wide gape for this taxon (e.g., Miles, 1969). By comparison, Anderson and Westneat (2009) suggested *Dunkleosteus* had a relatively narrow maximal gape of only 45°. A later study (Anderson, 2010) suggested an even narrower gape angle of only 22° for *D. terrelli* based on inferred contractile lengths and muscle strain. Anderson (2010) suggested the gape of 45° proposed by Anderson and Westneat (2009) was still possible, but required increasing the contractile length to 10% and “resulted in the adductor muscles being strained well beyond values seen in modern groups” (Anderson, 2010, p. 996). This conclusion was in part based on these studies' reconstructed path for the *m. adductor mandibulae*, which originated on the lateral consolidated area and had an insertion area extending to the ventral margin of the infragnathal. This meant the jaws could not be opened very wide before the mandibular articulation interfered with the contractile path of the *m. adductor mandibulae* (Anderson & Westneat, 2009, pp. 259–260).

**FIGURE 18:**
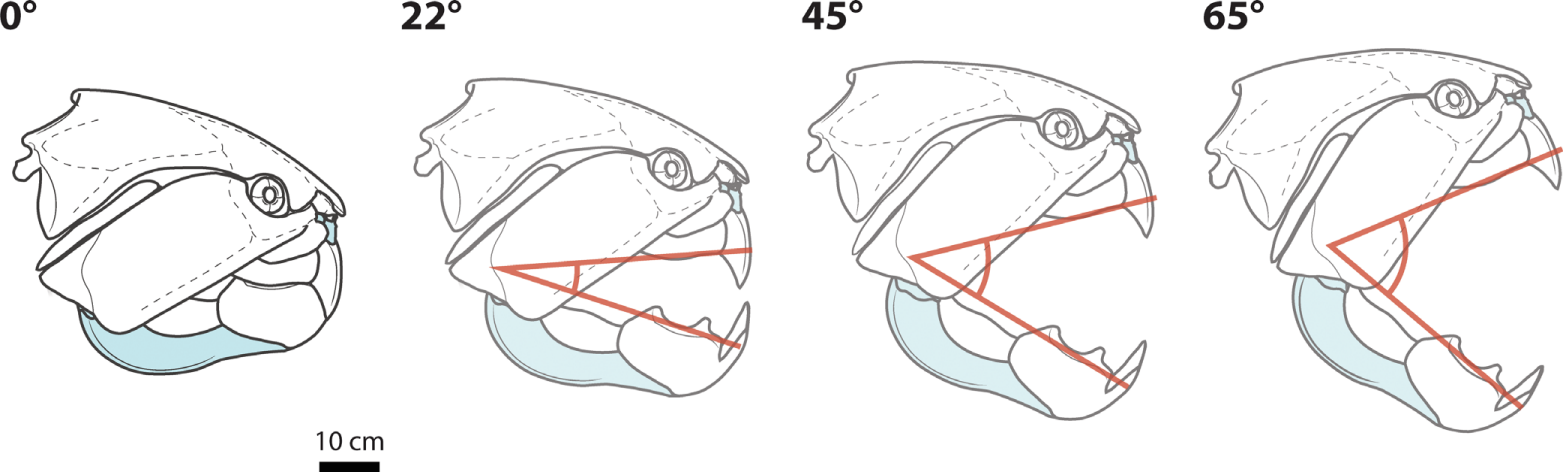
Gape angles for *Dunkleosteus terrelli* (modeled after CMNH 7054), comparing reconstructed gape angles in Anderson (2010) (22°) and Anderson and Westneat (2009) (45°) with gape angle (65°) based on present muscle reconstructions and necessary gape to achieve clearance between the odontoids. Angle measured between occlusal blades of the posterior supragnathal and infragnathal with mandibular articulations as a vertex.

However, these models do not agree with the comparative and functional anatomy of *Dunkleosteus*. Perhaps most notably, under these models *Dunkleosteus* is almost incapable of passing food items between its odontoids. At gape angles of 45°, large individuals of *Dunkleosteus* (CMNH 5768) only achieve 10–15 cm of clearance between the tips of the symphyseal and supragnathal odontoids (Figure 18, the distance here being slightly larger [17 cm] due to the shorter odontoids in the individual). At gape angles of 22° this clearance is less than 5 cm. Anderson and Westneat (2009), pp. 263–264) and Anderson (2010) also note under their model the large nuchal gap between the skull roof and median dorsal plate in *Dunkleosteus* and other pachyosteomorphs has no effect on gape. Maximal gape in these models is limited by the adductor musculature long before the increased range of motion from the nuchal gap becomes biomechanically relevant. This can be seen in that Anderson's models Anderson (2010) suggest no difference in maximal gape between pachyosteomorphs and coccosteomorphs; reconstructed gape angles in that study between taxa with large nuchal gaps (*Dunkleosteus*, gape angle = 22.88°) and small/absent nuchal gaps (e.g., *Mcnamaraspis*, gape angle = 21.36°) are nearly identical. While the posterior embayment of the nuchal could also be explained as producing a longer lever arm for the *m. levator capitis major* (Anderson & Westneat, 2009; Miles, 1967), there is no other explanation for the complimentary anterior embayment on the median dorsal plate of the trunk armor.

Because of the shorter length of the *m. adductor mandibulae* and inferred presence of a median raphe, the muscle reconstructions presented here allow for gapes of at least 65–70°, closer to gape angles seen in extant sharks (Cooper et al., 2022; Ferry‐Graham, 1998; McNeil et al., 2016; Moyer et al., 2021) and extant predators with large caniniforms (felids; Slater & Van Valkenburgh, 2009). It also agrees with several features of *Dunkleosteus* suggesting a large gape, such as a more posteroventral location of the quadrate articulation relative to other arthrodires (Engelman, 2023b) and the low angle of the suborbital groove, and allows the nuchal gap to function during jaw opening. Gape angles beyond ~65° potentially result in the dorsal glenoid facet extending anteriorly beyond the detent process of the palatoquadrate and dislocating unless long axis rotation of the mandible occurs. A more precise calculation of gape angle in *Dunkleosteus* via biomechanical modeling (e.g., as in Dearden et al., 2023) is beyond the scope of this study.

The low angle of the fibers of the *m. adductor mandibulae* (based on the shape of the adductor fossa) and median raphe may also control for the issues of possible overstretch noted by Anderson and Westneat (2009). Because of the slanted orientation of these muscle bodies, the line of action can rotate as the jaws of *Dunkleosteus* open rather than be forced to stretch excessively as would be required if the longest fibers of the *m. adductor mandibulae* followed a direct, vertical path between their origin and insertion. At a gape angle of 65°, the arrangement in this reconstruction allows the dorsal and ventral heads of the *m. adductor mandibulae* to stretch a mere ~20% of its resting length (File S15), within the range of muscle stretch factors in other systems (10%–20%; Anderson & Westneat, 2009, p. 258). By contrast, a purely vertical line of action requires an impossible stretch factor of over 40%. Estimated stretch factors in the present reconstruction are at least partially constrained by challenges in accommodating the ventral head of the *m. adductor mandibulae*, which is less angled and has a shorter line of action. In the present model, the median raphe must rotate slightly downward to keep stretch factors for the ventral head within physiologically tolerable limits (File S15). By contrast, stretch factors for the dorsal head of the *m. adductor mandibulae*, assuming the median raphe remains horizontal, are only about 15% resting length. However, at the same time, the resting length of the ventral head is determined by the shape of the (reconstructed, unpreserved) adductor fossa, and thus these values could be overestimates. If the adductor fossa were deeper anteriorly, allowing a more angled line of action for the ventral head of the *m. adductor mandibulae*, as in the dorsal head, the stretch factor might be lower.

### 4.4 Evolutionary trends in jaw morphology in *Dunkleosteus* and other arthrodires

#### 4.4.1 Macropredatory specializations in eubrachythoracid arthrodires

The cranio‐mandibular anatomy of *Dunkleosteus* has been primarily examined from a mechanical perspective and biomechanical modeling (Anderson & Westneat, 2007, 2009; Heintz, 1932; Miles, 1969; Snively et al., 2010). By contrast, observations based on the comparative anatomy of other arthrodires are more limited (see Anderson, 2008b, 2010; Miles, 1969, for exceptions). This restricts the degree to which information from *D. terrelli* can contribute to broader understandings of arthrodire paleoecology and evolutionary history. When examining the comparative anatomy of *Dunkleosteus*, two areas are of prime interest. First, how does the morphology of *Dunkleosteus* and other dunkleosteoids compare with the other major clade of macropredatory arthrodires within Pachyosteomorpha—macropredatory aspinothoracidans such as *Gorgonichthys*, *Dinichthys*, and *Heintzichthys?* These forms, despite being distantly related to dunkleosteoids (Boyle & Ryan, 2017; Carr & Hlavin, 1995, 2010; Zhu, Zhu, & Wang, 2016), have been noted to show substantial convergences in overall morphology (Anderson, 2008b, 2009; Carr & Hlavin, 1995, 2010; Denison, 1978). Second, how does the functional morphology of *Dunkleosteus* and its broader lineage (Dunkleosteoidea) compare to the generalized, ancestral eubrachythoracid condition seen in the outgroup to Pachyosteomorpha, represented by “coccosteomorph‐grade” arthrodires (sensu Miles, 1969). Using coccosteomorphs as models for the ancestral eubrachythoracid condition seems valid given the clade is primarily diagnosed by symplesiomorphies (Carr, 2004b), which are often shared with the basalmost members of Dunkleosteoidea and Aspinothoracidi (Carr, 2004b; Dennis & Miles, 1980; Dennis‐Bryan, 1987), and this group is often treated as a morphological “level of organization” from which various pachyosteomorph lineages have arisen (Carr, 2004b; Miles, 1969).

*Dunkleosteus* shows a number of apomorphic features relative to the ancestral eubrachythoracid condition. These apomorphies include the development of large odontoids, a general loss or de‐emphasis of denticle fields in favor of oral blades (see below), an enlarged mandibular arch with a posteriorly elongate postorbital cheek and asymmetrical adductor fossa, a greatly enlarged linguiform process and suborbital groove, an expanded contact for the ethmoid on the anterior supragnathal, a greatly enlarged lateral consolidated area that extends to the posterior margins of the head shield, a rod‐shaped submarginal plate, lack of contacts or articulations between the skull roof‐submarginal and submarginal‐suborbital/postorbital plates, a large nuchal gap, and a proportionally larger symphyseal articulation on the mentomandibular ossification. Some of these features have arisen independently in other arthodires (e.g., macropredatory aspinothoracidans, see below), but based on character distributions appear to represent character state transformation within Dunkleosteoidea from a coccosteomorph‐like ancestor (rather than, say, pachyosteomorph symplesiomorphies). Based on the soft‐tissue correlates of these features, many appear to reflect specializations for macropredation (i.e., feeding on large vertebrates), particularly enhancement of bite force and gape.

*Eastmanosteus calliaspis* is intermediate in morphology between *Dunkleosteus terrelli* and the outgroup condition in many respects (represented by coccosteomorphs [including plourdosteids] and basal/non‐macropredatory aspinothoracidans, which often share symplesiomorphies for many of these features), including the degree of elongation of the postorbital cheek and overall mouth size, degree of integration between head shield, submarginal, and suborbital/postsuborbital plates, size of the linguiform process (more similar to but slightly smaller than in *Dunkleosteus*) and the relative size of the odontoids on the anterior supragnathal (compare Figure 12a,b with Dennis‐Bryan, 1987, figs. 18a,b and 19a–d). In other cases, *E. calliaspis* differs from *Dunkleosteus* in retaining a number of symplesiomorphic features, including the absence of symphyseal odontoids on the infragnathal, relative size of the lateral consolidated area, small symphyseal articulation, lack of nuchal embayment, absence of expanded anterior supragnathal/ethmoid contact, and general patterns of denticulation on the gnathals (see below). In these features, the functional morphology of *E. calliaspis* more closely resembles coeval coccosteomorphs (e.g., *Torosteus*) than *D. terrelli*. Although *E. calliaspis* was capable of taking large prey, its morphology is clearly less specialized for a macropredatory niche than later members of its group. Anderson (2009) came to similar conclusions, recovering the functional morphology of the infragnathal in *E. calliaspis* as more similar to *Torosteus* or *Harrytoombsia* than *Dunkleosteus* or *Gorgonichthys* and interpreted *E. calliaspis* as a “facultative piscivore” (= facultative macropredator) similar to a reef shark rather than a dedicated macropredator. The morphology of other members of Dunkleosteoidea (*E. pustulosus*, “*Dunkleosteus*” *magnificus*, *Golshanichthys asiatica*, possibly *Westralichthys uwagedensis*), although not known in as much detail as *D. terrelli* or *E. calliaspis*, suggests an intermediate morphology or a mosaic acquisition of many traits (e.g., loss of symphyseal teeth and development of odontoids, enlargement of the lateral consolidated region) within this group. Overall, this suggests dunkleosteoids became increasingly specialized for feeding on large vertebrates over the course of their evolution.

**FIGURE 19:**
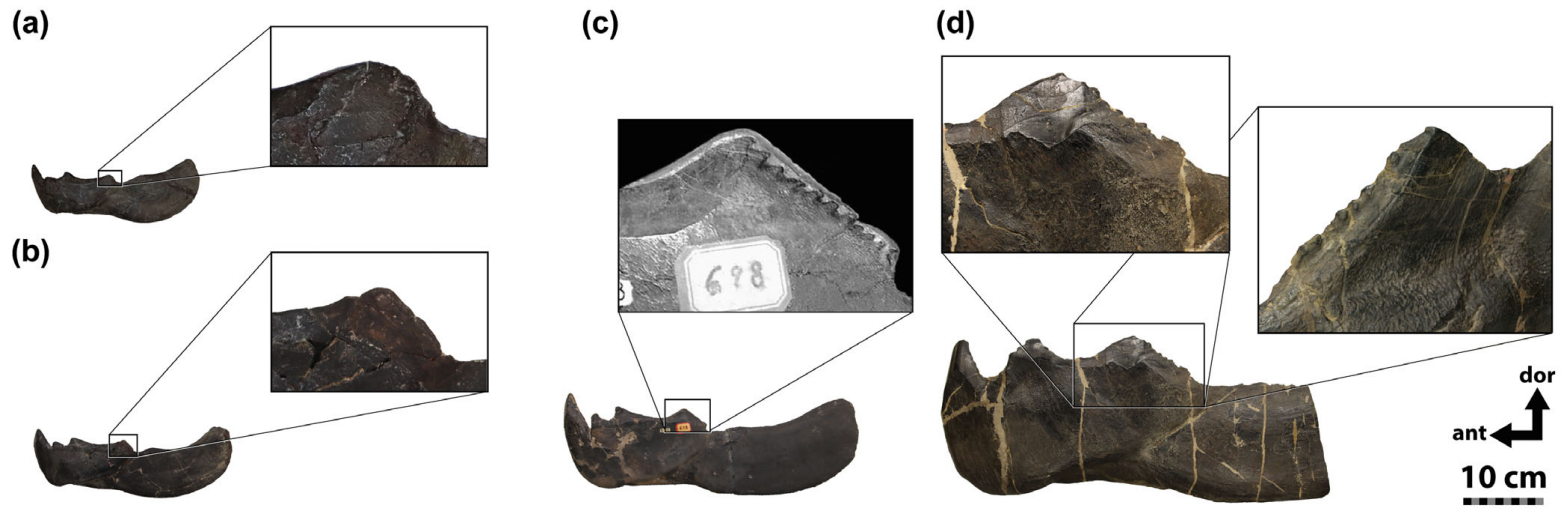
Infragnathals of *Dunkleosteus terrelli* in left lateral view, showing the individual variability in the presence of denticles. (a, b) CMNH 51774 (a) and CMNH 8808 (b), two small juveniles with no denticulation. (c) CMNH 5698, a medium‐sized individual with well‐preserved, fine denticulation. (d) CMNH 5936, the largest known *D. terrelli* infragnathal with irregular, well‐spaced denticles. Secondary inset image in (d) is in oblique posterointernal view, better showing the morphology of the marginal tooth row. Inset image in C is modified from Johanson and Smith (2005), used with permission. Scale is the same for all images outside of boxes.

Denticulation patterns in *Eastmanosteus calliaspis* are also roughly intermediate between *Dunkleosteus terrelli* and the outgroup condition, and in some cases are more similar to non‐dunkleosteoids. The gnathal plates of eubrachythoracid arthrodires are typically characterized by rows of statodont, acrodont teeth organized into a number of denticle fields (Johanson & Smith, 2005; Rücklin et al., 2012; Smith & Johanson, 2003). *Dunkleosteus* is unusual among arthrodires in having secondarily lost or reduced all of these denticle fields. This is not unique among eubrachythoracids but is uncommon even among macropredatory taxa, in which significant denticulation may be present (Carr, 1991; Carr & Hlavin, 2010). The only remaining teeth on the gnathal plates of *D. terrelli* are on the posterior face of the posterior corner of the infragnathal and the posterior edge of the posterior supragnathal (Johanson & Smith, 2005, fig. 6; Lebedev et al., 2023). Even on these elements their presence and morphology can be variable, in a significant portion of the CMNH hypodigm (CMNH 5007, CMNH 5959, CMNH 6020, CMNH 6090, CMNH 6194, CMNH 7069, CMNH 7568, CMNH 7714, CMNH 8793, CMNH 8808; Figures 1, 16, and 19a,b, Files S2 and S14) there are no identifiable teeth on the posterior corner of the infragnathal. The large number of specimens for which teeth are not present suggests their absence cannot be attributed to preparation damage. The morphology of these teeth is variable; in a few specimens the teeth are very fine and evenly spaced, giving a serrated appearance (CMNH 5698; Johanson & Smith, 2005, fig. 6b,e), whereas in others (CMNH 5300, CMNH 5936, and CMNH 7835) they may be low, rounded, and irregularly placed. There is no correlation between infragnathal size and tooth presence, as would be expected if tooth formation ceased late in ontogeny (Figure 19); some of the smallest specimens (CMNH 51774, CMNH 8808) lack teeth whereas the very largest specimen in the CMNH hypodigm (CMNH 5936) retains identifiable teeth (Engelman, 2023a, fig. 9; Lebedev et al., 2023, p. 8; Engelman, personal observation 2022). However, at the same time, medium (CMNH 6090) and large (CMNH 5768) specimens may still lack marginal teeth.

Other tooth fields seen in eubrachythoracids, including other dunkleosteoids like *Eastmanosteus calliaspis*, are absent. In *E. calliaspis*, the occlusal edge of the infragnathal often bears a number of teeth (Dennis‐Bryan, 1987, p. 32, fig. 18f,g). These structures are gradually worn down throughout ontogeny to produce an oral blade, though new teeth may be added to the oral surface posteriorly (Johanson & Smith, 2005). This appears to be a common pattern across arthrodires with bladed dentitions (Carr, 1991, p. 360, fig. 7; Johanson & Smith, 2005; Rücklin et al., 2012). By contrast, the biting surfaces of the gnathal plates in *Dunkleosteus terrelli* are completely bladed, even in juveniles (e.g., AMNH FF 7873, CMNH 6194, CMNH 8982, CMNH 51774; File S14), and functional teeth are absent. Similar patterns are present in other denticle fields. *D. terrelli* completely lacks any trace of adsymphyseal teeth at any stage of ontogeny, whereas in *E. calliaspis* symphyseal teeth continue to be produced even in old individuals (Dennis‐Bryan, 1987, p. 32). *Eastmanosteus* also shows a ventral and lateral tooth field on the posterior supragnathal, at least initially (Dennis‐Bryan, 1987), resembling the more broadly distributed condition in arthrodires (Gardiner & Miles, 1994; Miles & Dennis, 1979). By contrast, in *Dunkleosteus* the posterior supragnathal forms a single blade with limited denticulation on its posterior edge (Johanson & Smith, 2005, fig. 6a,c), resembling the condition in the infragnathal. Based on their location, the teeth on the posterior supragnathal appear to be homologous with the ventral tooth field of other arthrodires, with no trace that a lateral field was ever present in *D. terrelli*, even in the smallest individuals in the CMNH hypodigm (CMNH 6194, CMNH 8982; File S7C). Similarly, teeth are present on the anterior supragnathal in early ontogenetic stages of *E. calliaspis* (Dennis‐Bryan, 1987), whereas they are absent even in the earliest stages available for *D. terrelli* (CMNH 6194, File S7A,B). These differences do not appear attributable to differences in ontogenetic sampling. The size disparity between the largest and smallest individuals of *D. terrelli* in the Cleveland Shale hypodigm is similar to that of *E. calliaspis* at Gogo (smallest are about ¼ the size of the largest in linear dimensions; Dennis‐Bryan, 1987; R. Engelman personal observation, 2022), suggesting at least some overlap in the ontogenetic stages sampled.

Returning to the teeth on the posterior faces of the infragnathal and posterior supragnathal, these teeth are located in regions that do not occlude and are so small and fine compared to other arthrodires (including *Eastmanosteus calliaspis*) that it suggests they were non‐functional, as noted above. Their variable presence and morphology further suggest these teeth were likely vestigial. The possibility remains that these structures could have helped moderate formation of the oral blades through contribution of pleuromeric dentin (Johanson & Smith, 2005), but their absence in many specimens of *Dunkleosteus* suggests their function as teeth was limited and the presence of marginal teeth was not critical for survival. This suggests ontogenetic predisplacement has occurred in *D. terrelli* relative to *E. calliaspis*, with the factors promoting the formation of gnathal blades shifted so early in ontogeny that the initial tooth‐bearing stage only occurs in extremely young individuals of *D. terrelli*, if at all. In some cases, like the adsymphyseal teeth, whose presence or absence is not ontogenetically variable in *Eastmanosteus* (indeed, the number of adsymphyseal teeth tends to increase with age as more are added from the ventral end of the tooth row; Dennis‐Bryan, 1987, p. 32), these dental fields are better interpreted as having been outright lost in *D. terrelli*.

Denticulation patterns in other dunkleosteoids are less clear due to the paucity of gnathal plates, but the condition in taxa like *Eastmanosteus pustulosus* and “*Dunkleosteus” magnificus* (Hussakof & Bryant, 1918) does seem to suggest a progressive loss of the symphyseal dentition and other dental fields alongside the development of the symphyseal odontoid and the emphasis of oral blades. This, again, agrees with the overall interpretation presented here of increasing specializations for macropredation across evolutionary time in Dunkleosteoidea; mouthparts with prominent teeth are better suited for seizing smaller prey whereas odontoids and oral blades are expected to perform better at seizing and processing large prey items greater than the width of the mouth.

Many of these apomorphic features seen in *Dunkleosteus* and/or Dunkleosteoidea appear to have evolved in parallel in macropredatory aspinothoracidans. These include a posteriorly extensive lateral consolidated area, a well‐developed nuchal gap, a larger mouth and elongated postorbital cheek, expanded contact between the ethmoid ossification and anterior supragnathal, increased infragnathal contribution to the mandible (see below), loss of adsymphyseal teeth, development of symphyseal odontoids, and dentitions emphasizing the function of oral blades and odontoids over grasping teeth or crushing surfaces. Some of these convergences broadly resemble specializations for macropredatory behavior patterns seen in other vertebrates like sharks and carnivorous mammals, including a larger mouth and mandibular arch, a larger gape, development of caniniform structures, bladed dentition specialized for cutting, and features related to producing a more resilient mandible.

Similar observations have been made by other authors; Denison (1978) considers the presence of symphyseal odontoids and large, elongate, and loosely attached cheek units as diagnostic for his “Dinichthyidae,” which is now considered to represent a polyphyletic assemblage of dunkleosteoids and macropredatory aspinothoracidans (Carr & Hlavin, 1995, 2010). Similarly, the functional analysis of Anderson (2009) finds *D. terrelli* to group closer to the macropredatory aspinothoracidans *Gorgonichthys* and *Heintzichthys* than the fellow dunkleosteoid *Eastmanosteus*. Overall, this suggests the two main clades of macropredatory arthrodires evolved a significant number of convergent specializations for taking large prey. More research is needed on macropredatory aspinothoracidans (especially their phylogeny) to determine if these taxa exhibit a progressive specialization over time as suggested by dunkleosteoid evolution.

#### 4.4.2 Evolution of the mandible and jaw cartilages within Eubrachythoraci

Two broader conclusions about jaw evolution in arthrodires are evident from the present study: (1) the bony component of the mandible (infragnathal) in *Dunkleosteus* was proportionally larger than in many other arthrodires, and (2) despite this, much of the mandible—including the location of most major muscle attachments and articulation sites—remained cartilaginous. Arthrodires show a clear, progressive trend toward jaw ossification and simplification over evolutionary time (previously discussed by Young, 2010). The mandibles of the earliest arthrodires (non‐eubrachythoracids like *Dicksonosteus*, buchanosteids, *Holonema*; Figure 17a,b) are almost completely composed of an undifferentiated, often unossified Meckel's cartilage, with the infragnathal restricted to a thin tooth‐bearing region near the mouth and (sometimes) a slight posterior lamina homologous to the adductor lamina of later‐diverging taxa (Goujet, 1984; Hu et al., 2017; Lelièvre, 1984; Miles & White, 1971; Young et al., 2001). In ANU V244 the entire Meckel's cartilage is perichondrally ossified, but this is a condition currently unique to this specimen among non‐eubrachythoracids (Hu et al., 2017, p. 4; Young et al., 2001, p. 674) and in other taxa the Meckel's cartilage is considered to be unossified or poorly mineralized. There is no obvious symphysis between the two mandibles; instead, the connection between the two hemimandibles appears to be ligamentous as in elasmobranchs. Hu et al. (2017) suggested an additional, unpreserved basimandibular element was present between the two mandibles, but the similarity of this morphology to elasmobranchs (both have anteriorly narrow mandibles with no well‐developed symphysis) suggests an elasmobranch‐like arrangement might be more likely.

By contrast, in coccosteomorphs (Figure 17c,d), the infragnathal has expanded to become a clear “jaw‐like” structure serving as an internal support for the entire mandible, with the infragnathal extending from the occlusal margin to the level of the articular cartilage (Carr, 1995, p. 103). The infragnathal is also much deeper relative to mandible length than the thin element seen in most non‐eubrachythoracids, though the adductor lamina is often deeper than the oral region in many forms (Anderson, 2008b; see also figures in Dennis & Miles, 1979a; Dennis & Miles, 1979b; Gardiner & Miles, 1994; Miles & Dennis, 1979). In coccosteomorphs, the anterior and posterior ends of the Meckelian cartilage have perichondrally ossified into the mentomandibular element and the articular ossification (Johanson, 2003, figs. 4 and 5; Miles & Westoll, 1968), further reducing the proportion of unmineralized cartilage in the mandible, but the remaining unossified (intermediate) Meckelian cartilage is still very large (about the same size as the infragnathal). The mentomandibular ossifications form a distinct symphysis; the ligamentous connection, as seen in buchanosteids, remains but forms a foramen near or within the symphyseal face of the mentomandibular ossification.

Macropredatory pachyosteomorphs take this trend even further. In *Dunkleosteus* (Figure 17e,f), the infragnathal represents a larger proportion of the total mandible than in *Eastmanosteus* or coccosteomorphs, such that the majority of the jaw is composed of this bony element. The infragnathal is also much deeper relative to mandible length, especially at the oral region (Figure 17e). The adductor lamina is proportionally larger than the intermediate Meckelian cartilage, even though most of the *m. adductor mandibulae* still inserts on the latter element. Additionally, more of the infragnathal is exposed and thus available as a site for muscle attachment. The mentomandibular ossification is comparatively reduced, despite this element still supporting a proportionally larger symphyseal articulation. In *Eastmanosteus* and most coccosteomorphs, the mentomandibular element is relatively large compared to the oral region of the infragnathal and is visible in external view. However, in *Dunkleosteus*, this element is proportionally smaller, to the point that the mentomandibular ossification is not visible externally in life position. At least some of this may be a consequence of the greater height of the oral region of the infragnathal.

Whether similar trends occur in aspinothoracidans is unclear. The infragnathals of the macropredatory aspinothoracidans *Dinichthys* (Hussakof, 1906), *Gorgonichthys* (CMNH 7129), and *Holdenius* (Dunkle & Bungart, 1942b) resemble *Dunkleosteus* in having relatively deep infragnathals, particularly in the oral region, though *Heintzichthys* is an outlier among these forms in having a very shallow infragnathal relative to mandible length (Anderson, 2008b; Carr, 1991). Other aspinothoracidans like selenosteids tend to have relatively gracile infragnathals (Carr, 1994, 1996; Dunkle & Bungart, 1943; Jobbins et al., 2022; Stensiö, 1963), though in many of these forms the oral regions are often approximately the same height as the adductor lamina (unlike many coccosteomorphs). Macropredatory aspinothoracidans like *Heintzichthys* and *Gorgonichthys* also appear to resemble *Dunkleosteus* in having infragnathals with a reduced area of contact for the intermediate Meckelian cartilage and small articular facets for the mentomandibular ossification, though the morphology of the latter element is unknown. Compared to *Dunkleosteus*, *Gorgonichthys* (CMNH 7129) actually shows a comparatively larger and more prominent attachment site for the mentomandibular ossification on the internal face of the infragnathal at the level of the mandibular symphysis, which could indicate a relatively robust, well‐developed mandibular symphysis in *Gorgonichthys*. The internal attachment facet is very small on the infragnathal of *Heintzichthys* (CMNH 6025), fitting with the overall gracile nature of this taxon's mandible. Other aspinothoracidans like *Diplognathus* (Dunkle & Bungart, 1943), *Kendrickichthys* (Dennis & Miles, 1980, fig. 21), *Tapinosteus*, and *Pholidosteus* (Stensiö, 1963, pp. 243–244, fig. 80) seem to have more extensive cartilages similar to coccosteomorphs or intermediate between the condition in coccosteomorphs and *Dunkleosteus*, whereas *Paramylostoma* has an extremely small mentomandibular ossification fitting with the overall gracile nature of this taxon's mandible (Dunkle & Bungart, 1945, fig.e 2, AIG). The morphology of the Meckelian cartilages and their articulations with the infragnathal across arthrodires needs more extensive analysis.

A roughly analogous pattern to what is observed in arthrodires can be seen in synapsid tetrapods, where over the course of evolutionary time most of the elements of the mandible are gradually reduced in favor of a greatly expanded dentary (File S16), which increasingly comes to dominate and serve as the core structural support of the mandible (Kemp, 2005). Broader patterns in shape evolution are also similar (File S16), both start as a bony lamina supporting the lower dentition but subsequently expand to the level of the cranio‐mandibular articulation, displacing many of the other mandibular elements (Carr, 1995; Goujet, 1984; Kemp, 2005; Young, 2010). Indeed, the two elements primarily involved in these patterns, the infragnathal and dentary, may be homologous (Zhu, Ahlberg, et al., 2016). Additionally, in synapsids much of this increase in the size of the dentary is driven by a vertical expansion in dentary height relative to mandible length (Harano & Asahara, 2023), resembling the pattern in arthrodires (File S16A–C). In synapsids, this simplification of the jaw and expansion of the dentary results in a more resilient (stiffer) mandible than in other vertebrates (Tseng et al., 2023) and has been suggested to allow for higher bite force and a jaw more capable of resisting the repeated loads of mastication (Kemp, 2005; but see Lautenschlager et al., 2018, in part). Similar patterns toward increased simplification of the jaw and reduction of the Meckelian cartilages are present in other vertebrates, but the biomechanical consequences of this pattern have not been examined in as much detail (Watt et al., 2024). The reduction of the jaw cartilages and perichondral ossification of the anterior (mentomandibular) and posterior (articular) ends of the Meckelian cartilage would likely have a similar effect, producing a stiffer and more robust lower jaw.

In the case of arthrodires, this increase in the robustness of the mandible is likely related to higher bite forces and the ability to handle larger and more resilient prey. Patterns of mandible ossification correlate fairly well with previous broad‐scale interpretations of dietary habits across arthrodire evolution. Non‐eubrachythoracids are often interpreted as being microphagous, detritivorous, or feeding on small and/or soft‐bodied organisms (Denison, 1978; Dupret, 2010; Fitzpatrick et al., 2024; Gess & Whitfield, 2020, p. 872; Lebedev et al., 2009; Miles & White, 1971; Trinajstic, Briggs, & Long, 2022), with the exception of phyllolepids, which have been interpreted as benthic, macropredatory suction feeders akin to squatinids (Carr et al., 2009; Ritchie, 2005). Coccosteomorphs and similar forms, with their proportionally larger infragnathals, have been interpreted as generalist predators of invertebrates and fishes (Miles & Westoll, 1968; Trewin, 1986; Trinajstic, Long, et al., 2022), though camuropiscids, with their relatively more gracile infragnathals, have been suggested to feed on soft‐bodied or small prey (Anderson, 2009). Finally, the arthrodires with the proportionally largest infragnathals (dunkleosteoids and macropredatory aspinothoracidans) have been interpreted as specialists on large vertebrates (Anderson, 2009; Anderson & Westneat, 2009; Hall et al., 2016; Hlavin, 1990; Hussakof, 1906; Hussakof & Bryant, 1918; Miles, 1969). This interpretation agrees well with similar suggestions of high bite force in *Dunkleosteus* (Anderson & Westneat, 2007, 2009; Miles, 1969; Snively et al., 2010). Anderson (2008b) actually suggested the opposite pattern, with the mandible of coccosteomorphs being stiffer and more resilient than in pachyosteomorphs due to their proportionally deeper adductor lamina compared to the oral region. However, this study only looked at the infragnathal and did not consider the effect of a proportionally greater amount of the coccosteomorph mandible being composed of cartilage, which may be the source of this discrepancy. Additional variation exists within these broad categories beyond the overview presented here; for example, Anderson (2009) recovered *Harrytoombsia*, *Torosteus*, and *E. calliaspis* as “facultative piscivores” which would imply a condition roughly intermediate between that seen in typical coccosteomorphs and *Dunkleosteus* as defined here. However, reviewing this variation in detail is beyond the scope of this study; we merely define the general pattern here to provide a model to better understand broad‐scale patterns in arthrodire jaw evolution.

Nevertheless, despite the mandibular cartilages of *Dunkleosteus* being relatively small, it is clear that *Dunkleosteus* resembled other arthrodires in a significant, functionally non‐negligible portion of the mandible being composed of cartilage. Previous authors tended to assume the Meckelian cartilages of *Dunkleosteus*, particularly the intermediate Meckelian cartilage, were highly reduced to absent and played minor roles in jaw function (Anderson, 2008b; Anderson & Westneat, 2007, 2009; Heintz, 1932; Hussakof, 1906, p. 136; Snively et al., 2010). Even Young (2010, p. 532), who recognized the mentomandibular ossification and articular cartilage of *Dunkleosteus* as present, considered the intermediate Meckelian cartilage to have been lost. These assumptions, in turn, influenced functional models of this taxon (Anderson & Westneat, 2007, 2009; Snively et al., 2010). However, this work and its preceding study (Lebedev et al., 2023) identified several features suggesting an intermediate Meckelian cartilage was present, including attachment sites on the mentomandibular ossification and articular, a well‐developed Meckelian groove on the ventral surface of the infragnathal, and a large, well‐denoted contact face for this element on the ventral half of the adductor lamina (Lebedev et al., 2023, fig. 4). The presence of an intermediate Meckelian cartilage is further implied by the absence of an adductor fossa on the other elements of the mandible. If the intermediate cartilage was absent, this would imply the mandible of eubrachythoracids lacked an adductor fossa for the *m. adductor mandibulae*, a highly unusual condition among gnathostomes, despite being present in non‐eubrachythoracid arthrodires (Hu et al., 2017; Young et al., 2001). Thus, while the cartilaginous components of the mandible are reduced relative to other arthrodires, they still played a significant role in jaw function.

Similarly, despite the proportionally larger contribution of the infragnathal to the mandible of *Dunkleosteus*, articulations between the lower jaw and the cranium or at the mandibular symphysis were still retained on the relatively small, perichondrally ossified Meckelian cartilages. The cartilages were never reduced to the point these articulations migrated onto the infragnathal itself (though this partially occurred in some camuropiscids; Dennis & Miles, 1979a, p. 311), nor did the mentomandibular ossification or articular cartilage fuse with the infragnathal into a single element. Much, if not the majority, of the *m. adductor mandibulae* in *Dunkleosteus* still inserted onto the unossified intermediate Meckelian cartilage, though a greater proportion appears to have inserted onto the infragnathal than in coccosteomorphs or *Eastmanosteus*. Despite the relatively large size of the adductor lamina, its functional interactions with elements outside of the mandible were limited. Thus, it appears the primary function of the expanded adductor lamina in *Dunkleosteus* was to serve as a bony core providing support for the rest of the lower jaw, in contrast to many other vertebrates where many of the articulations and muscle attachment sites are located directly on the bony mandible.

## 5 CONCLUSIONS

Despite having been studied by many different authors, the functional morphology of the jaws of *Dunkleosteus* remains poorly understood. Many aspects of this taxon's morphology, such as the jaw cartilages, submarginal plate, and suborbital groove, were not considered in previous analyses, and their effects on previous biomechanical models may be significant. The functional significance of some features can be explained by biomechanics and comparative anatomy (e.g., the association between the suborbital groove and *m. preorbitalis*), but others, such as the submarginal plate, lateral consolidated region, and presence/absence of symphyseal teeth, remain poorly understood. The jaw anatomy of eubrachythoracid arthrodires is broadly similar to other gnathostomes; previous suggestions of a radically different arrangement of jaw muscles (e.g., the entire *m. adductor mandibulae* inserting on the head shield) are untenable.

Ironically, despite often being treated as the quintessential arthrodire by non‐specialists (e.g., see discussion in Miyashita, 2024, p. 440), the cranio‐mandibular anatomy of *Dunkleosteus* is highly atypical even among eubrachythoracids, including in the near absence of teeth (Johanson & Smith, 2005), robust mandibular symphysis, and little to no long axis rotation during jaw movement, elongate submarginal plate/hyomandibula, and extremely loosely integrated head shield/submarginal plate/cheek unit (especially posteriorly), and proportionally larger lateral consolidated region on the internal headshield. This degree of specialization is more likely a reflection of the poorer state of knowledge of dunkleosteoid morphology compared to coccosteomorphs or aspinothoracidans rather than *Dunkleosteus* being a complete outlier. Aside from *D. marsaisi* (which may be conspecific with *D. terrelli*; Boyle, 2025; Rücklin, 2002), the only dunkleosteoid known from near‐complete, well‐described remains is *Eastmanosteus calliaspis*, whose morphology is either plesiomorphic or shows a less extreme degree of specialization compared to *D. terrelli*. Other taxa like *E. pustulosus* and “*D.” magnificus* are known from a decent amount of material (Eastman, 1898; Hussakof & Bryant, 1918), but are in need of formal redescription. In many dunkleosteoids, the cheek plates and/or gnathals are unknown (as in *“D.” magnificus* [gnathals but no cheek plates], *“D.” newberryi* [gnathals but no cheek plates], *D. raveri* [both unknown], *D. amblyodoratus* [both unknown], *Kiangyousteus yohii* [only the anterior supragnathal is known], *Westralichthys uwagedensis* [both unknown], and *Golshanichthys asiatica* [infragnathal but no cheek plates or supragnathals]), meaning their condition cannot be evaluated (Carr & Hlavin, 2010; Hussakof & Bryant, 1918; Lelièvre et al., 1981; Long, 1987; Zhu & Zhu, 2013). It is highly likely that many of the supposedly “autapomorphic” traits identified in *Dunkleosteus* here are actually more broadly distributed within the group and simply have yet to be identified due to lack of material and/or analysis. Some of the characters identified here may be relevant for phylogenetic analyses. More extensive comparative morphological studies in other arthrodires are needed to elucidate the functional morphology of the jaw apparatus in *Dunkleosteus* and other specialized latest Devonian forms.

Arthrodires seem to show much more variation in their jaw structure than previously assumed, and some of the features seen in *Dunkleosteus* seem to support previous paleobiological interpretations of macropredation on large vertebrates (e.g., large gape, orthal jaw movement with a robust mandibular symphysis). Arthrodire evolutionary history has often been framed in terms of a broad‐scope, seemingly unidirectional pattern toward reduced armor and more active nektonic habits over geologic time (Carr, 1995; Denison, 1978; Engelman, 2024; Miles, 1969; Stensiö, 1963; Young, 2010). However, the present study highlights the underlying complexity within that pattern—many lineages within Arthrodira exhibit their own distinct evolutionary patterns of specialization related to particular life habits, and *Dunkleosteus* cannot be treated as an idealized exemplar or teleological endpoint for Pachyosteomorpha. Many of the features identified in this study (morphology and size of the suborbital groove, degree of cheek/skull roof integration, size and morphology of the lateral consolidated region) show significant interspecific variation across eubrachythoracid arthrodires. While in‐depth evaluation of this variation is beyond the scope of this study, its presence suggests that further study of these features might provide useful insights into the phylogeny, functional anatomy, and paleobiology of this group. Furthermore, the evaluation of the comparative anatomy of *Dunkleosteus* relative to an ancestral, generalized coccosteomorph‐like bauplan creates a framework in which the functional morphology of other highly specialized eubrachythoracids (e.g., *Alienacanthus*, Camuropiscidae, *Mylostoma*, *Titanichthys*; Coatham et al., 2020; Dennis & Miles, 1979a; Hlavin & Boreske, 1973; Jobbins et al., 2024) can be better understood. Arguments for suction feeding in *Dunkleosteus* based on biomechanical models are tenuous on the grounds of functional morphology and paleoecology and need to be revisited with more complex (i.e., three‐dimensional) biomechanical models.

## AUTHOR CONTRIBUTIONS

**Russell K. Engelman:** Conceptualization; methodology; data curation; investigation; validation; formal analysis; visualization; project administration; writing – original draft; writing – review and editing; supervision; resources. **Robert K. Carr:** Validation; investigation; formal analysis; visualization; writing – review and editing; resources; writing – original draft. **Kate Trinajstic:** Funding acquisition; resources; writing – review and editing; validation; writing – original draft; methodology. **Zerina Johanson:** Conceptualization; methodology; investigation; validation; formal analysis; supervision; writing – original draft; writing – review and editing; project administration. **Oleg A. Lebedev:** Conceptualization; writing – original draft; writing – review and editing; methodology; investigation; validation.

## FUNDING INFORMATION

Digital models used in the course of this study were created using funds from NSF DBI 2230809 to C. Colleary, A. McGee, H. Majewski, D. Donovan, and the CMNH to create publicly available 3D models. K. Trinajstic was funded through ARC DP220100825 and DP240102156.

## CONFLICT OF INTEREST STATEMENT

The authors declare they have no conflict of interest.

## Supporting information


**Data S1.** Comparison of cheek units of *Eastmanosteus calliaspis* (ANU 21633, A) and *Dunkleosteus terrelli* (CMNH 6201, B), showing how cheek unit of B is more elongate and the deepest point of the adductor fossa (in pink) is located more anteriorly. (A) from Hu et al. (2017), postsuborbital plate in (B) restored after Heintz (1932) and observations of CMNH 7424.

**Data S2.** Photograph (A) and line drawing (B) of a right infragnathal of *Dunkleosteus terrelli* with the articular in situ (CMNH 5768), showing the articulation for the unossified intermediate Meckelian cartilage on the articular, how the edge of the articular is continuous with the contact of the intermediate Meckelian cartilage on the infragnathal, and how the ridge on the articular (r.1) seemingly suggests a separation between the muscle bodies inserting on the bony dorsal and cartilaginous ventral surfaces of the mandible. Abbreviations follow Figures 1 and 3.

**Data S3.** Variation in submarginal plate morphology in eubrachythoracids, showing two generalized arthrodires (A, B), the early dunkleosteoid *Eastmanosteus* (C), *Dunkleosteus* (D), and representatives of two groups (Camuropiscidae and Selenosteidae) that independently evolved completely consolidated cheek plates (E, F). (A) *Coccosteus cuspidatus*, modified from Engelman (2024); (B) *Compagopiscis croucheri*, modified from Gardiner and Miles (1994) and WAM 70.4.263; (C) *Eastmanosteus calliaspis*, modified from Dennis‐Bryan (1987); (D) *Dunkleosteus terrelli*, drawn from CMNH 7054; (E) *Rolfosteus canningensis*, modified from Dennis and Miles (1979a); (F) *Brachyosteus dietrichi*, modified from Gross (1932) and Stensiö (1963). Submarginal plate highlighted in yellow. Figures are scaled to approximately the same head length (not counting the elongate rostral plate of E).

**Data S4.** Relative proportions of the cheek unit in arthrodires. Methods of measuring the cheek unit follow Miles and Dennis (1979) (see File S17A).

**Data S5.** Graphs of (A) relative total cheek length versus postorbital cheek length as a proportion of total cheek length and (B) relative postorbital cheek length versus relative total cheek length (scaled by skull roof length) in eubrachythoracid arthrodires. Morphospaces occupied by smaller juveniles and larger subadults/adults of *Dunkleosteus terrelli* circled to show the differences in cheek unit proportion across ontogeny. Data in these graphs comes from File S4.

**Data S6.** Graphs of (A) skull roof length versus total cheek unit length, (B) postorbital cheek length versus skull roof lengths, and (C) total cheek length versus postorbital cheek length in eubrachythoracid arthrodires. Dashed line represents isometric slope of 1, with intercept set to minimum predicted value of best fit line to better show allometric patterns in measurements of interest. Data in these graphs from File S4.

**Data S7.** Supragnathals of CMNH 6194, one of the smallest individuals of *Dunkleosteus terrelli* in the CMNH hypodigm. (A, B) Anterior supragnathals in anterior (A) and lateral (B) views, showing the development of the descending “lip” for the contact with the ethmoid ossification and the absence of teeth. (C) Right posterior supragnathal in lateral view, showing the absence of a lateral denticle field, even as worn remnants. Although not a neonate, CMNH 6914 is a sufficiently small juvenile (~1/3 the size of the largest known specimens of *D. terrelli*) to show these features are consistent across ontogeny.

**Data S8.** Head shields of *Rolfosteus* (A, from Dennis & Miles, 1979a), *Mcnamaraspis* (B, from Long, 1995), *Dunkleosteus* (C, CMNH 7639, reversed), and *Gorgonichthys* (D, from Dunkle & Bungart, 1940) in ventral view, showing the interspecific variation in the size and morphology of the lateral consolidated region (highlighted in green).

**Data S9.** Relative size of the lateral consolidated region in arthrodires as a percent area of the surface of the head shield in ventral view. Measurements taken as 1/2 total skull area or the better preserved of the two lateral consolidated areas, with the average area of the lateral consolidated region taken when possible (File S17B).

**Data S10.** Right (A, C, E, G–I) and left (B, D, F, H) submarginal plates of an individual of *Dunkleosteus terrelli* (CMNH 6194), showing the morphology of this plate and the individual variability of its (vestigial?) contact with the skull roof. (A, B) and (E, F) are photographs, the remainder are 3D surface scans. (A–D) External views; (E–H) internal views, (H, I) cross‐sections of the right submarginal plate with the viewer looking mesially (H) and distally (I).

**Data S11.** Diagram showing the spectrum of posterior cheek plate integration in eubrachythoracids, showing how posterior cheek consolidation correlates with lateral consolidated region development. Sources for drawings as in File S3.

**Data S12.** Relative depth of the nuchal embayment in eubrachythoracid arthrodires. Methods follow File S17C.

**Data S13.** Ancestral character state reconstruction showing distribution of adsymphyseal teeth in Eubrachythoraci. Phylogeny and codings modified from a composite of Zhu, Zhu, and Wang (2016) and Boyle and Ryan (2017), see File S18 for more information.

**Data S14.** Antagonistic sets of posterior supragnathals (in internal view, reversed) and infragnathals in small (A, CMNH 6194), moderately large (B, CMNH 7054), and large (C, CMNH 5768) individuals, showing how wear rates are roughly consistent across the occlusal blade.

**Data S15.** Skull of *Dunkleosteus* with jaws closed (A) and at maximum (~65°) gape (B), showing the median raphe and the line of action for the *m. adductor mandibulae*. In B, the more angled orientation of the *m. adductor mandibulae* at rest compared to previous studies allows the jaws to open while only stretching the dorsal and ventral heads of this muscle by ~20% resting length.

**Data S16.** Lower jaws of arthrodires (left) and synapsids (right), showing convergent trends in increasing consolidation of the jaw, expansion of the primary oral element (dentary/infragnathal, shown in green), and reduction of other elements. (A–C) as in Figure 17b,d,f. (D) *Dimetrodon* from Romer and Price (1940); (E) *Thrinaxodon* from Luo and Manley (2020); (F) *Didelphis* modified from Case Western Reserve University mammal skull identification guide (CMNH 21719). Note that arthrodires never reach a mandible state as extreme as (F), where the dentary takes over all feeding function.

**Data S17.** (A) How the cheek unit was measured in File S4 (methods follow Miles & Dennis, 1979); (B) how the relative size of the lateral consolidated region was measured in File S9; (C) how the depth of the nuchal embayment was measured for File S12.

**Data S18.** Codings and data for tree in File S13.
