# Supplementary material for: Functional anatomy, jaw mechanisms, and feeding behavior of Dunkleosteus terrelli (Placodermi, Arthrodira)
Source: Anat Rec (Hoboken). 2025 Nov 20;309(10):2574–635. doi: 10.1002/ar.70075 (PMC13549283; doi:10.1002/ar.70075)
Supplement: Supplementary file 1 [file AR-309-2574-s016.docx]

# Institutional Abbreviations

**AMNH** – American Museum of Natural History, New York, USA

**ANU** – Australian National University, Canberra, Australia

**BMSC** – Buffalo Museum of Science, Buffalo, USA

**CMNH** – Cleveland Museum of Natural History, Cleveland, USA

**GPIMG** – Geowissenschaftliches Zentrum der Universität Göttingen, Göttingen, Germany

**IVPP** – Institute of Vertebrate Paleontology and Paleoanthropology, Beijing, China

**LACM** – Natural History Museum of Los Angeles County, Los Angeles, USA

**LDUCZ** – Grant Museum of Zoology, University College, London, U.K.

**MB.f** – fossil fish collections, Museum für Naturkunde, Berlin, Germany

**MCD** – Museum National d’Histoire Naturelle, Paris, France

**MCZ** – Museum of Comparative Zoology, Cambridge, USA

**MGSI** – Museum of the Geological Survey of Iran, Mehrabad-Téhéran, Iran

**MNHM** – Musée d’Histoire naturelle de Miguasha, Nouvelle, Québec, Canada

**NHMUK –** Musée d’Histoire Naturelle de Miguasha, Quebec, Canada

**NMS** – National Museum of Scotland, Edinburgh, U.K.

**NRM** – Swedish Museum of Natural History, Stockholm, Sweden

**PIMUZ –** Palaeontological Institute and Museum, University of Zurich, Zurich, Switzerland

**ROM** – Royal Ontario Museum, Toronto, Canada

**SMNK-PAL –** Staatliches Museum für Naturkunde Karlsruhe, Karlsruhe, Germany

**WAM** – Western Australian Museum, Perth, Australia

# Tree (In Newick Format)

('Holonema_westolli',((Antinosteus_lehmani,Homosteus_sulcatus),(('ANU Buchanosteid (ANU V244)',Goodradigbeeon_australianum),(Gemuendenaspis_angusta,((((Janiosteus_timanicus,Panxiosteus_ocullus,Plourdosteus_canadensis),((Millerosteus_minor,Coccosteus_cuspidatus),(Dickosteus_threiplandi,Watsonosteus_fletti,Protitanichthys_rockportensis))),(Harrytoombsia_elegans,((Torosteus_pulchellus,Torosteus_tuberculatus),(Mcnamaraspis_kaprios,(Compagopiscis_croucheri,Trematosteus_fontanellus,((Incisoscutum_ritchiei,Incisoscutum_sarahae),(Rolfosteus_canningensis,(Tubonasus_lennardensis,(Fallacosteus_turneri,(Latocamurus_coulthardi,(Camuropiscis_connicus,Camuropiscis_laidlawi))))))))))),(Rhachiosteus_pterygiatus,((Eastmanosteus_calliaspis,((Eastmanosteus_pustulosus,Kiangyousteus_yohii),'"Dunkleosteus" magnificus',(Golshanichthys_asiatica,(Westralichthys_uwagedensis,((Dunkleosteus_raveri,Dunkleosteus_terrelli),(Dunkleosteus_amblyodoratus,(Heterostius_ingens,Yinosteus_major))))))),(Tapinosteus_heintzi,((Bullerichthys_fascidens,(Bruntonichthys_multidens,Kendrickichthys_cavernosus)),(Dinichthys_herzeri,(Hadrosteus_rapax,((Tafilalichthys_lavocati,(Bungartius_perissus,(Titanichthys_agassizi,Titanichthys_cf._T_clarki))),(Gorgonichthys_clarki,(Heintzichthys_gouldi,(Gymnotrachelus_hydei,Pachyosteus_bulla,Stenosteus_angustopectus,(Rhinosteus_parvulus,(Melanomontanosteus_occitanus,(Brachyosteus_dietrichi,Driscollaspis_pankowskiorsum))),(Selenosteus_brevis,(Walterosteus_spp.,(Draconichthys_elegans,Enseosteus_spp.))))))))))))))))));

# Character-State Matrix

Holonema_westolli	0

Antinosteus_lehmani	0

Homosteus_sulcatus	0

'ANU Buchanosteid (ANU V244)'	0

Goodradigbeeon_australianum	0

Gemuendenaspis_angusta	1

Millerosteus_minor	?

Coccosteus_cuspidatus	1

Dickosteus_threiplandi	?

Watsonosteus_fletti	?

Protitanichthys_rockportensis	1

Harrytoombsia_elegans	1

Torosteus_pulchellus	1

Torosteus_tuberculatus	1

Mcnamaraspis_kaprios	1

Compagopiscis_croucheri	1

Incisoscutum_ritchiei	1

Incisoscutum_sarahae	1

Camuropiscis_connicus	1

Camuropiscis_laidlawi	1

Latocamurus_coulthardi	0

Tubonasus_lennardensis	0

Rolfosteus_canningensis	0

Fallacosteus_turneri	0

Janiosteus_timanicus	?

Panxiosteus_ocullus	?

Plourdosteus_canadensis	1

Eastmanosteus_calliaspis	1

Eastmanosteus_pustulosus	1

Westralichthys_uwagedensis	?

Kiangyousteus_yohii	?

Golshanichthys_asiatica	0

'"Dunkleosteus" magnificus'	1

Dunkleosteus_amblyodoratus	?

Dunkleosteus_raveri	?

Dunkleosteus_terrelli	0

Heterostius_ingens	?

Yinosteus_major	?

Rhachiosteus_pterygiatus	?

Tapinosteus_heintzi	?

Trematosteus_fontanellus	?

Bruntonichthys_multidens	1

Kendrickichthys_cavernosus	0

Bullerichthys_fascidens	1

Bungartius_perissus	0

Tafilalichthys_lavocati	?

Titanichthys_agassizi	0

Titanichthys_cf._T_clarki	0

Dinichthys_herzeri	1

Hadrosteus_rapax	?

Gorgonichthys_clarki	0

Heintzichthys_gouldi	0

Gymnotrachelus_hydei	1

Brachyosteus_dietrichi	0

Draconichthys_elegans	1

Driscollaspis_pankowskiorsum	?

Enseosteus_spp.	1

Melanomontanosteus_occitanus	1

Pachyosteus_bulla	?

Rhinosteus_parvulus	1

Selenosteus_brevis	1

Stenosteus_angustopectus	1

Walterosteus_spp.	?

# Character State Changes

*Homosteus sulcatus* – Coded as “absent” following Heintz (1933).

*Holonema westolli* – Coded as “absent” following Miles and White (1971).

*Gemuendenaspis angusta* – Coded as “present” following Miles (1962).

*Watsonosteus fletti* – Changed from “absent” to “unknown”. Miles and Westoll (1963) describe the infragnathal in the specimen of *W. fletti* they examined as highly worn, especially in the symphyseal region (which might suggest symphyseal teeth), and their figures are ambiguous as to whether symphyseal teeth are present (Miles and Westoll 1963: fig. 5a). Thus, it is unclear if *W. fletti* may have had adsymphyseal teeth, or if they were present in this individual earlier in life and the symphyseal surfaces were worn down (contra other taxa where the denticles are coded as absent like *Dunkleosteus terrelli* where these faces are not worn). Analysis of additional *Watsonosteus* remains under description (Newman et al. 2021) may help answer this question.

*Tubonasus lennardensis* – Coded as “absent” following Dennis and Miles (1979a: fig. 13b)

*Rolfosteus canningensis* – Coded as “absent” following Dennis and Miles (1979a: fig. 9)

*Kiangyousteus yohii* – Changed from “absent” to “unknown”. As mentioned by Zhu and Zhu (2013), the infragnathal is unknown for *K. yohii*.

*Eastmanosteus pustulosus* – Coded as “present” following Hussakof and Bryant (1918: pl. 13.2).

*Hadrosteus rapax* – Changed from “absent” to “unknown”. Stensiö (1963) provides several plates figuring the holotype of *H. rapax* (MB.f.163), but the adsymphyseal face of the infragnathal appears to be still embedded in the matrix. This is the only specimen of *H. rapax* in the Museum für Naturkunde collections (F. Witzmann, pers. comm. 2024), and is likely the only known specimen of this species. Thus, it is not possible to confirm the presence of adsymphyseal denticles in this taxon. Given that adsymphyseal denticles in other macropredatory aspinothoracidans like *Dinichthys herzeri* and *Eastmanosteus pustulosus* are very small structures located along the adsymphyseal face of the symphyseal odontoid and thus can be very easily overlooked (Hussakof 1906), we tentatively code *H. rapax* as “unknown” until its infragnathal can be re-examined in more detail.

*Melanomontanosteus occitans* – Changed from “absent” to “present”. Lelièvre et al. (1978: p. 13) remarks that the medial region of infragnathal of *M. occitans* “bears a vertical row of denticles, the most dorsal of which is better marked and of larger dimensions”, while also remarking the biting surface lacks denticles.

*Selenosteus brevis* – Changed from “absent” to “present”. Dean (1901: p. 96, pl. 6.37) described and figures prominent denticles in the holotype of *Selenosteus “kepleri”* (AMNH FF 7312), regarded as a synonym of *S. brevis* (Denison 1978).

*Enseosteus* spp. – Coded as “present” following Rücklin (2011: p. 35-36).

*Draconichthys elegans* – Changed from “absent” to “present”. Rücklin (2011: p. 54) describes a symphyseal tooth as present.

# Phylogeny Changes

- Added ANU V244 to the analysis. ANU V244 is thought to represent a buchanosteid closely related to, though seemingly non-conspecific with, *Buchanosteus confertituberculatus* (Hu et al. 2017: supplementary information). Because of this it is relevant to polarizing character-state changes near the most recent common ancestor of Eubrachythoraci. As *B. confertituberculatus* is the only buchanosteoid included in the parent analysis, there is little controversy over the potential position of this taxon.
- Added “*Dunkleosteus” magnificus* to the tree. *“Dunkleosteus” magnificus* has not been included in any phylogeny of the arthrodira, though it very clearly represents a dunkleosteid. This taxon is important because it displays vestigial adsymphyseal denticles at the base of the odontoids (Hussakof and Bryant 1918), thus making it relevant to discussions of character polarity within Dunkleosteoidea. *“D.” magnificus* was provisionally placed as Dunkleosteoidea incertae sedis, apical of *Eastmanosteus calliaspis* but not necessarily close to *D. terrelli*. This seems a reasonable compromise position given the varying taxonomic history of this species (Denison 1978, Dennis-Bryan 1987, Hussakof and Bryant 1918), at least until this taxon can be re-examined in more detail.

# Literature Cited

Boyle, J., and M. J. Ryan. 2017. New information on *Titanichthys* (Placodermi, Arthrodira) from the Cleveland Shale (Upper Devonian) of Ohio, USA. Journal of Paleontology 91(2):318–336.

Carr, R. K. 1994. A redescription of *Gymnotrachelus* (Placodermi: Arthrodira) from the Cleveland Shale (Famennian) of northern Ohio, U.S.A. Kirtlandia 48:3–21.

Carr, R. K. 1996. *Stenosteus angustopectus* sp. nov. from the Cleveland Shale (Fammenian) of northern Ohio with a review of selenosteid (Placodermi) systematics. Kirtlandia 49:19–43.

Carr, R. K., and W. J. Hlavin. 2010. Two new species of *Dunkleosteus* Lehman, 1956, from the Ohio Shale Formation (USA, Famennian) and the Kettle Point Formation (Canada, Upper Devonian), and a cladistic analysis of the Eubrachythoraci (Placodermi, Arthrodira). Zoological Journal of the Linnean Society 159(1):195–222.

Carr, R. K., H. Lelièvre, and G. L. Jackson. 2010. The ancestral morphotype for the gnathostome pectoral fin revisited and the placoderm condition. Pp. 107–122. *In* D. K. Elliott, J. G. Maisey, X. Yu, and D. Miao, eds. Morphology, Phylogeny and Paleobiogeography of Fossil Fishes. Verlag Dr. Friedrich Pfeil, München.

Dean, B. 1901. On two new arthrodires from the Cleveland Shale of Ohio. Memoirs of the New York Academy of Sciences 2(3):86–100.

Dean, B. 1909. Notes on a newly mounted *Titanichthys*. Memoirs of the American Museum of Natural History 9(5):270–271.

Denison, R. H. 1978. Placodermi. Pp. 1–128. *In* H.-P. Schultze, ed. Handbook of Palaeoichthyology. Gustav Fischer Verlag, Stuttgart.

Dennis-Bryan, K. 1987. A new species of eastmanosteid arthrodire (Pisces: Placodermi) from Gogo, Western Australia. Zoological Journal of the Linnean Society 90(1):1–64.

Dennis, K., and R. S. Miles. 1979a. Eubrachythoracid arthrodires with tubular rostral plates from Gogo, Western Australia. Zoological Journal of the Linnean Society 67(4):297–328.

Dennis, K., and R. S. Miles. 1979b. A second eubrachythoracid arthrodire from Gogo, Western Australia. Zoological Journal of the Linnean Society 67(1):1–29.

Dennis, K., and R. S. Miles. 1980. New durophagous arthrodires from Gogo, Western Australia. Zoological Journal of the Linnean Society 69(1):43–85.

Dennis, K., and R. S. Miles. 1981. A pachyosteomorph arthrodire from Gogo, Western Australia. Zoological Journal of the Linnean Society 73(3):213–258.

Dennis, K., and R. S. Miles. 1982. A eubrachythoracid arthrodire with a snubnose from Gogo, Western Australia. Zoological Journal of the Linnean Society 75(2):153–166.

Desmond, A. J. 1974. On the coccosteid arthrodire *Millerosteus minor*. Zoological Journal of the Linnean Society 54(4):277–298.

Dunkle, D. H. 1947. A new genus and species of arthrodiran fish from the upper Devonian Cleveland Shale. Kirtlandia 8(10):103–117.

Dunkle, D. H., and P. A. Bungart. 1940. On one of the least known of the Cleveland Shale Arthrodira. Kirtlandia 8(3):29–47.

Dunkle, D. H., and P. A. Bungart. 1945. A new arthrodiran fish from the upper Devonian Ohio Shales. Kirtlandia 8(8):85–95.

Dunkle, D. H., and P. A. Bungart. 1946. The antero-supragnathal of *Gorgonichthys*. American Museum Novitates 1316:1–10.

Dupret, V. 2010. Revision of the genus *Kujdanowiaspis* Stensiö, 1942 (Placodermi, Arthrodira, “Actinolepida”) from the Lower Devonian of Podolia (Ukraine). Geodiversitas 32(1):5–63.

Engelman, R. K. 2023. A Devonian fish tale: A new method of body length estimation suggests much smaller sizes for *Dunkleosteus terrelli* (Placodermi: Arthrodira). Diversity 15(3):318.

Engelman, R. K. 2024. Reconstructing *Dunkleosteus terrelli* (Placodermi: Arthrodira): A new look for an iconic Devonian predator. Palaeontologia Electronica 27(3):a44.

Fitzpatrick, A. N., A. M. Clement, and J. A. Long. 2024. Unique dental arrangement in a new species, *Groenlandaspis howittensis* (Placodermi, Arthrodira) from the Middle Devonian of Mount Howitt, Victoria, Australia. PeerJ 12:e18759.

Gardiner, B. G., and R. S. Miles. 1990. A new genus of eubrachythoracid arthrodire from Gogo, Western Australia. Zoological Journal of the Linnean Society 99(2):159–204.

Gardiner, B. G., and R. S. Miles. 1994. Eubrachythoracid arthrodires from Gogo, Western Australia. Zoological Journal of the Linnean Society 112(4):443–477.

Goujet, D. 1973. *Sigaspis*, un nouvel arthrodire du Dévonien inférieur du Spitzberg. Palaeontographica Abteilung A 143:73–88.

Goujet, D. 1975. *Dicksonosteus*, un nouvel Arthrodire du Devonien du Spitsberg. Colloque inteml C.N.R.S. 218:81–99.

Heintz, A. 1933. Revision of the Estonian Arthrodira, Part I: Family Homostiidae Jaekel. Archiv fur die Naturkunde Estland 1:1–114.

Hu, Y., J. Lu, and G. C. Young. 2017. New findings in a 400 million-year-old Devonian placoderm shed light on jaw structure and function in basal gnathostomes. Scientific Reports 7(1):7813.

Hussakof, L. 1906. Studies on the Arthrodira. Memoirs of the American Museum of Natural History 9(3):103–155.

Hussakof, L., and W. L. Bryant. 1918. Catalog of the fossil fishes in the Museum of the Buffalo Society of Natural Sciences. Bulletin of the Buffalo Society of Natural Sciences 12:1–198.

Jobbins, M., M. Rücklin, H. G. Ferrón, and C. Klug. 2022. A new selenosteid placoderm from the Late Devonian of the eastern Anti-Atlas (Morocco) with preserved body outline and its ecomorphology. Frontiers in Ecology and Evolution 10:969158.

Lelièvre, H. 1991. New information on the structure and the systematic position of *Tafilatichthys lavocati* (Placodermi, Arthrodira) from the Late Devonian of Tafilalt, Morocco. Pp. 121-130. Early vertebrates and related problems of evolutionary biology. Science Press, Beijing.

Lelièvre, H., R. Feist, D. Goujet, and A. Blieck. 1978. Les vertebres Devoniens de la Montagne Noire (Sud de la France) et leur apport a la phylogenie des pachyosteomorphes (Placodermes Arthrodires). Palaeovertebrata 17(1):1–26.

Lelievre, H., P. Janvier, and D. Goujet. 1981. Les vertébrés dévoniens de l'Iran central: IV: Arthrodires et ptyctodontes. Geobios 14(6):677–709.

Long, J. 1988. A new camuropiscid arthrodire (Pisces: Placodermi) from Gogo, Western Australia. Zoological Journal of the Linnean Society 94(3):233–258.

Long, J. 1994. A second incisoscutid arthrodire (Pisces, Placodermi) from the Late Devonian Gogo Formation, Western Australia). Alcheringa 18:59–69.

Long, J. A. 1990. Two new arthrodires (placoderm fishes) from the Upper Devonian Gogo Formation, Western Australia. Memoirs of the Queensland Museum 28:51–64.

Mark-Kurik, E. 1985. *Actinolepis spinosa* n. sp. (Arthrodira) from the Early Devonian of Latvia. Journal of Vertebrate Paleontology 5(4):287–292.

Miles, R. S. 1962. *Gemuendenaspis* n.gen., an Arthrodiran Fish from the Lower Devonian Hunsrückschiefer of Germany. Transactions of the Royal Society of Edinburgh 65(3):59-77.

Miles, R. S. 1966a. The Placoderm Fish *Rhachiosteus pterygiatus* Gross and its Relationships. Earth and Environmental Science Transactions of The Royal Society of Edinburgh 66(15):377–392.

Miles, R. S. 1966b. *Protitanichthys* and some other coccosteomorph arthrodires from the Devonian of North America. Swenska wetenskaps academiens handlingar 10(3):1–49.

Miles, R. S., and K. Dennis. 1979. A primitive eubrachythoracid arthrodire from Gogo, Western Australia. Zoological Journal of the Linnean Society 66(1):31–62.

Miles, R. S., and T. S. Westoll. 1963. Two new genera of coccosteid Arthrodira from the middle Old Red Sandstone of Scotland, and their stratigraphical distribution. Transactions of the Royal Society of Edinburgh 65(9):179–210.

Miles, R. S., and T. S. Westoll. 1968. The Placoderm Fish *Coccosteus cuspidatu*s Miller ex Agassiz from the Middle Old Red Sandstone of Scotland. Part I. Descriptive Morphology. Transactions of the Royal Society of Edinburgh 67(9):373–476.

Miles, R. S., and E. I. White. 1971. The Holonematidae (placoderm fishes), a review based on new specimens of *Holonema* from the Upper Devonian of Western Australia. Philosophical Transactions of the Royal Society of London. B, Biological Sciences 263(849):101–234.

Newman, M. J., J. den Blaauwen, C. Burrow, and R. Jones. 2021. Earliest vertebrate embryos in the fossil record (Middle Devonian, Givetian). Palaeontology 64(1):21–30.

Rücklin, M. 2011. First selenosteid placoderms from the eastern Anti-Atlas of Morocco; osteology, phylogeny and palaeogeographical implications. Palaeontology 54(1):25–62.

Rücklin, M., J. A. Long, and K. Trinajstic. 2015. A new selenosteid arthrodire (‘Placodermi’) from the Late Devonian of Morocco. Journal of Vertebrate Paleontology 35(2):e908896.

Schultze, H.-P., and S. L. Cumbaa. 2017. A new Early Devonian (Emsian) arthrodire from the Northwest Territories, Canada, and its significance for paleogeographic reconstruction. Canadian Journal of Earth Sciences 54(5):461–476.

Stensiö, E. A. 1963. Anatomical studies on the arthrodiran head. Pt. 1. Preface, geological and geographical distribution, the organisation of the arthrodires, the anatomy of the head in the Dolichothoraci, Coccosteomorphi and Pachyosteomorphi. Kungligar Svenska Vetenskapakadamiens Handlingar 9:1–419.

Trinajstic, K., and M. Hazelton. 2007. Ontogeny, phenotypic variation and phylogenetic implications of arthrodires from the Gogo Formation, Western Australia. Journal of Vertebrate Paleontology 27(3):571–583.

Trinajstic, K., S. Sanchez, V. Dupret, P. Tafforeau, J. Long, G. Young, T. Senden, C. Boisvert, N. Power, and P. E. Ahlberg. 2013. Fossil musculature of the most primitive jawed vertebrates. Science 341(6142):160–4.

Vézina, D. 1988. *Plourdosteus canadensis* (Woodward 1892), un Arthrodire du Frasnien inférieur du Canada: contribution à l'étude morphologique et phylogénétique des Plourdosteidae (Vertebrata, Placodermi) du Dévonien moyen et supérieur. l'Université de Paris VII, Paris.

Zhu, Y.-A., and M. Zhu. 2013. A redescription of *Kiangyousteus yohii* (Arthrodira: Eubrachythoraci) from the Middle Devonian of China, with remarks on the systematics of the Eubrachythoraci. Zoological Journal of the Linnean Society 169(4):798–819.

Zhu, Y.-A., M. Zhu, and J.-Q. Wang. 2016. Redescription of *Yinostius major* (Arthrodira: Heterostiidae) from the Lower Devonian of China, and the interrelationships of Brachythoraci. Zoological Journal of the Linnean Society 176(4):806–834.
